# Beta cell secretion of miR-375 to HDL is inversely associated with insulin secretion

**DOI:** 10.1038/s41598-019-40338-7

**Published:** 2019-03-07

**Authors:** Leslie R. Sedgeman, Carine Beysen, Marisol A. Ramirez Solano, Danielle L. Michell, Quanhu Sheng, Shilin Zhao, Scott Turner, MacRae F. Linton, Kasey C. Vickers

**Affiliations:** 1 Department of Molecular Physiology and Biophysics, Vanderbilt University, Nashville, TN USA; 2 KineMed, Inc., Emeryville, CA USA; 3 Department of Medicine, Vanderbilt University Medical Center, Nashville, TN USA; 4 Department of Biostatistics, Vanderbilt University Medical Center, Nashville, TN USA

## Abstract

Extracellular microRNAs (miRNAs) are a new class of biomarkers for cellular phenotypes and disease, and are bioactive signals within intercellular communication networks. Previously, we reported that miRNAs are secreted from macrophage to high-density lipoproteins (HDL) and delivered to recipient cells to regulate gene expression. Despite the potential importance of HDL-miRNAs, regulation of HDL-miRNA export from cells has not been fully studied. Here, we report that pancreatic islets and beta cells abundantly export miR-375-3p to HDL and this process is inhibited by cellular mechanisms that promote insulin secretion. Small RNA sequencing and PCR approaches were used to quantify beta cell miRNA export to HDL. Strikingly, high glucose conditions were found to inhibit HDL-miR-375-3p export, which was dependent on extracellular calcium. Likewise, stimulation of cAMP was found to repress HDL-miR-375-3p export. Furthermore, we found that beta cell ATP-sensitive potassium channel (K_ATP_) channels are required for HDL-miRNA export as chemical inhibition (tolbutamide) and global genetic knockout (*Abcc8*^−/−^) approaches inhibited HDL-miR-375-3p export. This process is not likely associated with cholesterol flux, as gain-of-function and loss-of-function studies for cholesterol transporters failed to alter HDL-miR-375-3p export. In conclusion, results support that pancreatic beta cells export miR-375-3p to HDL and this process is inversely regulated to insulin secretion.

## Introduction

Islets of Langerhans in the pancreas control systemic energy homeostasis primarily through two cell types: insulin-producing beta cells and glucagon-producing alpha cells. In response to high glucose, beta cells secrete insulin in a process known as glucose-stimulated insulin secretion (GSIS); however, chronic exposure to supraphysiological concentrations of glucose, e.g. conditions of systemic insulin resistance, can result in damage to the beta cell, and the development of Type 2 Diabetes (T2D)^1,2^. HDL have many beneficial properties in various biological processes that underlie pancreatic beta cell integrity and function, including enhancing GSIS^3,4^. HDL’s regulation of beta cell integrity and insulin secretion have been reported to be both dependent and independent of cholesterol transporters - ATP binding cassette transporter A1 (ABCA1) and scavenger receptor BI (SR-BI)^4,5^. HDL also have a wide-variety of alternative functions in many different cell-types conferred in part through the transport of non-cholesterol cargo, e.g. miRNAs^6–8^, suggesting there may be a relationship between insulin secretion and HDL-miRNAs.

miRNAs are small non-coding RNAs (18–22 nts in length) that post-transcriptionally regulate gene expression and are key factors in many (patho)physiologies^9^. In pancreatic beta cells, miRNAs have emerged as critical regulators of insulin secretion and cellular proliferation^10,11^. miR-375-3p is highly expressed in pancreatic islets and beta cells^12,13^, and is a key regulator of beta cell proliferation and function. Extracellular miRNAs are also detected in plasma, where they are protected from RNase digestion by protein and/or lipid complexes^8,14,15^. Recently, miR-375-3p was also found to be released from beta cells in exosomes^16^, suggesting that miR-375-3p may have both cellular and extracellular roles. Nevertheless, extracellular miR-375-3p is not limited to just exosomes, as we have previously reported that HDL also transport miR-375-3p^8^. Furthermore, we have previously demonstrated that HDL can transfer miRNAs, including miR-375-3p, to recipient cells, e.g. hepatocytes^8^. HDL-transferred miRNAs are also functional in recipient cells, as we have previously demonstrated that HDL-miRNAs delivered to coronary artery endothelial cells regulate key inflammatory genes, thus conferring, in part, HDL’s anti-inflammatory properties^6^. Collectively, our previous studies support that HDL transports miRNAs in circulation, that HDL-miRNA signatures are altered in metabolic disease, and that HDL-miRNAs serve as cell-to-cell signals within potential intercellular communication networks. Despite these findings, very little is understood about how cells export miRNAs to HDL.

Here, we demonstrate that pancreatic islets and beta cells export miRNAs, specifically miR-375-3p, to HDL and report key insights into processes that regulate export. Remarkably, contrary to exosomes in which miR-375-3p export was increased under cellular conditions that promote with insulin secretion^16^, we found that beta cell miR-375-3p export to HDL is suppressed by these same mechanisms that promote insulin secretion. Multiple conditions that stimulate insulin secretion from pancreatic beta cells - high glucose, tolbutamide, and persistent membrane depolarization - inhibited miRNA export to HDL. Moreover, we found that high glucose inhibition of HDL-miR-375-3p export required extracellular calcium, which is also critical for insulin secretion, thus further establishing the negative relationship between insulin secretion and HDL-miR-375 export. Nonetheless, despite the importance of cholesterol flux for insulin secretion from beta cells, miRNA export to HDL was found to be independent of cholesterol transporters. Collectively, these findings establish that the pancreatic beta cells contribute to extracellular miRNAs on circulating HDL and that beta cell miRNA export is inversely regulated by the cellular processes that promote insulin secretion.

## Results

### Pancreatic beta cells export miRNAs to HDL

**Figure 1:**
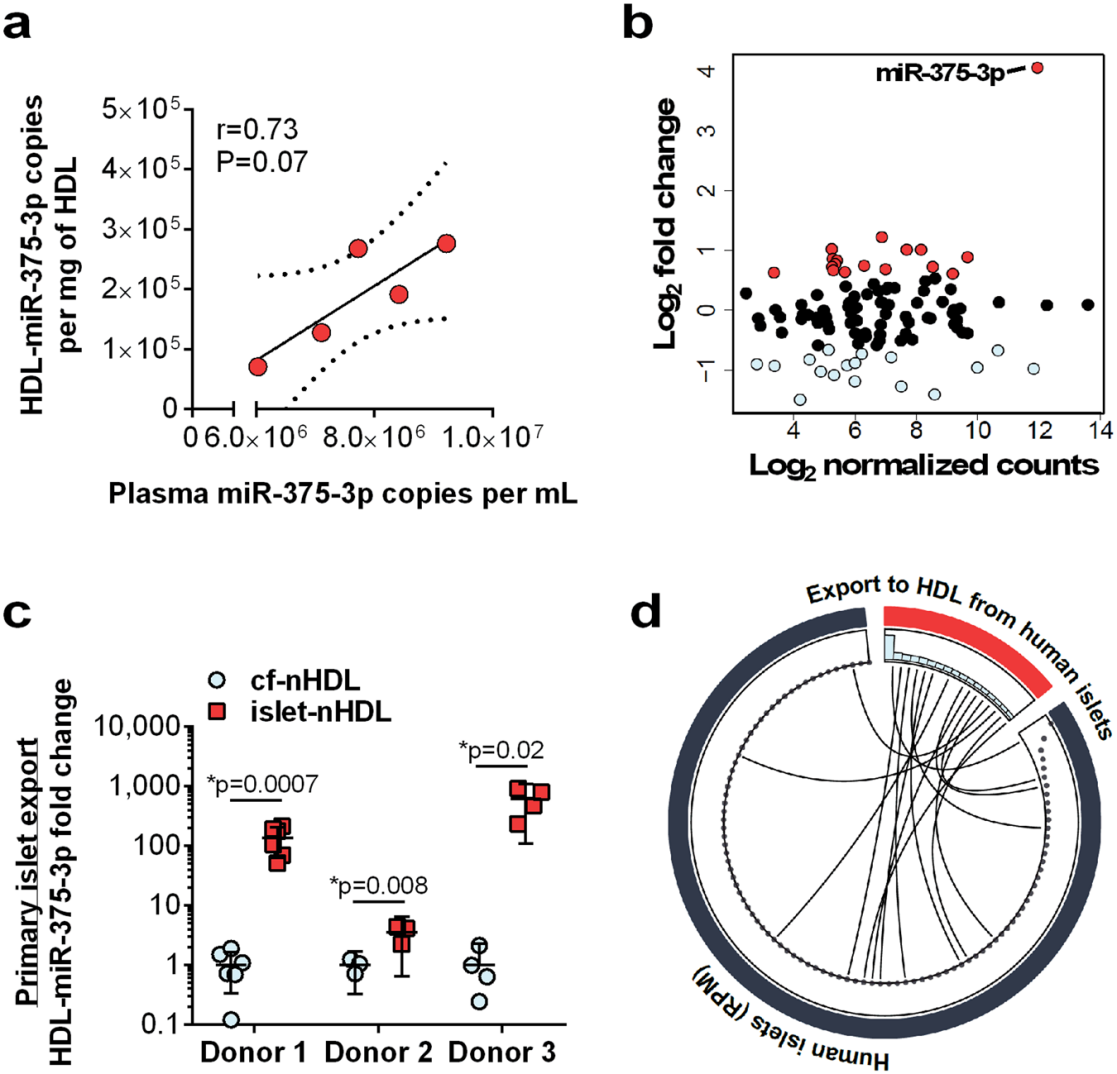
Pancreatic islets export miR-375-3p to HDL. (**a**) Quantification of miR-375-3p in plasma and HDL from WT mice. Correlation of plasma miR-375-3p levels and HDL-miR-375-3p levels. n = 5; Linear regression analysis. (**b**) MA plot of miRNA changes between cf-nHDL and islet-nHDL. n = 1 human donor. (**c**) miR-375-3p levels on cf-nHDL and islet-nHDL from three islet preps from individual donors. Donor 1: n = 6, donor 2: n = 3, donor: 3, n = 4; mean ± 95% CI; Two-tailed t-test. (**d**) Circos depicting top 100 islet miRNAs from 1 human donor (blue) and exported miRNAs from the same islets. sRNA-seq; dots represent RPM in islets and blue bars depict log_2_ HDL-miRNA fold change in islet-nHDL compared to cf-nHDL.

High-throughput sRNA-seq was used to quantify miRNAs on HDL from humans, and miR-375-3p was identified as a top-ranked HDL-miRNA (Table SI). To quantify the concentration of miR-375-3p on HDL, miR-375-3p levels were measured by quantitative PCR (qPCR) using 1 mg of HDL from human and mouse HDL isolated by size-exclusion chromatography (SEC) or density-gradient ultracentrifugation (DGUC) followed by SEC. We detected 10^5^–10^6^ copies/mg HDL (total protein) for miR-375-3p in both mouse and human samples (Fig. S1a). Furthermore, stoichiometric analysis of miR-375-3p levels on HDL and in plasma, suggests that HDL-miR-375-3p levels contribute to approximately 3.7–6.0% of the entire plasma pool of miR-375-3p (Fig. S1a). Moreover, we found that plasma and HDL miR-375-3p levels were strongly correlated. Based on miR-375’s link to beta cell biology and our previous study demonstrating that miR-375-3p was highly abundant on HDL, we hypothesized that pancreatic islets and beta cells contribute miR-375-3p to HDL. To test this hypothesis, we developed an HDL-miRNA export assay to measure miRNA efflux to HDL. Briefly, primary islets or beta cell lines were incubated with serum-free medium containing 1 mg/mL native HDL (islet-nHDL or INS-1-nHDL) for 24 h, or in cell free conditions (cf-nHDL). We then re-isolated nHDL from the culture media by SEC or apolipoprotein A-I immunoprecipitation (apoA-I-IP). To determine if primary human islets export miRNAs to HDL *ex vivo*, miRNA levels were quantified (by sRNA-seq) on cf-nHDL and on islet-nHDL, and differential expression analysis identified that 17 miRNAs were detected at levels >1.5 fold on HDL after incubation with islets compared to cf-nHDL (Fig. 1b and Table SII). Strikingly, miR-375-3p was found to be the only miRNA that was detected with a large fold change and at high abundance. Real-time PCR was used to confirm that miR-375-3p levels were significantly increased (donor 1: 135-fold, p = 0.0007; donor 2: 3.5-fold, p = 0.008; donor 3: 609-fold, p = 0.02) on nHDL after incubation with islets from three individual islet donors (Fig. 1c). Other top exported miRNAs (Table SII), let-7d-5p, miR-126-5p, miR-183-5p, miR-223-3p were measured by real-time PCR, and only miR-183-5p was found to be exported to HDL, albeit at much lower levels than miR-375-3p (Fig. S1b). To assess the specificity of human islet miRNA export to HDL, miRNA profiles from sRNA sequencing of the human islets from donor 1 and their exported HDL-miRNAs were linked (Fig. 1d, Tables SII and III). Most interestingly, we found that only 17 of the 1491 miRNAs detected in islets were exported to nHDL (>1.5-fold islet-nHDL vs. cf-nHDL levels), suggesting that miRNA export to nHDL is likely to be specific, and not simply reflecting cellular miRNA concentrations (Fig. 1d).

**Figure 2:**
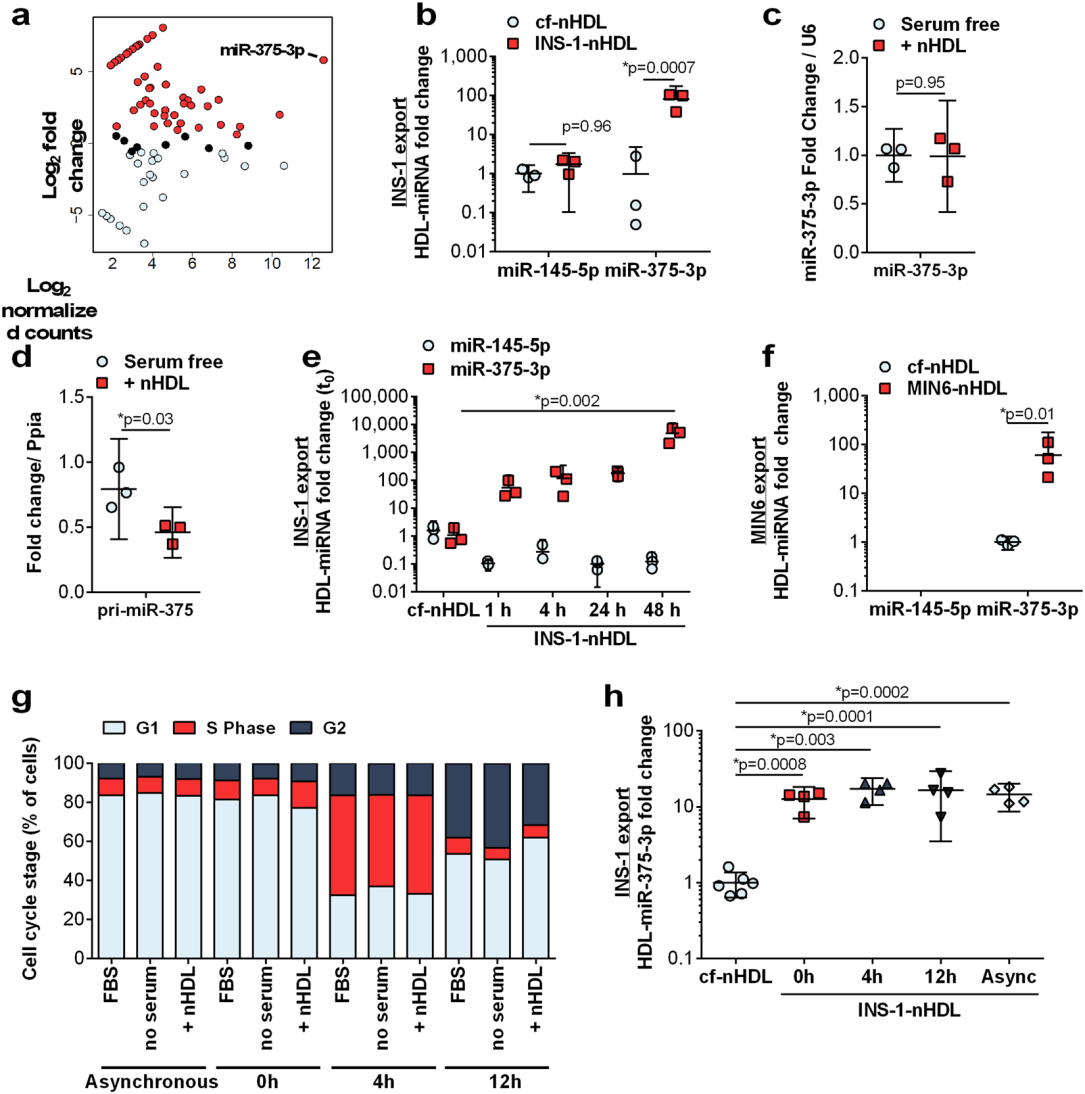
INS-1 (832/13) beta cells export miR-375-3p to HDL. (**a**) MA plot of miRNA changes between cf-nHDL and INS-1-nHDL. n = 1. (**b**) HDL miR-145-5p and miR-375-3p levels on cf-nHDL and INS-1-nHDL. n = 3; mean ± 95% CI; two-tailed t-test. (**c**,**d**) INS-1 cellular levels of (**c**) mature miR-375-3p and (**d**) primary-miR-375. n = 3; mean ± 95% CI; two-tailed t-test. (**e**) INS-1 miR-145-5p and miR-375-3p export to nHDL: cf-nHDL and INS-1-nHDL after 1, 4, 24, or 48 h after incubation with INS-1 cells. n = 3; mean ± 95% CI; One-way ANOVA with Bonferroni post-test, alpha = 0.05. (**f**) HDL miR-145-5p and miR-375-3p levels on cf-nHDL and INS-1-nHDL. n = 3; mean ± 95% CI; two-tailed t-test. (**g**) Percentage of INS-1 cells in G1, S and G2 phase determined by flow cytometry quantification of propidium iodide stain. n = 4; mean. (**h**) Export of miR-375-3p to nHDL after cell cycle synchronization and enrichment in G1 (0 h), S (4 h) and G2 (12 h) phase. n = 4; mean ± 95% CI; One-way ANOVA with Bonferroni post-test, alpha = 0.05.

To determine if pancreatic beta cells export miR-375-3p and other miRNAs to HDL, HDL-miRNA export assays were performed in rat INS-1 832/13 cells and sRNA-seq was used to quantify miRNAs on cf-nHDL and INS-1-nHDL. Strikingly, the levels of 61 miRNAs were increased >1.5-fold on INS-1-nHDL compared to cf-nHDL. (Fig. 2a and Table SIV). Similar to our findings of islet exported miRNAs (Fig. 1b), miR-375-3p export was robust and distinct from other miRNAs. Real-time PCR was used to confirm that INS-1 cells exported miR-375-3p to nHDL (81.48-fold, p = 0.0007) (Fig. 2b). Furthermore, miR-145-5p was used as a negative control, as we found that miR-145-5p was consistently not exported to INS-1-nHDL by real-time PCR (Fig. 2b). We quantified additional exported miRNAs by qPCR, miR-16-5p, miR-107-3p, miR-30d-5p, miR-182-5p, miR-21-5p, miR-27b-3p, miR-25-3p, miR-132-3p, miR-22-3p (Fig. S2). Similar to our findings in islets, only one miRNA, miR-132-3p was found to be significantly exported (p = 0.01) and at much lower levels than miR-375-3p (Fig. S2). Based on the striking observation that miR-375-3p is the most abundantly exported miRNA, we next investigated how its export is regulated.

miR-375-3p regulates beta cell development, and its levels have been found to be inversely associated with insulin secretion^12,17,18^. To determine if beta cell export of miR-375-3p to HDL reduces cellular miR-375-3p levels, mature miR-375-3p levels were quantified by real-time PCR in INS-1 cells treated with nHDL for 24 h. Cellular levels of mature miR-375-3p were not altered by nHDL treatments (Fig. 2c), suggesting that export of miR-375-3p to HDL is not likely a mechanism for the regulation or depletion of cellular miRNA levels. Nevertheless, miR-375 transcription was found to be inhibited by nHDL treatments, as the levels of the primary miR-375 transcript (pri-miR-375) were significantly decreased (p = 0.029) in INS-1 cells treated with nHDL compared to untreated cells (Fig. 2d). To determine the temporal dynamics of beta cell HDL-miR-375-3p export, INS-1-nHDL was purified at multiple time-points. We found that miR-375-3p levels steadily increased on INS-1-HDL over 24 h – 54-fold at 1 h, 116.3-fold at 4 h, 180.5-fold at 24 h, and 4,874.2–fold at 48 h compared to cf-nHDL levels - whereas miR-145-5p was not exported to HDL at any time-point (Fig. 2e). Cellular miR-375-3p levels were unaffected at any time-point, and miR-145-5p levels were considerably decreased at 24 h. (Fig. S3). To confirm that beta cell miR-375-3p export to HDL is not limited to rat INS-1 cells, HDL-miRNA export assays were also performed in mouse MIN6 cells, and we found that MIN6 cells also export miR-375-3p (46.19-fold), but not miR-145-5p, to nHDL (Fig. 2f). These results strongly support that pancreatic islets and INS-1 cells robustly and selectively export miR-375-3p to HDL.

Unlike native beta cells in the islets which are not proliferative, INS-1 and MIN6 cells are mitotically active^19^. We found that in unsynchronized INS-1 cells, approximately 77% of cells are in G1 phase, whereas approximately 6–8% of cells are in S and G2 phases (Figs 2g and S4). We next tested whether INS-1 cells that are enriched in G1 (0 h), S (4 h) or G2 (12 h) phases differentially export miR-375-3p to HDL. Of note, we did not observe any differences in cell cycle states when cells were treated with FBS, serum free, or serum free + nHDL media for the last 4 h of the experiments (Figs 2g and S4). Most importantly, we found that miR-375-3p levels on INS-1-nHDL are not affected by the cell cycle (Fig. 2h), whereas cellular levels of miR-375-3p were found to be increased at 0 h (p < 0.0001) compared to asynchronous cells (Fig. S5). Pri-miR-375 levels were not altered across the time points (Fig. S5). Due to the observation that despite the differences in proliferation between islets and INS-1 cells, miR-375-3p is the main miRNA exported from both islets and INS-1 cells at high levels, we sought to characterize whether miR-375-3p export is regulated by insulin secretion.

### Insulin secretion suppresses beta cell miRNA export to HDL

**Figure 3:**
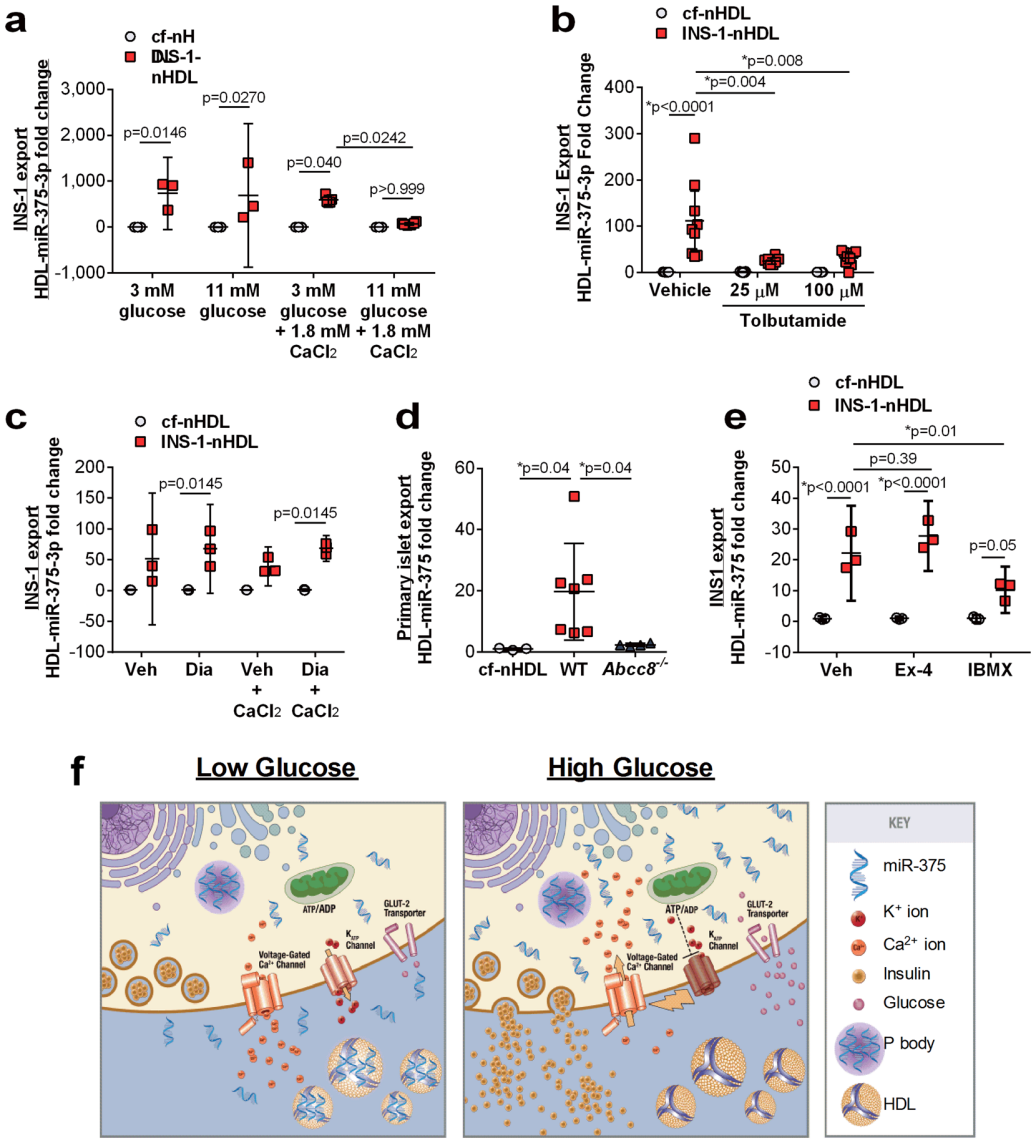
Stimulation of insulin secretion blocks INS-1 export of miR-375-3p to HDL. (**a**) miR-375-3p levels on cf-nHDL and INS-1-nHDL after INS-1 cell treatment with 3 mM or 11 mM D-glucose in the presence or absence of 1.8 mM CaCl_2_. n = 3–6; mean ± 95% CI; One-way ANOVA with Bonferroni post-test, alpha = 0.05. (**b**) HDL-miR-375-3p levels on cf-nHDL and INS-1-nHDL from INS-1 cells treated with tolbutamide for 2 h. n = 7–9; mean ± 95% CI; One-way ANOVA with Bonferroni post-test, alpha = 0.05. (**c**) miR-375-3p levels on cf-nHDL and INS-1-nHDL after INS-1 cell treatment with vehicle or diazoxide (DIA) in the presence or absence of 1.8 mM CaCl_2_. n = 3; mean ± 95% CI; One-way ANOVA with Bonferroni post-test, alpha = 0.05.(**d**) miR-375-3p levels on cf-nHDL and islet-nHDL from mouse WT (wildtype) or SUR1 KO (*Abcc8*^−/−^) mice. n = 3; mean ± 95% CI; One-way ANOVA with Bonferroni post-test, alpha = 0.05. (**e**) HDL-miR-375-3p levels on cf-nHDL and INS-1-nHDL from INS-1 cells treated with exendin-4 (ex-4) or IBMX for 3 h. n = 3; mean ± 95% CI; One-way ANOVA with Bonferroni post-test, alpha = 0.05. (**f**) Schematic depicting miRNA export to HDL in low glucose (left panel), and suppression of export in high glucose (right panel).

Pancreatic beta cells maintain systemic energy homeostasis through glucose sensing and insulin secretion; therefore, we sought to determine if the cellular mechanisms that control insulin secretion (i.e. GSIS) also regulate miRNA export to HDL. To determine the impact of high glucose conditions on beta cell miR-375-3p export to HDL, export assays were completed in INS-1 cells with RPMI media containing normal (3 mM) or high (11 mM) glucose levels. In these assays, we failed to find a difference in HDL-miR-375-3p export between the two conditions (Fig. 3a). However, RPMI medium is hypocalcemic (0.45 mM Ca(NO_3_)_2_) and GSIS requires extracellular calcium for membrane depolarization. Therefore, we tested whether high glucose (11 mM) media supplemented with 1.8 mM CaCl_2_ alters beta cell miR-375-3p export to HDL. The addition of 1.8 mM CaCl_2_ to the medium raises the extracellular levels to physiological levels, allowing for a rise in intracellular Ca^2+^ in response to high glucose stimulation. Remarkably, in the presence of extracellular Ca^2+^, high glucose conditions suppressed beta cell miR-375-3p export to nHDL (p = 0.0242 between INS-1-nHDL 3 mM glucose +1.8 mM CaCl_2_ and 11 mM glucose +1.8 mM CaCl_2_) (Fig. 3a). In response to high glucose, beta cells trigger a series of cellular processes that result in the secretion of insulin, including the closure of ATP-sensitive potassium channels (K_ATP_), depolarization of the plasma membrane, and influx of extracellular calcium through voltage-gated calcium channels^20^. To determine whether the observed inhibition of HDL-miR-375-3p export by high glucose conditions is regulated by cellular mechanisms that regulate insulin secretion, the role of K_ATP_ channels in miRNA export to HDL was investigated. HDL-miRNA export assays were performed in INS-1 cells treated with a chemical inhibitor of the K_ATP_ channel (tolbutamide)^21^, which results in depolarization of the plasma membrane. Strikingly, tolbutamide treatment resulted in a significant decrease in beta cell miR-375-3p export to nHDL (p = 0.0.004 INS-1-nHDL vehicle compared to INS-1-nHDL 25 μM tolbutamide; p = 0.008 INS-1-nHDL vehicle compared to INS-1-nHDL 100 μM tolbutamide) (Fig. 3b), without any changes in cellular miR-375-3p levels (Fig. S6). Conversely, diazoxide (Dia), a K_ATP_ channel activator, prevented the suppression of miR-375-3p export to HDL observed with Veh + CaCl_2_ (p = 0.7374 between cf-nHDL and INS-1-nHDL in Veh + CaCl_2_; p = 0.0145 between cf-nHDL and INS-1-nHDL in Dia + CaCl_2_) (Fig. 3c). Diazoxide also failed to alter cellular levels of mature miR-375-3p in INS-1 cells with or without CaCl_2_ (Fig. S6). In addition, we confirmed that both high glucose conditions and tolbutamide treatments stimulated insulin secretion from INS-1 cells, whereas diazoxide inhibited insulin secretion under high glucose conditions (Fig. S6).

The K_ATP_ channel is a hetero-octameric protein composed of four SUR1 and four Kir6.2 subunits, and deletion of either channel component results in incomplete trafficking to the plasma membrane and complete loss of channel activity^22^. To replicate our chemical inhibition findings using an orthogonal approach, we tested whether genetic deletion of a K_ATP_ channel subunit in mice (sulfonylurea receptor 1, SUR1 (*Abcc8*^−/−^)) impacts pancreatic islet miR-375-3p export to nHDL. Primary islets were isolated from *Abcc8*^−/−^ mice (Fig. S7), and miR-375-3p export to HDL was quantified *ex vivo*, as described above. In agreement with our *in vitro* studies, loss of the K_ATP_ channel in SUR1 knockout islets, significantly reduced miR-375-3p export to nHDL *ex vivo* (p = 0.0363 between WT and *Abcc8*^−/−^ islet-n-HDL) (Fig. 3d). K_ATP_–deficiency in mice (*Abcc8*^−/−^) has been reported to cause calcium-associated changes in the beta cell transcriptome^23^; therefore, we confirmed that the observed loss of HDL-miR-375-3p export was not merely due to decreased miR-375-3p expression in islets isolated from *Abcc8*^−/−^ mice compared to WT mice. In fact, miR-375-3p levels were found to be increased, not decreased, in *Abcc8*^−/−^ islets (Fig. S7). These findings suggest that miR-375-3p export to nHDL is inverse to insulin secretion. To further test this association, the role of cAMP was investigated.

As a second messenger molecule, cAMP enhances GSIS in beta cells through both protein kinase A dependent and independent mechanisms that regulate the exocytotic machinery and membrane depolarization^24,25^. A rise in cellular cAMP levels can be triggered by increased intracellular Ca^2+^ concentrations or through hormone signaling, e.g. glucagon-like-peptide 1 (GLP-1). cAMP also alters gene expression and recently, cAMP has been shown to repress transcription of pri-miR-375^26^. Therefore, we investigated whether miR-375-3p export to nHDL is regulated by cAMP. Two different compounds were used to stimulate cAMP in INS-1 cells, exendin-4 (ex-4), a GLP1R agonist, and IBMX, a phosphodiesterases inhibitor. As a positive control, the expression of c-fos was quantified, as this gene was previously reported to be increased with ex-4 and IBMX treatments^26,27^. IBMX treatments, but not ex-4, increased *Fos* mRNA levels in INS-1 cells (p < 0.0001), perhaps due to low level of expression of GLP1R in INS-1 cells^28^ (Fig. S8). We found that pri-miR-375, but not mature miR-375-3p levels were down-regulated in INS-1 cells treated with ex-4 or IBMX in serum-free media + nHDL (Fig. S8). Most interestingly, IBMX, but not ex-4, was found to repress miR-375-3p export to nHDL (p = 0.0098) (Fig. 3e). These results further support a model in which stimulation of GSIS from beta cells, either through glucose, membrane depolarization, or cAMP, inhibit miR-375-3p export to nHDL. Furthermore, these results established an inverse link between beta cell miRNA export to HDL and insulin secretion (Fig. 3f).

### Beta cell HDL-miRNA export is independent of cholesterol flux

**Figure 4:**
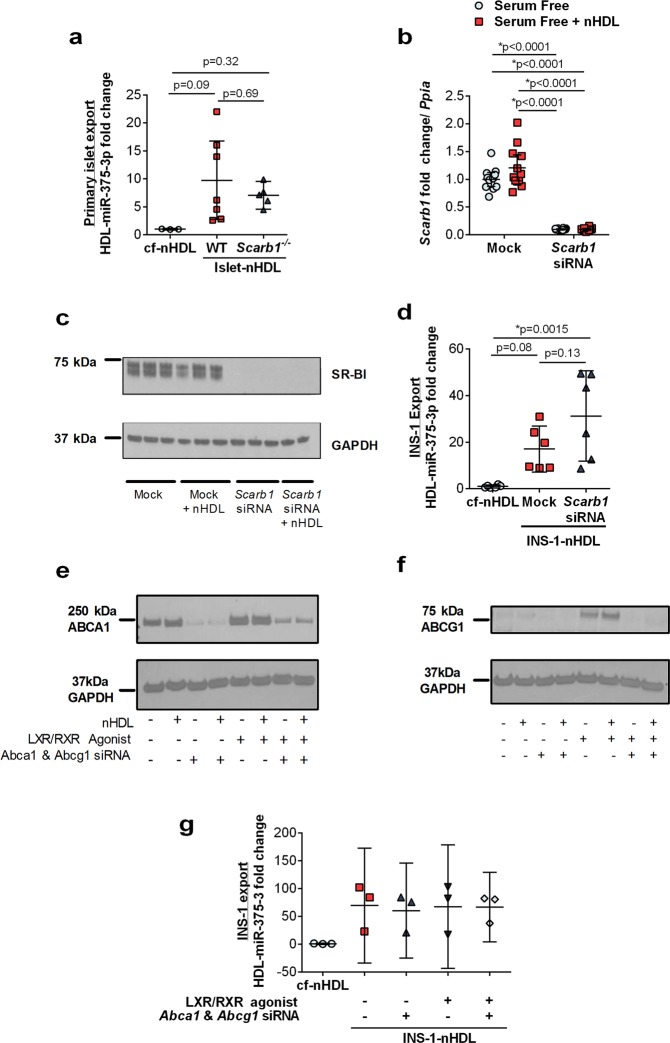
Beta cell miR-375-3p export to HDL does not require cholesterol transporters. (**a**) miR-375-3p levels on cf-nHDL and islet-nHDL from mouse WT (wildtype) or SR-BI KO (*Scarb1*^−/−^) mice. n = 3; mean ± 95% CI; One-way ANOVA with Bonferroni post-test, alpha = 0.05. (**b**) INS-1 cellular levels of *Scarb1* mRNA and (**c**) SR-BI protein (western blotting) after transfection with mock or 50 nM siRNA against *Scarb1*. Cropped images are of the same blot at different exposures. Full blots are available in Fig. S10. n = 12; mean ± 95% CI; One-way ANOVA with Bonferroni post-test, alpha = 0.05. (**d**) miR-375-3p levels on cf-nHDL and INS-1-nHDL after knockdown of *Scarb1*; cells were transfected with mock or 50 nM *Scarb1* siRNA. n = 6; mean ± 95% CI; One-way ANOVA with Bonferroni post-test, alpha = 0.05. (**e**) ABCA1 and (**f**) ABCG1 protein (western blotting) after transfection with mock or 50 nM siRNA against *Abca1* and *Abcg1*, in the presence or absence of TO901317 (LXR agonist) and 9-cis-retinoic acid (RXR agonist). Representative of n = 3. Cropped images are of the same blots at different exposures. Full blots are available in Fig. S10. (**g**) miR-145-5p and miR-375-3p levels on cf-nHDL and INS-1-nHDL after INS-1 cell transfection with mock or 50 nM siRNAs against *Abca1* and *Abcg1* and/or LXR/RXR agonists. n = 6; mean ± 95% CI; One-way ANOVA with Bonferroni post-test, alpha = 0.05.

Previously, studies have demonstrated that HDL enhances beta cell insulin secretion which requires cholesterol transporters^4^. Based on these findings, we sought to examine the roles of HDL’s primary receptor, scavenger receptor BI (SR-BI), and key cholesterol transporters, ATP-binding cassette transporter A1 (ABCA1) and ATPB-binding cassette transporter G1 (ABCG1), in regulating beta cell miRNA export to nHDL. SR-BI is a bidirectional transporter of cholesterol and lipids, and mediates HDL-induced cell signaling^29,30^. We have previously demonstrated that HDL-miRNA delivery to recipient hepatocytes was dependent upon SR-BI^8^. SR-BI is also expressed in pancreatic beta cells and could, therefore, directly transport miRNAs to nHDL or indirectly facilitate HDL-induced cell signaling promoting miRNA export. To determine if SR-BI-deficiency in mouse islets aids in trafficking miR-375-3p to nHDL, pancreatic islets were collected from *Scarb1*^−/−^ (SR-BI KO) and c57Bl/6 wild-type mice (WT), and incubated with nHDL *ex vivo* (Fig. S9). Surprisingly, islets from both SR-BI KO and WT mice were found to export miR-375-3p to nHDL and we found no difference between islet genotype (p = 0.6876 between WT and *Scarb1*^−/−^ islet-nHDL) (Fig. 4a). Moreover, we tested whether beta cell SR-BI regulates HDL-miR-375-3p export *in vitro*; siRNAs were used to knockdown SR-BI expression in INS-1 cells, which was confirmed at the mRNA and protein levels by real-time PCR and western blotting, respectively (Fig. 4b,c). Similar to our findings in primary islets *ex vivo*, SR-BI was not required for miR-375-3p export to nHDL from INS-1 cells (p = 0.134 between mock and *Scarb1* siRNA INS-1-nHDL) (Fig. 4d).

We next sought to investigate the role of cholesterol transporters ABCA1 and ABCG1 in regulating miRNA export to HDL. ABCA1 and ABCG1 mediate cholesterol and lipid efflux to discoidal nascent HDL and spherical HDL particles, respectively^31^. ABCA1 is also a key mediator of HDL-induced anti-inflammatory cell signaling. We have previously reported that liver-X-receptor (LXR) activation, which increases ABCA1 and ABCG1 expression, failed to alter miR-223-3p export from macrophages to nHDL^8^. Nonetheless, ABCA1 and/or ABCG1 might regulate miR-375-3p export to nHDL in pancreatic beta cells; therefore, siRNAs were used to knockdown ABCA1 and ABCG1 expression in INS-1 cells, which was confirmed by loss of mRNA and protein levels (Figs 4e,f and S9). Due to low basal levels of ABCG1 expression in beta cells, we also studied the effect of transporter over-expression using LXR/RXR agonists which promote the transcription of *Abca1* and *Abcg1* (TO901317, LXR agonist; 9-cis-retinoic acid, RXR agonist) (Figs 4e,f and S9). HDL-miRNA export assays were performed in conditions of dual *Abca1* and *Abcg1* knockdown or over-expression; however, neither silencing, nor over-expression of these cholesterol transporters had any effect on beta cell HDL-miR-375-3p export (Fig. 4g). Thus, SR-BI, ABCA1, and ABCG1 do not likely regulate HDL-miR-375-3p export from pancreatic beta cells. Combined, these results support a model in which beta cell miR-375-3p export and cholesterol efflux to HDL are mediated by distinct transporters and/or pathways.

## Discussion

HDL particles have many diverse beneficial properties, including anti-inflammatory and anti-oxidant capacities^3^. In the beta cell, HDL promotes cell integrity and proper function, e.g. protection against cellular toxicity and maintenance of GSIS^3^. Although most of the beneficial properties of HDL are attributed to cholesterol metabolism, HDL transports many types of non-cholesterol cargo, including proteins, vitamins, bioactive lipids, and non-coding sRNAs, which also likely contribute to HDL’s beneficial properties^7^. Previously, we reported that HDL transport miRNAs and deliver them to recipient cells where they regulate target gene expression^6,8^. Nevertheless, very little is known about the origin of HDL-miRNAs and the mechanisms of cellular miRNA export to HDL. In this study, we demonstrate that pancreatic islets and beta cells export selected miRNAs, e.g. miR-375-3p, to HDL. Using sRNA-seq, we profiled miRNA expression in pancreatic islets and on HDL. Comparisons of miRNAs detected in both HDL and islets showed considerable overlap. Based on this overlap, sRNA-seq was further used to identify the miRNAs that are exported from primary islets and beta cells to HDL *ex vivo* and *in vitro*, and we found that miR-375-3p is a highly and the most abundantly exported miRNA to HDL. Results suggest that beta cell miRNA export to HDL is inversely linked to insulin secretion. For example, beta cell miRNA export to HDL likely occurs during cellular conditions in which insulin secretion is low, as HDL-miR-375-3p export was inhibited under conditions that stimulate insulin secretion. Although we hypothesized that cholesterol transporters facilitate miRNA transport across the plasma membrane, we found that inhibition of SR-BI, ABCA1, and ABCG1 failed to alter beta cell miRNA export to HDL. Overall, these observations markedly advance our fundamental understanding of HDL-miRNA secretion.

Many of the most abundant miRNAs in pancreatic islets and beta cells were found circulating on HDL, including miR-375-3p. Due to miR-375-3p’s critical importance to beta cell integrity and function, extracellular circulating miR-375-3p levels have been reported as a biomarker of beta cell function^18,32^. miR-375-3p has previously been detected in plasma, as well as exosomes and microvesicles, prompting renewed interest in the role of miR-375-3p as an intercellular signaling molecule and for its utility as a biomarker of disease (and pre-disease)^16^. In patients with T2D, no clear consensus exists as to the regulation of extracellular miR-375-3p levels, as different studies have reported an increase, decrease, or no change in extracellular miR-375-3p levels in T2D human subjects and rodent models of T2D^18^. Nevertheless, in both humans with Type 1 Diabetes (T1D) and murine models of T1D, miR-375-3p was consistently found to be upregulated in plasma samples^33^. For this reason, circulating (plasma/serum) miR-375-3p has been proposed as a biomarker of beta cell death^32,34^. Despite reports that beta cells may release miR-375-3p during cell death and serum-free conditions used in our studies may promote some level of cell death, we did not observe substantial cell death in our cell culture conditions. Moreover, in this study, we found that beta cell miRNA export is selective for miR-375-3p, and that many of the other highly-abundant miRNAs in the beta cells and islets are not exported to HDL, as determined by sRNA-seq.

Due to the secretory phenotype of the endocrine beta cell, we sought to determine whether cellular mechanisms that control insulin secretion also regulate miRNA export to HDL. We found that while miR-375-3p was readily exported to HDL in low glucose conditions, export was inhibited at high glucose in the presence of physiologic calcium concentrations. In beta cells, under basal, low glucose conditions, the plasma membrane is hyperpolarized and insulin secretion is low. Increases in glucose concentrations above 5 mM result in glucose metabolism and a rise in the ATP/ADP ratio. ATP closes the K_ATP_ channels resulting in the depolarization of the plasma membrane. This activates voltage-gated Ca^2+^ channels, and leads to increased intracellular [Ca^2+^] levels which result in insulin granule secretion. Many pharmacologic agents have been developed to modulate this process: sulfonylureas promote insulin secretion by binding to the SUR1 subunit and closing the K_ATP_ channel, whereas diazoxide opens the channel^22,35^. Here, chemical inhibition of the K_ATP_ channel, independent of high glucose, significantly inhibited miR-375-3p export to HDL. Conversely, diazoxide in high glucose prevent the suppresion of miR-375-3p export caused by CaCl_2_. It should also be noted that we were able to readily detect GSIS in INS-1 cells, and tolbutamide also increased insulin secretion in low glucose conditions, whereas diazoxide inhibited insulin secretion in high glucose. In high glucose, tolbutamide did not further stimulate GSIS beyond the effect of high glucose. We further tested the requirement of the K_ATP_ channel using islets isolated from SUR1 (*Abcc8*^−/−^) knockout mice, and islets lacking the K_ATP_ channel had a decreased capacity to export miR-375-3p to HDL. We also found that IBMX, but not ex-4, suppressed miR-375-3p export to nHDL. Of note, IBMX resulted in a strong induction of c-fos, a cAMP responsive gene, whereas ex-4 did not, suggesting that a large increase in cAMP may be required in INS-1 cells to inhibit miR-375-3p export to HDL. Based on these observations, a few models can be proposed. Firstly, the K_ATP_ channel itself may be responsible for directly transporting miRNAs across the plasma membrane; however, the size of the channel is likely not large enough to facilitate miRNAs transport across the plasma membrane. Secondly, that K_ATP_ channel may indirectly aid the transport of miRNAs across the plasma membrane through interaction with a currently unknown membrane transporter, potentially through an allosteric regulatory mechanism. Thirdly, inhibition (closure) or absence of the K_ATP_ channel alters the cell polarization state and ion concentrations leading to insulin secretion, while indirectly affecting miRNA release or retention in the cell. Although our studies cannot distinguish between these three models, our findings suggest that ion dynamics likely are involved, as glucose-induced repression of HDL-miR-375-3p export also required extracellular calcium and occurred with elevated cAMP.

Although this is the first study to report critical regulators of beta cell miRNA export to HDL, a recent study found that beta cell secretion of exosomes containing miR-375-3p was increased under conditions that promoted insulin secretion, i.e. high glucose, arginine and KCl^16^. This observed discrepancy—decreased miRNA export to HDL compared to increased exosome miRNA release with GSIS—is in agreement with our previous study in which we reported that inhibition of the ceramide signaling pathway by GW4869 (attenuates exosome secretion) increased miRNA release to HDL^8^. Although further work is required to fully define the relationship between insulin secretion, miRNA export to HDL, and exosome secretion, results presented here and our proposed model support that beta cell miRNA export through exosomes and HDL occur through distinct routes under opposing cellular stimuli and regulation.

Based on the results presented here and in previous studies from our laboratory^8^, beta cell-derived miRNAs are likely transported by HDL in circulation, and thus, may regulate target genes in cells that take up HDL-miRNAs. We have previously demonstrated that miR-223-3p, a miRNA detected on HDL at comparable levels to miR-375-3p, is biologically active on HDL and regulates inflammatory target genes in recipient endothelial cells^6^, thus supporting a potential role for beta cell-derived HDL-miR-375-3p in intercellular gene regulation. Nevertheless, the functional impact of HDL-miR-375-3p on cell-to-cell communication networks remains to be determined. In exosomes, miR-375-3p has been reported to be the 3^rd^ most abundant miRNA in exosomes originating from pancreatic beta cells (following miR-709 and miR-1224)^36^,and miRNAs that are detected in exosomes at 8–10 times lower concentration than miR-375-3p, e.g. miR-15a, have been shown to have biological function in miRNA-mediated communication originating from beta cells^37^. For example, an exogenous miRNA, cel-miR-238, when expressed in pancreatic beta cells has been shown to be released into exosomes to promote apoptosis in recipient cells^36^. Similarly, exosomes released from human islets and beta cells have also been reported to be taken up by recipient dendritic cells^38^. Furthermore, INS-1 beta cells were demonstrated to release exosomes containing miR-15a, which was increased in high glucose conditions^37^. These INS-1 cell exosomes were demonstrated to target Akt3 and promote apoptosis in recipient retinal glial cells (Muller cells), thus representing a potential underlying mechanism of diabetic complication resulting from beta cell-originating intercellular communication^37^. Although these miRNAs have been demonstrated to have physiological function in cell-to-cell communication pathways, they are found in exosomes at concentrations much lower than miR-375-3p. In addition, a recent study by Chevillet *et al*., found that the levels of miR-375-3p carried in exosomes represent a small percentage (2.7%) of total plasma miR-375-3 levels, thus leaving 97.3% to be associated with other carriers, e.g. lipoproteins and RNA-binding proteins^39^.

Nevertheless, essential questions regarding the biological functions of HDL-bound miR-375-3p remain to be determined. HDL-miR-375-3p is abundantly detected in plasma, and likely regulates cell-to-cell communication in some capacity^8^. We hypothesize that HDL-miR-375 is transported from the beta cell to recipient cells where miR-375-3p regulates target gene expression; however, at this time the identity to the recipient cells/tissues are unknown. Furthermore, it is currently unknown whether HDL-miR-375-3p are delivered to distinct cells and tissues, and whether HDL-delivered miR-375-3p target different genes under distinct physiological conditions, i.e. fast vs. feeding states. miR-375-3p has been extensively studied in pancreatic beta cells, however, a few studies in other cell-types offer clues to the potential physiological effects of HDL-miR-375-3p. For example, miR-375-3p has been shown to negatively regulate osteogenesis, the process of bone formation, by targeting Wnt signaling proteins^40^. Wnt signaling is also involved in epithelial cell differentiation in the lung and rheumatoid arthritis (RA) pathogenesis^41^. miR-375-3p was also shown to target Frizzled 8, a component of canonical Wnt signaling, and inhibit epithelial cell differentiation in response to lung injury^41^. In fibroblasts, miR-375-3p suppression of Frizzled 8 inhibited disease pathogenesis in a rat model of RA^42^. In the brain, miR-375-3p is associated with neuroprotection in a rat model of cerebral ischemia/reperfusion^43^.

In summary, this study provides regulatory insight into the cellular process that affect miR-375-3p export to HDL from human and mouse islets and pancreatic beta cells. Results suggest that miR-375-3p export to HDL is inhibited by glucose-stimulation (high glucose), but only in the presence of extracellular (1.8 mM) Ca^2+^. Furthermore, through multiple different approaches, we demonstrate that miR-375-3p export to HDL occurs only under conditions of low insulin secretion, as demonstrated by modulating K_ATP_ channel activity and cAMP levels. Moreover, we found that miR-375-3p export to HDL was independent of cholesterol transporters—ABCA1, ABCG1 and SR-BI. Together, these results support that pancreatic beta cell miR-375-3p export to HDL is inversely regulated by the cellular mechanisms that control insulin secretion.

## Experimental Procedures

### HDL Isolation

nHDL used for cell culture studies was isolated from healthy volunteers at Vanderbilt University Medical Center, purchased from Interstate Blood Bank, or obtained from the Vanderbilt blood bank. nHDL was isolated from human plasma by density-gradient ultra-centrifugation (DGUC, 1.061–1.21 g/mL), 330,000 × *g* followed by extensive dialysis at 4 °C in 1X PBS. The study conformed to the guidelines set out in the Declaration of Helsinki and pertinent ethical regulations. The protocol was approved by the ethical committee of Vanderbilt University Medical Center (IRB# 151573) and all volunteers gave written informed consent for participation in the study. Pooled mouse plasma (50 ml) from random fed mice was purchased from Equitech-Bio, Inc. HDL was isolated from mouse and human plasma by DGUC, as described above for nHDL. For human HDL-RNA analysis, blood was collected from healthy blood donors (n = 10) at Vanderbilt University Medical Center under active IRB protocol. Immuno-affinity purification of HDL was achieved as previously described^8^ using goat-anti-human apoA-I antibodies conjugated to Sepharose-4B beads (Academy Biomedical Company). RNA was isolated from 100 ug of HDL protein (Norgen Biotek).

### Animal Experiments

All animal experimentation was approved by and carried out in accordance to the Vanderbilt Institutional Animal Care and Use Committee. All transgenic lines were backcrossed to a C57BL/6 background for more than 10 generations. Female *Abcc8*^*−/−*^ (*Abcc8*^*tm1*.*1Mgn*^) and wildtype C57B6J (WT) mice were used at 12 wks old^23^. *Scarb1*^+/−^ mice were obtained from The Jackson Laboratory and bred to generate *Scarb1*^−/−^ mice. Female *Scarb1*^−/−^ and WT mice were used at 8 wks old. For correlation of plasma and HDL miR-375, blood was collected from 5 12-week old male WT mice. HDL was isolated using size exclusion chromatography. HDL fractions were pooled and RNA was isolated from 1 mg of HDL protein. RNA was isolated from 50ul of plasma.

### HDL-miRNA Export Assays

Primary islets (human and rodent) or INS-1 cells were incubated with 1 mg/mL nHDL (isolated by DGUC) in serum-free media for 1–48 h and maintained at 37 °C with 5% CO_2_ for 24 h. Control nHDL was added to media and incubated in the absence of cells (cell free, cf) at 37 °C with 5% CO_2_ for 24 h. cf-nHDL and islet-nHDL or INS-1-nHDL were isolated from culture media by immunoprecipitation (IP) using goat-anti-human apoA-I antibodies conjugated to Sepharose-4B beads (Academy Biomedical Company). RNA was isolated from 100 µg of HDL total protein (Norgen Biotek).

### Primary Islet Culture

Primary human islets from three individual human donors were obtained through the Integrated Islet Distribution Program. Primary mouse islets were isolated from the transgenic mice described above, as previously described^44^. Primary islets (50) were added to each well in RPMI supplemented with 5.6 mM glucose, streptomycin (100 μg/ml), penicillin (100 U/ml), 10% LPDS (DGUC 1.21 g/ml, bottom fraction), and 1 mg/ml DGUC nHDL and maintained at 37 °C with 5% CO_2_ for 24 h. For a control group, nHDL was added to islet-free media and maintained at 37 °C with 5% CO_2_ for 24 h in cell-free conditions (cf-nHDL). Cf-nHDL and islet-nHDL were isolated by IP against apoA-I, and total RNA was isolated from 100 µg of nHDL. Total RNA was also isolated from mouse islets to confirm genotype.

### Cell Culture

MIN6 cells were cultured in DMEM supplemented with streptomycin (100 μg/ml), penicillin (100 U/ml) and 10% FBS. INS-1 832/13 cells were cultured in RPMI supplemented with streptomycin (100 μg/ml), penicillin (100 U/ml), 10% FBS, and INS-1 supplement which consists of 10 mM HEPES, 2 mM L-glutamine, 1 mM sodium pyruvate, and 0.05 mM β-mercaptoethanol. All cells were maintained at 37 °C with 5% CO_2_. For miRNA export assay, INS1 and MIN6 cells were plated at 2 × 10^5^ cells/mL for 24 h prior to HDL addition. Export experiments were performed at the following glucose concentrations: MIN6 cells – 5 mM D-glucose, INS1 cells – 11 mM glucose, except for experiment used for HDL-sequencing which was performed at 3 mM glucose. DGUC nHDL was added to cells at 1 mg/ml for 1–24 h. For tolbutamide studies, cells were washed with 1x HBSS for 2 h (0 mM glucose). Media was then added containing 3 mM glucose and either 25 µM, 150 µM tolbutamide or ethanol vehicle for 1 h. RPMI media was not supplemented with CaCl_2_ for these experiments. 1 mg/mL nHDL was added for an additional 2 h. For low and high glucose studies, cells were wash with 1x HBSS, and then media containing 3 mM or 11 mM glucose supplemented with 1.8 mM CaCl_2_ +/− 1 mg/ml nHDL was added for 24 h. For cAMP studies, INS-1 cells were pretreated with vehicle, 100 µM IBMX, or 100 nM exendin-4 for 1 h in complete INS-1 media. Cells were then switched to serum free media +/− nHDL supplemented with 100 µM IBMX, or 100 nM exendin-4 for an additional 2 h. nHDL was added to the media from each of the conditions, as described above, and maintained at 37 °C with 5% CO_2_ for 24 h (cf-nHDL). For diazoxide studies: Media was added containing 11 mM glucose and either DMSO (vehicle) or 200 μM diazoxide for 1 h +/− 1.8 Mm CaCl_2_. 1 mg/mL nHDL was added for an additional 2 h. Cf-nHDL and INS-1-nHDL were isolated by IP for apoA-I or SEC, and total RNA was isolated from 100–300 µg of nHDL.

### Cell Cycle Assays

Synchronization was performed as previously described^19^. Briefly, cells were plated in INS-1 RMPI media containing 10% FBS. Asynchronous cells were plated at the same time, but remained in INS-1 media for the remainder of the study. After 24 h, synchronized cells were switched to INS-1 media + 0.1% FBS for 56 h, at which point cells were treated with 2 µg/ml aphidicolin (Sigma-Aldrich) for an additional 12 h. Following aphidicolin treatment, media was changed to INS-1 media with 10% FBS and cells were collected at 0 h, 4 h, and 12 h for flow cytometry analysis of cell cycle phases and RNA content. To quantify miRNA export, media was changed to serum free media or serum free +1 mg/mL nHDL 4 h prior to the final time-point for each condition. For analysis of cell cycle phases by flow cytometry, cells were trypsinized and counted, and 5 × 10^5^ cells were washed with 1X PBS and fixed in 80% methanol (Sigma-Aldrich) at −20 °C overnight. Methanol was removed by centrifugation at 2000 × *g* for 10 min and cells were washed in 1X PBS twice. Cells were stained with 50 μg/ml propidium iodide (Invitrogen) in the presence of 50 μg/ml RNAse A (ThermoFisher) on ice for 2 h. DNA content was analyzed using the 3-laser BD LSRII at the Vanderbilt Flow Cytometry Shared Resource Core.

### Transfection Studies

Cells were plated at 2 × 10^5^ cells/mL for 24 h prior to transfection (48 h) with DharmaFECT 4 (Dharmacon). Transient transfections (50 nM) were conducted with siRNA against Scarb1 (ON-TARGETplus SMARTpool L-098018-02), against Abca1 (ON-TARGETplus SMARTpool L-098018-02), against Abcg1 (ON-TARGETplus SMARTpool L-098018-02).

### Insulin Secretion Assays

INS-1 cells were plated onto 24-well plates and were grown to confluency prior to assay. The standard tissue culture medium was switched to medium containing 5 mM glucose for 18 h. Insulin secretion was performed in HEPES balanced salt solution (HBSS) (114 mM NaCl, 4.7 mM KCl, 1.2 mM KH_2_PO_4_, 1.16 mM MgSO_4_, 20 mM HEPES, 2.5 mM CaCl_2_, 25.5 mM NaHCO_3_ and 0.2% bovine serum albumin, pH 7.2). Cells were washed with HBSS containing 3 mM glucose, followed by a 2 h incubation in the same buffer. Insulin secretion was then measured in static incubations of HBSS containing 3 mM or 15 mM glucose containing 200 μM tolbutamide or diazoxide for 2 h. Insulin levels were measured using the porcine insulin radioimmunoassay (RIA) (Millipore, PI-12K). Human insulin was used for standard curves.

### Transcriptomics and qPCR

HDL-RNA was isolated from equivalent amounts of HDL total protein and then quantified by RT-PCR or sRNA sequencing. Total RNA was isolated using either Total RNA Purification Kit (Norgen Biotek) or miRNeasy Mini Kit (Qiagen). HDL-RNA was isolated from equal HDL-protein amounts for each experiment. Cell and tissue RNA was diluted to equal concentrations. miRNA reverse transcription was performed using TaqMan MicroRNA Reverse Transcription Kit and predesigned TaqMan assays for each miRNAs Mix (Applied Biosystems). mRNA cDNA was obtained using High-Capacity cDNA Reverse Transcription Kit (Applied Biosystems). qPCR was performed for 40 cycles using Taqman Universal PCR and predesigned Taqman assays for each miRNA or mRNA (Applied Biosystems). Ct values were normalized to a housekeeping gene, U6 for miRNAs and Ppia for mRNAs. Absolute quantification of miR-375 was performed by comparing Cts for samples of unknown miRNA concentration to a standard curve created with known concentration of miR-375 oligonucleotides, ranging from 3 fM–400 pM (Integrated DNA Technologies). Real-time PCR was performed on the QuantStudio 6 Real-Time PCR System, as per according to manufacturer’s instructions (Life Technologies).

### Western Blotting

Whole cell lysates were isolated using 150 mM NaCl, 1% NP-40, 0.1% SDS, 100 mM Tris-HCl, pH 7.4, supplemented with protease inhibitors (Roche) and cleared by centrifugation at 4 °C for 10 min at 10,000 × g. Proteins lysates were separated by 4–12% SDS PAGE and then transferred to nitrocellulose membranes. The membranes were probed with monoclonal rabbit anti-SR-BI (Abcam, ab180383, 1:1,000), mouse monoclonal anti-ABCA1 (Abcam, ab18180, 1:1,000), rabbit polyclonal anti-ABCG1 (Novus Biologics, NB400-132, Lot F3, 1:1,000 dilution), or monoclonal mouse anti-GAPDH (Sigma, G8795, 1:10,000) in TBS-Tween20 containing 5% non-fat dry milk. HRP-conjugated secondary antibodies we used: anti-mouse (Promega, W4028, Lot 0000214819, 1:15,000) and anti-rabbit (Promega, W4018, Lot 0000212738, 1:20,000). Immune complexes were detected with Western Lightning Plus-ECL (Perkin Elmer) or Amersham ECL Prime Western Blotting Detection Reagent (GE Healthcare Life Sciences) chemiluminescent substrate.

### Small RNA Sequencing

Total RNA from human HDL (Table SI), human islets export (Fig. 1b and Table SII), rat INS-1 cell export (Fig. 2a and Table SIV) were prepared with TruSeq small RNA library kits (Illumina) and sequenced on the HiSeq2500 sequencer SE50 (Illumina). Total RNA from human islets (Table SIII) were prepared with TruSeq sRNA kits (Illumina) and sequenced on the NextSeq500 sequencer SE75 (Illumina). All kits were performed as per manufacturer’s instruction with added amplification cycles. Prior to sequencing samples were size-selected by Pippin-Prep (Sage Science) to collect cDNA 135–200 nts in length. Libraries were cleaned and concentrated (DNA Clean and Concentrator 5 kit, Zymo), tested for quality (High-Sensitivity DNA chips, 2100 Bioanalyzer, Agilent), and quantified (High-Sensitivity DNA assays, Qubit, Life Technologies). Equal concentrations samples were pooled for multiplex sequencing and concentrated (DNA Clean and Concentrator 5 kit, Zymo).

### Informatics

miRNA sequencing data were analyzed by an in-house sRNA-seq data analysis pipeline^45^. Briefly, Cutadapt was used to remove adapters and reads >16 nts in length were aligned to the rat (rno5) or human (Hg18) genome with 1 mismatch allowance by Bowtie1 (v1.1.2)^46^ and DEseq2 (v1.18.1)^47^ was used for differential expression between cf-nHDL and INS-1-nHDL or islet-nHDL. For human HDL and islets, reads were normalized and reported as reads per million total reads. For MAplots, log_2_ fold change and log_2_ normalized counts were calculated using DESEq2 v1.20.0. Circos plot was generated with R package Circlize v0.4.3. Briefly, log_2_ fold change of miRNAs exported to HDL were calculated with DESEq2 v1.20.0 and sorted in descending order. Human islet miRNA counts were normalized by total reads per million, averaged among technical replicates, and sorted in descending abundance. Top 100 miRNAs were plotted. Links represent miRNAs exported to HDL that were also present in human islets.

### Statistics

Comparisons between two groups: Two-tailed t-tests were used. One-way ANOVA with Bonferonni post-correction alpha = 0.05 was used to compare between multiple groups. A p < 0.05 was considered significant. Scatter plots indicate replicates with mean ± 95% CI.

## Supplementary information

Supplementary Material

## Data Availability

Sequencing datasets are available through the Gene Expression Omnibus (GEO): GSE124559, GSE125135, GSE125136, GSE125137.
