# Supplementary material for: Beta cell secretion of miR-375 to HDL is inversely associated with insulin secretion
Source: Sci Rep. 2019 Mar 7;9:3803. doi: 10.1038/s41598-019-40338-7 (PMC6405899; doi:10.1038/s41598-019-40338-7)
Supplement: Supplementary file 1 — Supplementary Material [file 41598_2019_40338_MOESM1_ESM.pdf]

# Beta cell secretion of miR-375 to HDL is inversely associated with insulin secretion

Leslie R. Sedgeman, Carine Beysen, Marisol A. Ramirez Solano, Danielle L. Michell, Quanhu Sheng, Shilin Zhao, Scott Turner, MacRae F. Linton, Kasey C. Vickers

**Supplementary Information:**

Supplementary Figure 1. Quantification of HDL miR-375-3p islet exported miRNAs.

Supplementary Figure 2. Quantification of INS-1 exported miRNAs.

Supplementary Figure 3. Cellular expression of miR-375 during time course.

Supplementary Figure 4. Flow cytometry quantification of cell cycle.

Supplementary Figure 5. miR-375-3p and pri-miR-375 during cell cycle.

Supplementary Figure 6. Cellular miR-375 levels and insulin secretion with tolbutamide and diazoxide.

Supplementary Figure 7. Confirmation of *Abcc8*$^{-/-}$ genotype and cellular levels of miR-375-3p.

Supplementary Figure 8. Expression of Fos, miR-375-3p and pri-miR-375 with IBMX and exendin-4.

Supplementary Figure 9. Cellular expression of Scarb1, Abca1 and Abcg1.

Supplemental Figure 10. Full western blot images.

Supplementary Table I: Normalized miRNA rank (RPM) in human (healthy) HDL by sRNA-seq. Mean from n=10

Supplementary Table II: Primary human islet miRNA export to HDL. Fold change of islet-nHDL versus cf-nHDL from 1 human islet donor.

Supplementary Table III: Normalized miRNA rank (RPM) in primary human islets by sRNA-seq from 1 human donor.

Supplementary Table IV: INS-1 cell miRNA export to HDL. Fold change of INS-1-nHDL versus cf-nHDL from n=1 pool of 3 samples.

a

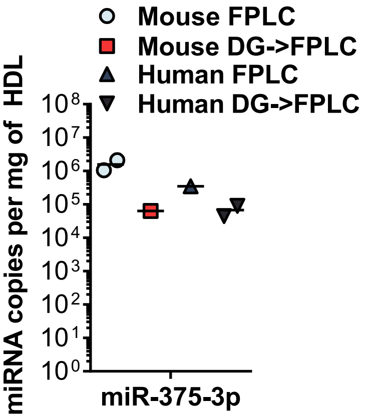


b

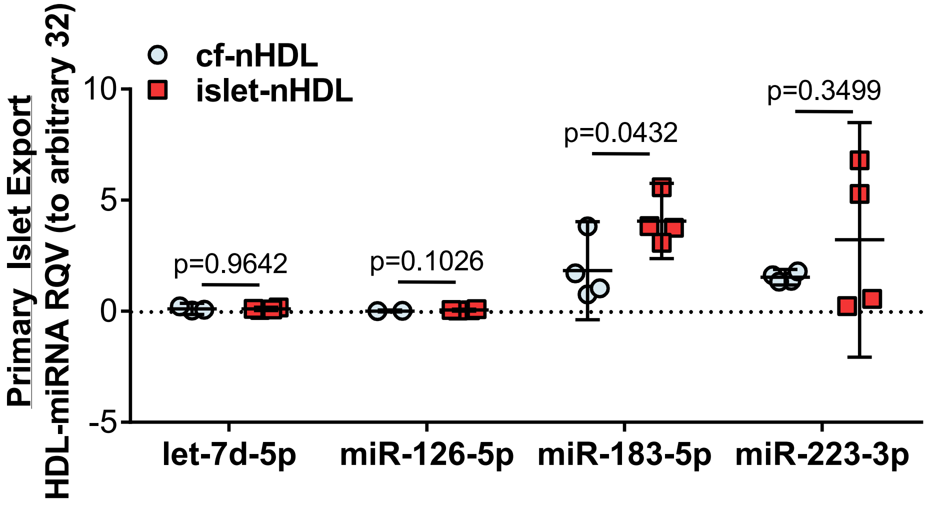


**Supplementary Figure 1. Quantification of HDL miR-375-3p and islet exported miRNAs. (a)** Absolute quantification (qPCR) of mouse and human HDL-miRNAs from 1mg of HDL isolated by fast-protein liquid chromatography (FPLC) or density-gradient ultracentrifugation (DGUC) followed by FPLC. n=1-2; Mean ± 95% CI. **(b)** let-7d-5p, miR-126-5p, miR-183-5p, miR-223-3p levels on cf-nHDL and islet-nHDL from islet preps from donor 3. n=4; mean ± 95% CI; Two-tailed t-test.

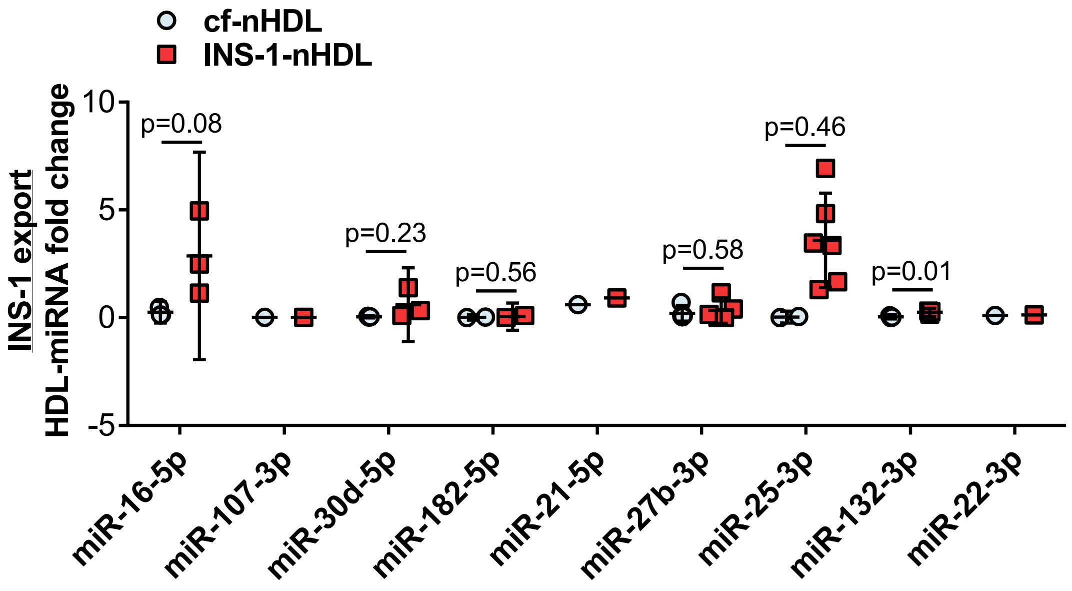


**Supplementary Figure 2. Quantification of INS-1 exported miRNAs.** miR-16-5p, miR-107-3p, miR-30d-5p, miR-182-5p, miR-21-5p, miR-27b-3p, miR-25-3p, miR-132-3p, miR-22-3p export to nHDL from INS-1 cells. n=1-6; mean ± 95% CI; Two-tailed t-test.

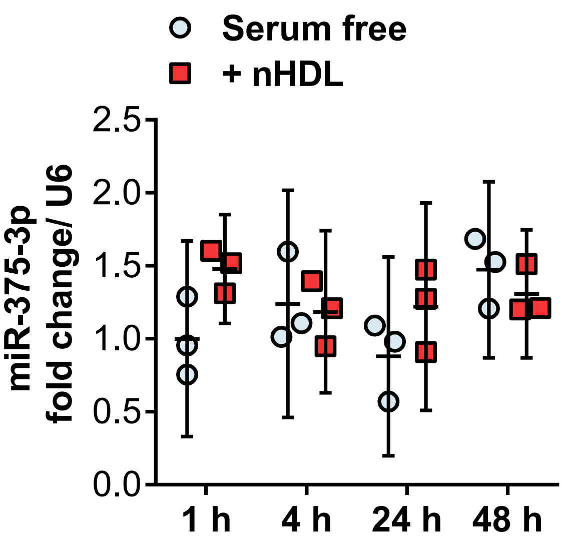


**Supplementary Figure 3. Cellular expression of miR-375 during time course. (a)** Cellular miR-375 levels in INS-1 cells treated with or without nHDL for 1, 4, 24 and 48h. n=3; mean ± 95% CI; One-way ANOVA with Bonferroni post-test, alpha = 0.05.

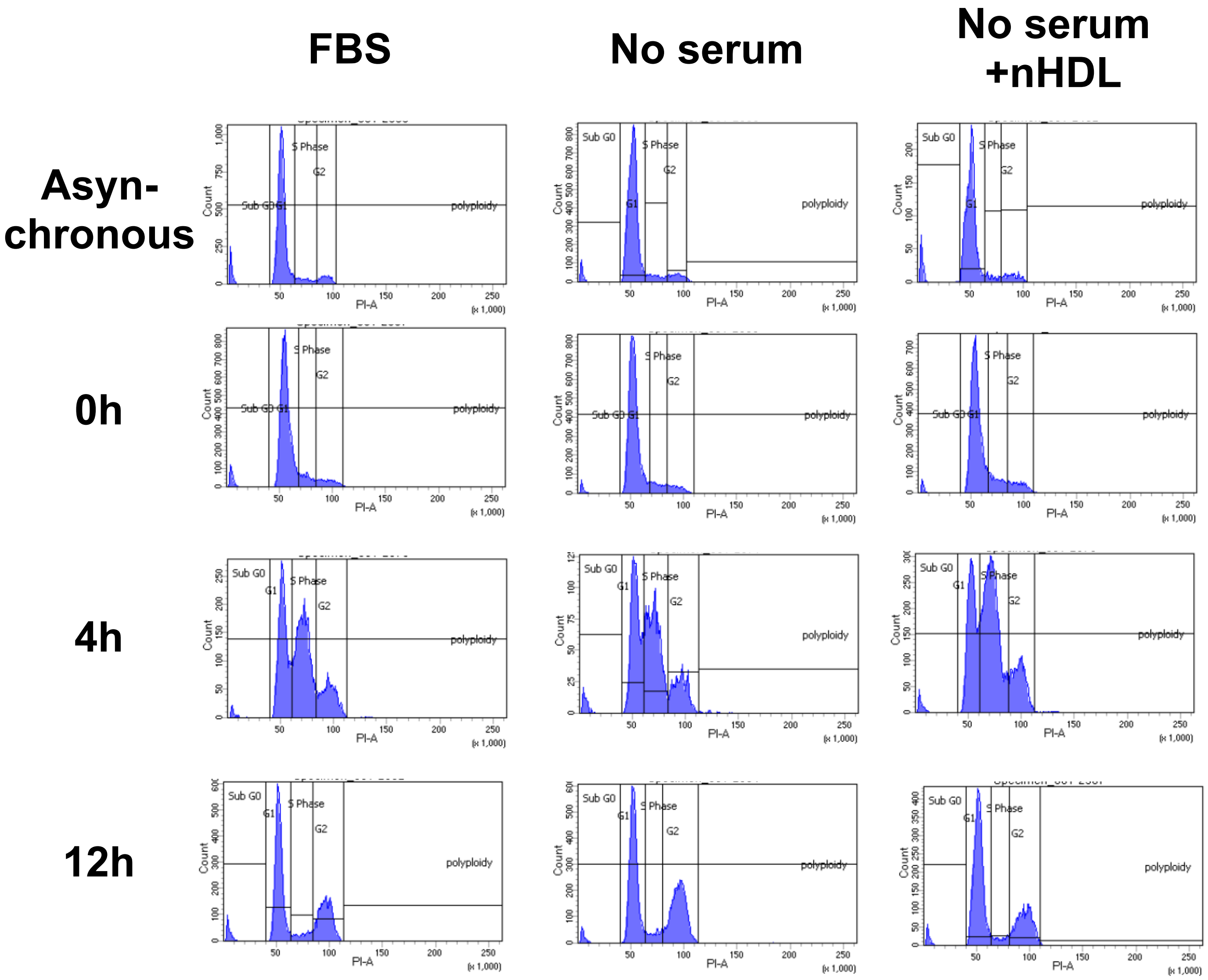


**Supplementary Figure 4.** Flow cytometry quantification of cell cycle. Representative flow cytometry plots of cells in G1, S and G2 phase in asynchronous or after 0h, 4h or 12h after G1 arrest.

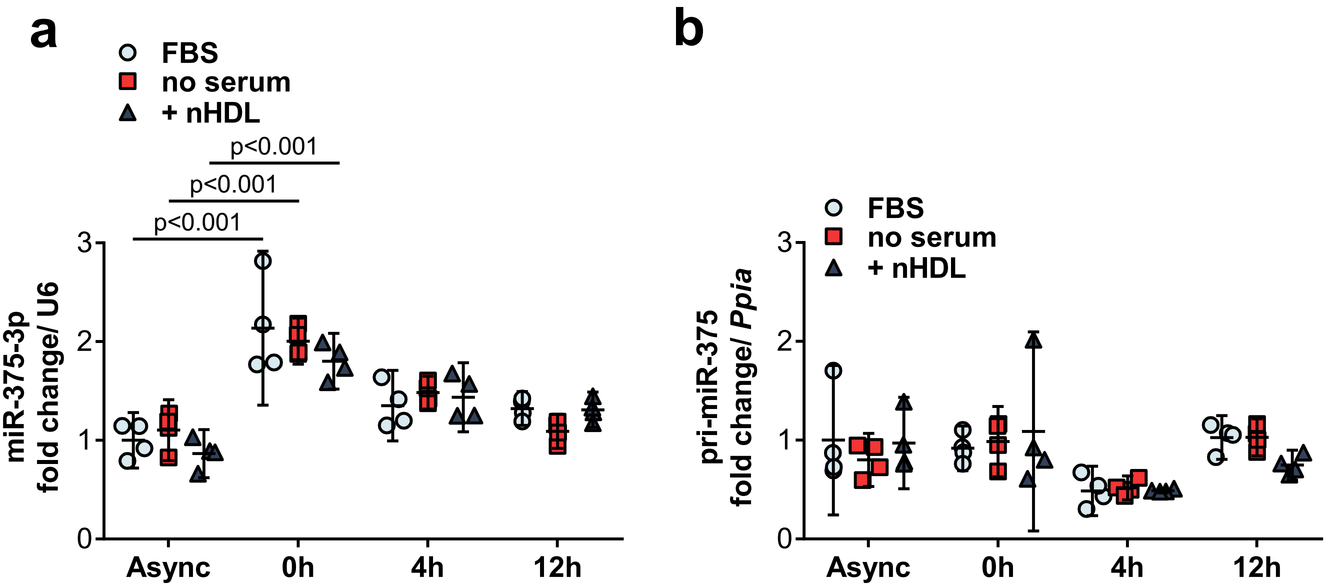


**Supplementary Figure 5. miR-375-3p and pri-miR-375 during cell cycle. (a)** Cellular miR-375 and **(b)** pri-miR-375 in asynchronous INS-1 cells or after 0, 4, or 12h after G1 arrest with or without serum and HDL. n=4; mean ± 95% CI; One-way ANOVA with Bonferroni post-test, alpha = 0.05.

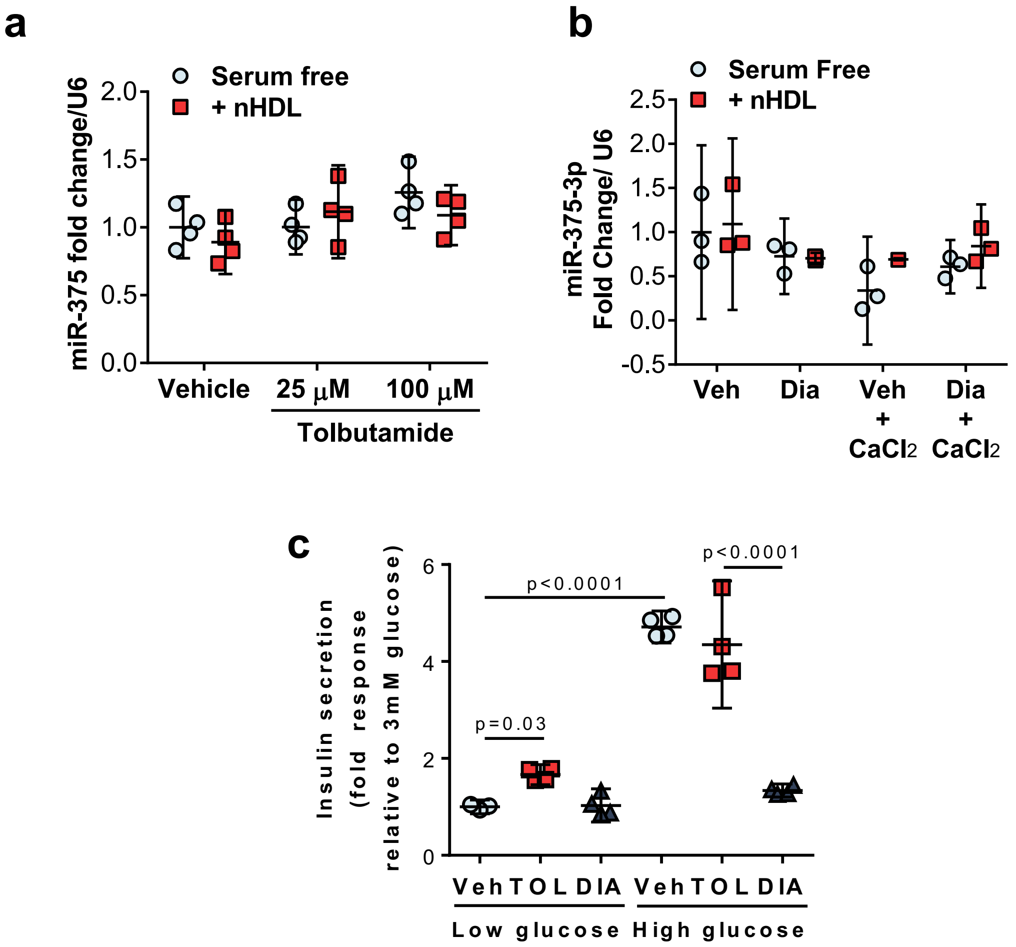


**Supplementary Figure 6. Cellular miR-375 levels and insulin secretion with tolbutamide and diazoxide. (a)** Cellular miR-375-3p levels in INS-1 cells treated with tolbutamide for 3h. n=4; mean ± 95% CI; One-way ANOVA with Bonferroni post-test, alpha = 0.05.
**(b)** Cellular levels of miR-375-3p in INS-1 cells treated diazoxide (Dia) with or without HDL for 3h in the presence or absence of $CaCl_2$. n=3; mean ± 95% CI; One-way ANOVA with Bonferroni post-test, alpha = 0.05. **(c)** Insulin secretion to media from INS-1 cells in low (3mM) or high (15mM) glucose supplemented with 200uM diazoxide (DIA) or tolbutamide (TOL). n=3-4; mean ± 95% CI; One-way ANOVA with Bonferroni post-test, alpha = 0.05.

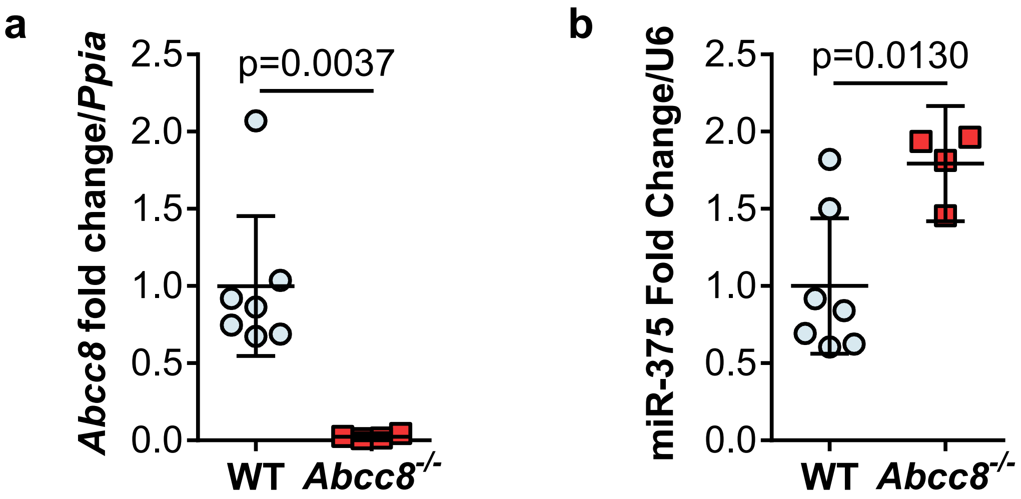


**Supplementary Figure 7. Confirmation of Abcc8$^{-/-}$ genotype and cellular levels of miR-375-3p. (a)** Expression of Abcc8 and **(b)** miR-375-3p in primary islets from WT (wildtype) or Abcc8$^{-/-}$ mice. n=3; mean ± 95% CI; two-tailed t-test.

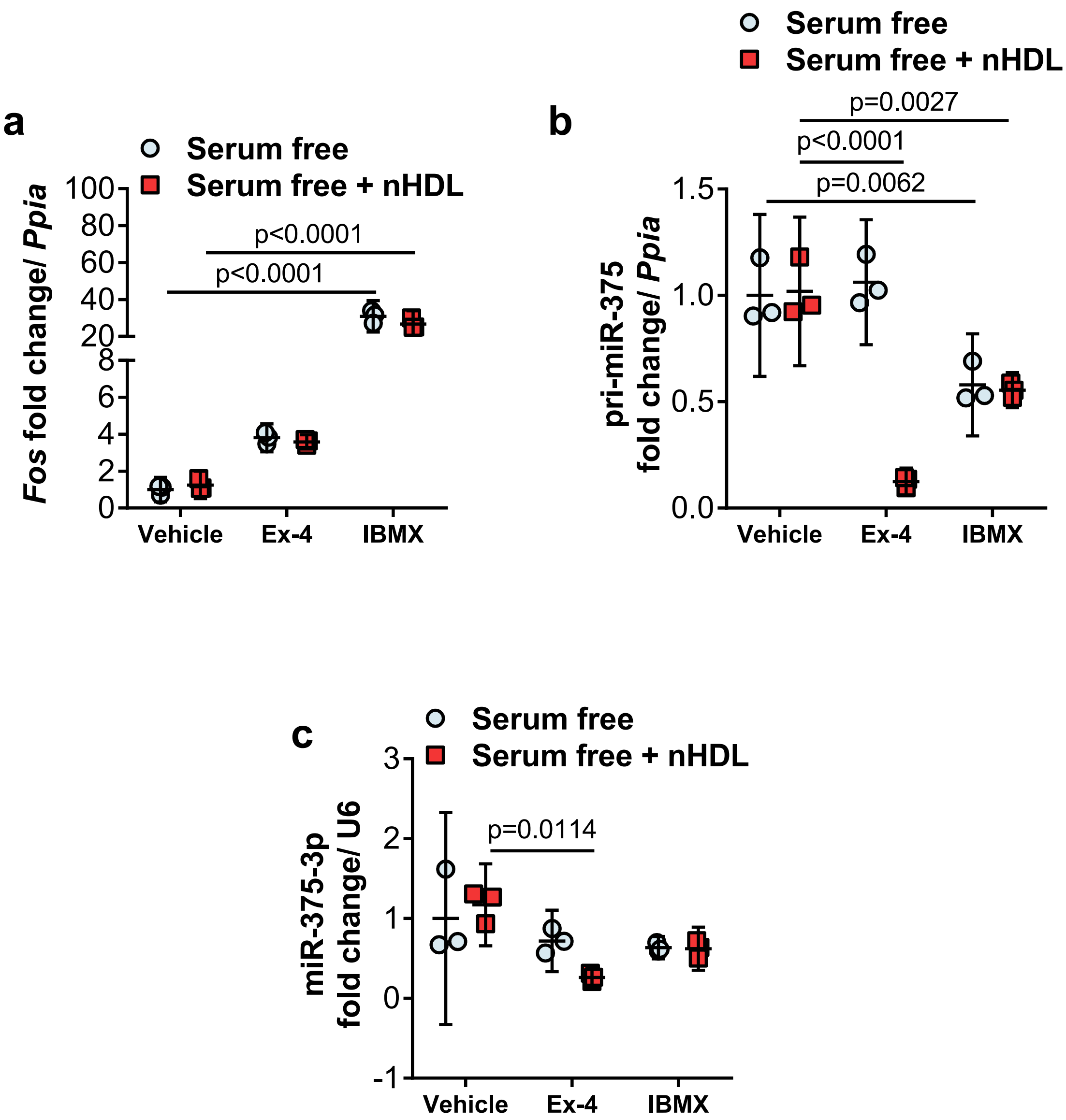


**Supplementary Figure 8. Expression of Fos, miR-375-3p and pri-miR-375 with IBMX and exendin-4.** **(a)** Cellular levels of *Fos*, **(b)** pri-miR-375 and **(c)** miR-375-3p in INS-1 cells treated with ex-4 or IBMX with or without HDL for 3h. n=3; mean ± 95% CI; One-way ANOVA with Bonferroni post-test, alpha = 0.05.

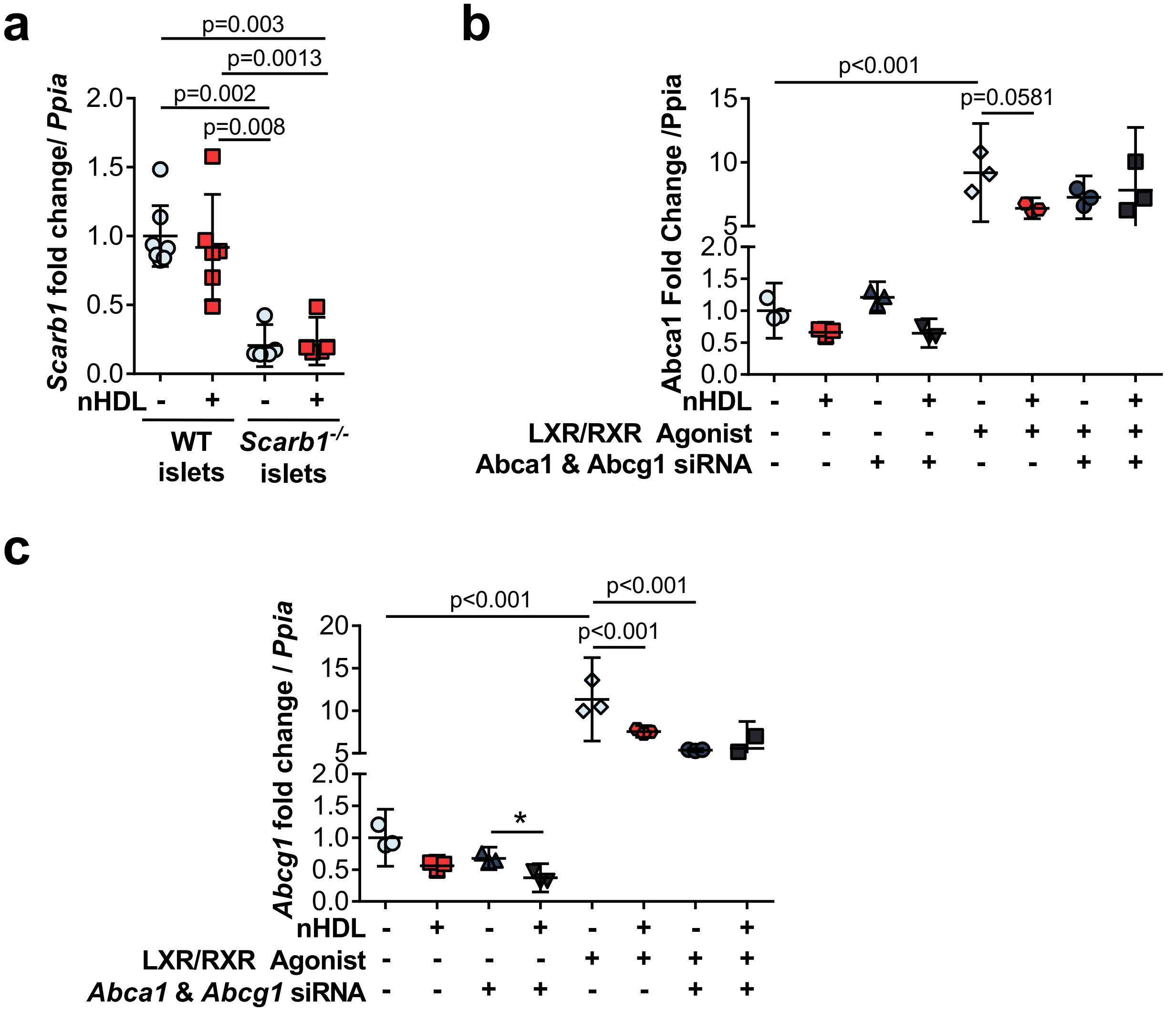


**Supplementary Figure 9. Cellular expression of Scarb1, Abca1 and Abcg1. (a)** SR-BI and wildtype islet levels of *Scarb1* mRNA. n=3; mean ± 95% CI; One-way ANOVA with Bonferroni post-test, alpha = 0.05. **(b)** Abca1 and **(c)** Abcg1 mRNA levels in INS-1 cells treated with Abca1 and Abcg1 siRNA and/or LXR and RXR agonist. n=3; mean ± 95% CI; One-way ANOVA with Bonferroni post-test, alpha = 0.05.

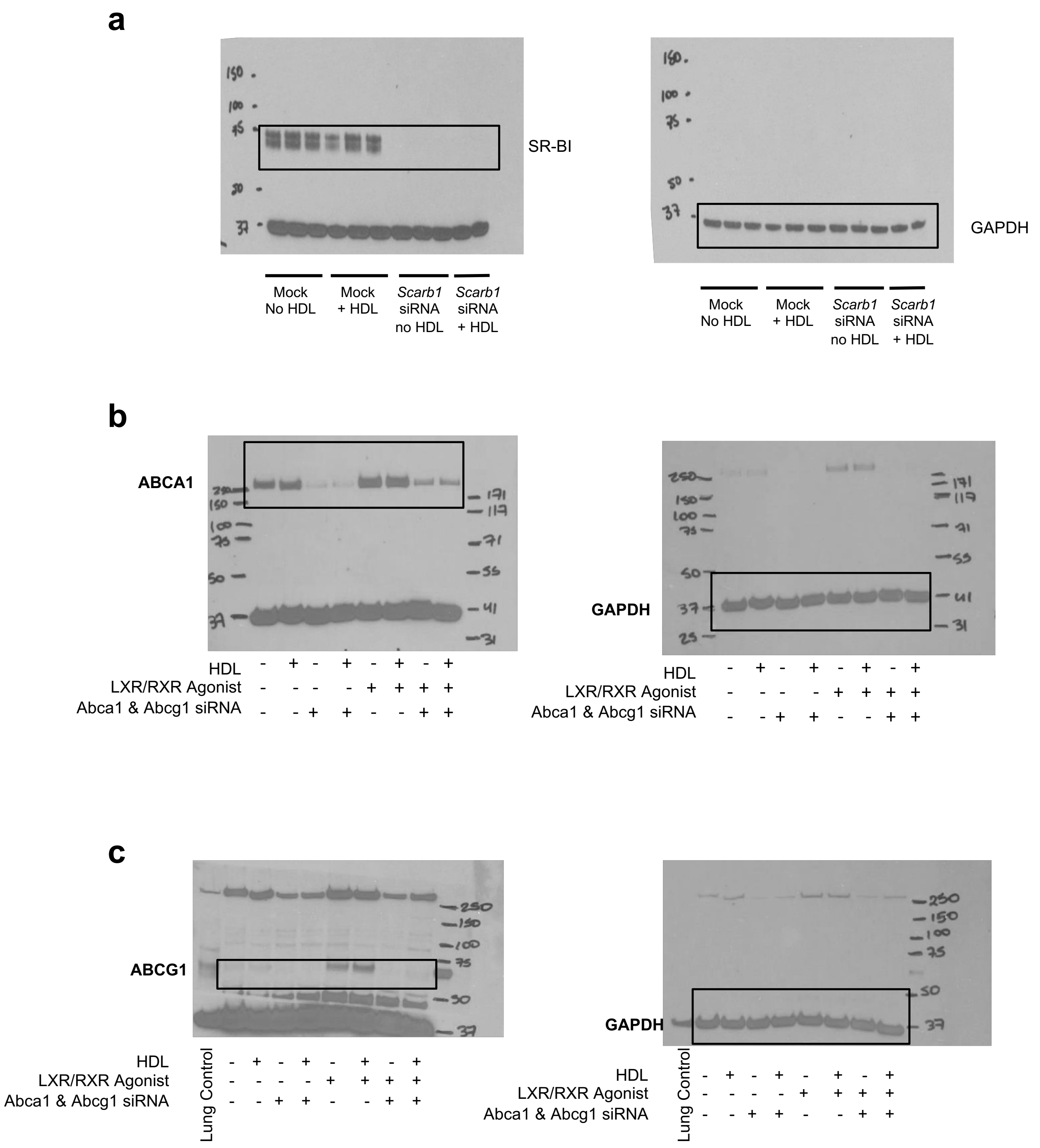


**Supplemental Figure 10. Full western blot images. (A)** Full Western blot from Fig 4C. **(B)** Full Western blot from Fig 4E. **(C)** Full Western blot from Fig 4F. Black box indicates cropped images shown in Fig 4.

**Table SI. Normalized miRNA rank (RPM) in human (healthy) HDL by sRNA-seq. Mean from n=10**

| Feature | Mean RPM | Standard Deviation |
|---|---|---|
| hsa-miR-486-5p | 788.0112 | 456.7136 |
| hsa-miR-27b-3p | 345.1239 | 178.1848 |
| hsa-miR-423-5p | 319.4812 | 281.671 |
| hsa-miR-92a-3p | 281.4443 | 244.7793 |
| hsa-miR-3168 | 195.0576 | 152.0776 |
| hsa-miR-22-3p | 173.6318 | 140.1347 |
| hsa-miR-26a-5p | 161.2463 | 162.8545 |
| hsa-miR-16-5p | 133.8386 | 74.41697 |
| hsa-miR-191-5p | 119.7854 | 105.1068 |
| hsa-let-7f-5p | 108.0859 | 116.4362 |
| hsa-miR-126-5p | 71.14003 | 55.7222 |
| hsa-miR-21-5p | 69.68608 | 88.02777 |
| hsa-miR-30d-5p | 61.40914 | 85.8951 |
| hsa-let-7i-5p | 57.19072 | 81.51391 |
| hsa-let-7a-5p | 52.71567 | 62.44602 |
| hsa-miR-181a-5p | 51.00064 | 59.93619 |
| hsa-miR-30e-5p | 46.35006 | 58.70766 |
| hsa-miR-375 | 44.40288 | 17.06922 |
| hsa-miR-320a | 41.24771 | 48.00825 |
| hsa-miR-146a-5p | 39.45484 | 47.21153 |
| hsa-miR-203a | 34.63446 | 10.18178 |
| hsa-miR-320b | 29.35724 | 18.41114 |
| hsa-miR-151a-3p | 27.6612 | 25.07574 |
| hsa-miR-451a | 26.84648 | 18.20303 |
| hsa-miR-25-3p | 19.52505 | 11.03535 |
| hsa-let-7g-5p | 17.10731 | 18.65177 |
| hsa-miR-127-3p | 16.93047 | 34.60336 |
| hsa-miR-182-5p | 16.74405 | 12.31013 |
| hsa-miR-26b-5p | 14.79317 | 15.6524 |
| hsa-miR-146b-5p | 14.56165 | 17.50995 |
| hsa-miR-222-3p | 14.31521 | 19.17382 |
| hsa-miR-130a-3p | 13.66696 | 7.699563 |
| hsa-miR-423-3p | 13.18309 | 14.12278 |
| hsa-miR-28-3p | 12.58219 | 15.78282 |
| hsa-miR-10a-5p | 12.55418 | 10.21123 |
| hsa-miR-10b-5p | 12.48325 | 9.664026 |
| hsa-miR-143-3p | 12.41597 | 9.269474 |
| hsa-miR-584-5p | 12.39155 | 19.99658 |
| hsa-miR-103a-3p | 12.32826 | 14.61766 |

| | | |
|---|---|---|
| hsa-miR-151a-5p | 11.81064 | 16.00837 |
| hsa-miR-148a-3p | 11.57163 | 9.780746 |
| hsa-miR-3621 | 11.4806 | 5.934623 |
| hsa-miR-186-5p | 10.89291 | 15.13651 |
| hsa-miR-148a-5p | 10.56691 | 12.71385 |
| hsa-miR-15a-5p | 10.44135 | 9.346207 |
| hsa-miR-192-5p | 9.9571 | 7.213854 |
| hsa-miR-199a-3p;hsa-miR-199b-3p | 9.835791 | 12.65265 |
| hsa-let-7b-5p | 9.015215 | 12.82158 |
| hsa-miR-126-3p | 8.308532 | 9.860342 |
| hsa-miR-27a-3p | 7.972251 | 3.4671 |
| hsa-miR-92b-3p | 7.087933 | 4.789983 |
| hsa-let-7d-5p | 7.001503 | 11.70697 |
| hsa-miR-125a-3p | 6.29544 | 6.96409 |
| hsa-miR-744-5p | 5.786959 | 7.174524 |
| hsa-miR-181b-5p | 5.745241 | 7.154621 |
| hsa-miR-148b-3p | 5.511017 | 9.116341 |
| hsa-miR-30c-5p | 5.040731 | 7.490189 |
| hsa-miR-140-3p | 4.971965 | 6.836437 |
| hsa-miR-30a-5p | 4.825282 | 4.719443 |
| hsa-miR-93-5p | 4.43947 | 5.802726 |
| hsa-let-7e-5p | 4.387744 | 4.778715 |
| hsa-miR-877-5p | 4.125504 | 9.271787 |
| hsa-miR-409-3p | 3.578761 | 4.523906 |
| hsa-miR-378a-3p | 3.436597 | 2.949803 |
| hsa-miR-101-3p | 3.249335 | 3.962054 |
| hsa-miR-432-5p | 3.210909 | 7.056805 |
| hsa-miR-17-5p | 3.156199 | 3.161019 |
| hsa-miR-23a-3p | 3.101793 | 2.11827 |
| hsa-miR-142-5p | 3.085366 | 4.266771 |
| hsa-miR-223-3p | 2.996284 | 5.238771 |
| hsa-miR-1246 | 2.757549 | 2.71853 |
| hsa-miR-301a-3p | 2.698945 | 2.51427 |
| hsa-miR-184 | 2.426209 | 3.493294 |
| hsa-miR-484 | 2.417394 | 5.216184 |
| hsa-miR-98-5p | 2.375291 | 5.016895 |
| hsa-miR-142-3p | 2.276818 | 3.146609 |
| hsa-miR-221-3p | 2.251317 | 4.004715 |
| hsa-miR-130b-3p | 2.124789 | 2.825755 |
| hsa-miR-425-5p | 1.933646 | 5.026174 |
| hsa-miR-23b-3p | 1.885075 | 1.789049 |
| hsa-miR-155-5p | 1.80416 | 2.732211 |
| hsa-miR-100-5p | 1.758465 | 5.560754 |
| hsa-miR-107 | 1.745213 | 0.718582 |
| hsa-miR-410-3p | 1.659829 | 4.051417 |

| | | |
|---|---|---|
| hsa-miR-486-3p | 1.639299 | 2.703247 |
| hsa-miR-20a-5p | 1.620598 | 2.73591 |
| hsa-miR-128-3p | 1.514984 | 2.654882 |
| hsa-miR-421 | 1.480628 | 3.214629 |
| hsa-miR-30e-3p | 1.374828 | 2.630024 |
| hsa-miR-28-5p | 1.363409 | 2.404024 |
| hsa-miR-221-5p | 1.284207 | 2.719184 |
| hsa-miR-340-5p | 1.230565 | 2.626052 |
| hsa-miR-19a-3p | 1.204221 | 2.756621 |
| hsa-miR-15b-5p | 1.202127 | 2.605209 |
| hsa-miR-144-3p | 1.192036 | 1.546762 |
| hsa-miR-433-3p | 1.156504 | 2.439425 |
| hsa-miR-7977 | 1.14226 | 1.158401 |
| hsa-miR-133a-3p | 1.089457 | 1.775504 |
| hsa-miR-654-3p | 1.057014 | 1.921373 |
| hsa-miR-224-5p | 0.994556 | 2.293266 |
| hsa-miR-136-3p | 0.936677 | 2.081764 |
| hsa-miR-323b-3p | 0.888826 | 2.810715 |
| hsa-miR-627-5p | 0.861207 | 1.825931 |
| hsa-miR-342-3p | 0.847269 | 2.679301 |
| hsa-miR-766-5p | 0.842046 | 2.662783 |
| hsa-miR-93-3p | 0.837221 | 1.822078 |
| hsa-miR-181c-3p | 0.83713 | 1.777149 |
| hsa-miR-885-3p | 0.810446 | 1.710193 |
| hsa-miR-125a-5p | 0.795265 | 2.51485 |
| hsa-miR-361-3p | 0.795265 | 2.51485 |
| hsa-miR-629-5p | 0.793521 | 2.509333 |
| hsa-miR-4732-3p | 0.778847 | 2.462929 |
| hsa-miR-1307-3p | 0.770245 | 2.182074 |
| hsa-miR-194-5p | 0.767475 | 1.634957 |
| hsa-miR-4516 | 0.767334 | 1.369029 |
| hsa-miR-185-5p | 0.760457 | 2.404777 |
| hsa-miR-21-3p | 0.751251 | 1.248465 |
| hsa-miR-3615 | 0.734768 | 2.209812 |
| hsa-miR-891a-5p | 0.734547 | 2.322842 |
| hsa-miR-4520a-3p | 0.72119 | 2.280602 |
| hsa-miR-106b-5p | 0.717454 | 1.439748 |
| hsa-miR-335-3p | 0.702211 | 2.220586 |
| hsa-miR-889-3p | 0.701705 | 2.218985 |
| hsa-let-7c-5p | 0.683756 | 1.211577 |
| hsa-miR-103b | 0.681772 | 1.566444 |
| hsa-miR-30b-5p | 0.672703 | 1.770978 |
| hsa-miR-215-5p | 0.666892 | 1.225463 |
| hsa-miR-106b-3p | 0.637828 | 1.144105 |
| hsa-miR-652-3p | 0.628433 | 1.376372 |

| | | |
|---|---|---|
| hsa-miR-146b-3p | 0.628204 | 1.986555 |
| hsa-miR-374b-5p | 0.612364 | 1.936466 |
| hsa-miR-181c-5p | 0.608144 | 1.923121 |
| hsa-miR-5010-3p | 0.608144 | 1.923121 |
| hsa-miR-1304-3p | 0.600451 | 1.898794 |
| hsa-miR-151b | 0.592301 | 0.634469 |
| hsa-miR-99b-5p | 0.584135 | 1.847197 |
| hsa-miR-6852-5p | 0.581105 | 1.212846 |
| hsa-miR-181a-2-3p | 0.579217 | 1.369252 |
| hsa-miR-744-3p | 0.56936 | 1.800475 |
| hsa-miR-425-3p | 0.561364 | 1.775188 |
| hsa-miR-500a-3p | 0.561364 | 1.775188 |
| hsa-miR-3688-3p | 0.557562 | 1.763166 |
| hsa-miR-4446-3p | 0.538702 | 1.185676 |
| hsa-miR-411-5p | 0.502102 | 0.817138 |
| hsa-miR-4532 | 0.500773 | 0.75705 |
| hsa-miR-6748-3p | 0.476222 | 0.88888 |
| hsa-let-7d-3p | 0.474467 | 1.500396 |
| hsa-miR-197-5p | 0.474467 | 1.500396 |
| hsa-miR-660-5p | 0.472779 | 1.435334 |
| hsa-miR-199b-5p | 0.448907 | 1.247594 |
| hsa-miR-548n | 0.445356 | 1.408339 |
| hsa-miR-671-3p | 0.433076 | 0.994592 |
| hsa-miR-625-5p | 0.422487 | 1.224265 |
| hsa-miR-485-5p | 0.421023 | 1.331391 |
| hsa-miR-1343-3p | 0.408534 | 0.334219 |
| hsa-miR-150-5p | 0.389423 | 1.231464 |
| hsa-miR-200a-3p | 0.389423 | 1.231464 |
| hsa-miR-125b-5p | 0.386005 | 1.220653 |
| hsa-miR-26b-3p | 0.386005 | 1.220653 |
| hsa-miR-363-3p | 0.374924 | 0.823524 |
| hsa-miR-4485 | 0.363697 | 1.150111 |
| hsa-miR-4757-3p | 0.354887 | 0.991115 |
| hsa-miR-5189-3p | 0.354755 | 1.121835 |
| hsa-miR-4772-3p | 0.349526 | 0.737021 |
| hsa-miR-183-5p | 0.346803 | 0.49908 |
| hsa-miR-301b | 0.341616 | 1.080285 |
| hsa-miR-19b-3p | 0.336163 | 1.063041 |
| hsa-miR-4433b-3p | 0.330634 | 1.045555 |
| hsa-miR-370-3p | 0.327462 | 1.035527 |
| hsa-miR-378i | 0.327462 | 1.035527 |
| hsa-miR-505-3p | 0.327462 | 1.035527 |
| hsa-miR-7975 | 0.325754 | 0.705431 |
| hsa-miR-381-3p | 0.280682 | 0.887594 |
| hsa-miR-4742-3p | 0.280682 | 0.887594 |

| | | |
|---|---|---|
| hsa-miR-5010-5p | 0.280682 | 0.887594 |
| hsa-miR-483-3p | 0.275921 | 0.872538 |
| hsa-miR-187-5p | 0.264507 | 0.836444 |
| hsa-miR-496 | 0.257336 | 0.813769 |
| hsa-miR-760 | 0.233902 | 0.739662 |
| hsa-miR-17-3p | 0.23372 | 0.739086 |
| hsa-miR-141-3p | 0.214447 | 0.678141 |
| hsa-miR-6791-3p | 0.194843 | 0.616148 |
| hsa-miR-369-3p | 0.187121 | 0.591729 |
| hsa-miR-431-5p | 0.187121 | 0.591729 |
| hsa-miR-769-5p | 0.187121 | 0.591729 |
| hsa-miR-6869-5p | 0.170524 | 0.369907 |
| hsa-miR-320d | 0.169824 | 0.248057 |
| hsa-miR-24-3p | 0.161693 | 0.341672 |
| hsa-miR-1260b | 0.140341 | 0.443797 |
| hsa-miR-6087 | 0.119845 | 0.281921 |
| hsa-miR-4530 | 0.118646 | 0.375191 |
| hsa-miR-384 | 0.118252 | 0.373945 |
| hsa-miR-7704 | 0.093561 | 0.295865 |
| hsa-miR-3191-3p | 0.083504 | 0.264064 |
| hsa-miR-6791-5p | 0.078835 | 0.249297 |
| hsa-miR-511-3p | 0.075953 | 0.161789 |
| hsa-miR-320c | 0.073291 | 0.083652 |
| hsa-miR-4508 | 0.069165 | 0.218718 |
| hsa-miR-497-5p | 0.059323 | 0.187596 |
| hsa-miR-3610 | 0.056936 | 0.180048 |
| hsa-miR-4492 | 0.055669 | 0.176042 |
| hsa-miR-323a-3p | 0.04678 | 0.147932 |
| hsa-miR-6073 | 0.04678 | 0.147932 |
| hsa-miR-205-5p | 0.039417 | 0.124648 |
| hsa-miR-3150b-5p | 0.039417 | 0.124648 |
| hsa-miR-4783-3p | 0.039417 | 0.124648 |
| hsa-miR-193a-3p | 0.033389 | 0.105584 |
| hsa-miR-4662b | 0.033063 | 0.104556 |
| hsa-miR-3074-3p | 0.027835 | 0.088021 |
| hsa-miR-1247-3p | 0.019774 | 0.062532 |
| hsa-miR-3150b-3p | 0.019774 | 0.062532 |
| hsa-miR-3184-3p | 0.019774 | 0.062532 |
| hsa-miR-6516-5p | 0.018979 | 0.060016 |
| hsa-miR-6851-5p | 0.018979 | 0.060016 |
| hsa-miR-7641 | 0.018979 | 0.060016 |
| hsa-miR-145-3p | 0.017291 | 0.05468 |
| hsa-miR-3622b-3p | 0.017291 | 0.05468 |
| hsa-miR-6834-5p | 0.017291 | 0.05468 |

**Table SII. Primary human islet miRNA export to HDL. Fold change of islet-nHDL versus cf-nHDL from 1 human islet donor.**

| miRNA | DEseq2 (BaseMean) | Fold Change | cf-nHDL (RPM) | Islet-nHDL (RPM) |
|---|---|---|---|---|
| hsa-miR-375 | 3937.38 | 16.77 | 160.00 | 3944.76 |
| hsa-let-7d-5p | 117.55 | 2.34 | 50.02 | 94.32 |
| hsa-miR-200a-3p | 37.91 | 2.03 | 17.32 | 35.74 |
| hsa-miR-148a-3p | 285.12 | 2.02 | 145.47 | 203.56 |
| hsa-miR-30c-5p | 205.87 | 2.02 | 118.93 | 160.70 |
| hsa-miR-30a-5p | 812.26 | 1.85 | 441.25 | 606.52 |
| hsa-miR-223-3p | 38.69 | 1.81 | 18.28 | 33.09 |
| hsa-miR-101-3p | 42.64 | 1.78 | 9.03 | 32.21 |
| hsa-let-7d-3p | 40.84 | 1.71 | 23.93 | 33.41 |
| hsa-miR-125a-5p | 78.79 | 1.68 | 31.70 | 63.91 |
| hsa-miR-92b-3p | 38.20 | 1.65 | 15.13 | 36.73 |
| hsa-let-7f-5p | 370.57 | 1.65 | 216.53 | 262.95 |
| hsa-miR-126-5p | 127.87 | 1.61 | 54.55 | 87.93 |
| hsa-miR-425-5p | 39.13 | 1.60 | 21.07 | 30.59 |
| hsa-miR-181b-5p | 50.86 | 1.56 | 30.08 | 36.89 |
| hsa-miR-183-5p | 10.27 | 1.55 | 4.12 | 8.81 |
| hsa-miR-30e-5p | 583.44 | 1.53 | 427.29 | 440.71 |
| hsa-miR-30d-5p | 390.14 | 1.45 | 248.94 | 285.87 |
| hsa-miR-191-5p | 314.15 | 1.41 | 181.99 | 234.27 |
| hsa-miR-127-3p | 97.64 | 1.36 | 61.42 | 59.74 |
| hsa-let-7b-5p | 51.90 | 1.32 | 36.03 | 38.72 |
| hsa-miR-205-5p | 134.97 | 1.30 | 86.85 | 94.08 |
| hsa-let-7a-5p | 156.81 | 1.29 | 108.85 | 94.95 |
| hsa-miR-93-5p | 130.65 | 1.28 | 89.93 | 84.75 |
| hsa-let-7i-5p | 555.92 | 1.27 | 369.73 | 334.91 |
| hsa-miR-215-5p | 109.33 | 1.26 | 79.99 | 67.74 |
| hsa-miR-423-5p | 303.25 | 1.25 | 211.53 | 178.56 |
| hsa-miR-194-5p | 5.45 | 1.22 | 3.88 | 5.83 |
| hsa-miR-186-5p | 83.96 | 1.20 | 51.49 | 49.76 |
| hsa-miR-145-5p | 27.27 | 1.20 | 19.36 | 18.09 |
| hsa-miR-128-3p | 63.12 | 1.17 | 51.30 | 41.06 |
| hsa-miR-451a | 462.89 | 1.10 | 324.83 | 282.24 |
| hsa-miR-92a-3p | 1649.47 | 1.10 | 1190.28 | 983.81 |
| hsa-miR-30b-5p | 114.52 | 1.10 | 94.52 | 65.59 |
| hsa-miR-200b-3p | 119.81 | 1.09 | 72.27 | 75.69 |
| hsa-miR-143-3p | 260.78 | 1.09 | 169.79 | 152.12 |
| hsa-miR-146a-5p | 56.07 | 1.09 | 39.36 | 38.21 |
| hsa-miR-574-3p | 19.10 | 1.07 | 16.58 | 13.22 |
| hsa-let-7g-5p | 137.53 | 1.07 | 51.22 | 71.36 |

| | | | | |
|---|---|---|---|---|
| hsa-miR-486-5p | 12316.90 | 1.07 | 9388.91 | 7232.77 |
| hsa-miR-22-3p | 4882.67 | 1.06 | 3506.36 | 2930.14 |
| hsa-miR-199a-3p;hsa-miR-199b-3p | 55.42 | 1.04 | 45.44 | 36.90 |
| hsa-miR-182-5p | 65.81 | 1.04 | 59.00 | 39.72 |
| hsa-miR-192-5p | 689.27 | 1.02 | 463.31 | 445.19 |
| hsa-miR-378c | 10.62 | 1.01 | 9.62 | 6.80 |
| hsa-miR-27b-3p | 594.52 | 1.00 | 432.58 | 355.21 |
| hsa-miR-144-5p | 31.70 | 1.00 | 32.20 | 13.40 |
| hsa-let-7e-5p | 31.25 | 0.99 | 12.23 | 11.40 |
| hsa-miR-532-5p | 58.47 | 0.98 | 19.76 | 28.44 |
| hsa-miR-10b-5p | 583.80 | 0.96 | 450.35 | 353.54 |
| hsa-miR-15a-5p | 128.49 | 0.96 | 124.88 | 74.53 |
| hsa-miR-138-5p | 19.92 | 0.96 | 7.75 | 10.60 |
| hsa-miR-125b-5p | 22.18 | 0.95 | 23.09 | 15.80 |
| hsa-let-7c-5p | 201.63 | 0.93 | 190.98 | 134.83 |
| hsa-miR-187-3p | 33.46 | 0.93 | 18.61 | 16.62 |
| hsa-miR-181d-5p | 11.77 | 0.92 | 12.48 | 3.68 |
| hsa-miR-378a-3p | 333.65 | 0.92 | 249.31 | 200.30 |
| hsa-miR-149-5p | 26.93 | 0.92 | 32.41 | 14.02 |
| hsa-miR-26a-5p | 352.49 | 0.91 | 257.85 | 189.49 |
| hsa-miR-100-5p | 7.19 | 0.91 | 8.65 | 2.99 |
| hsa-miR-132-3p | 19.11 | 0.90 | 13.05 | 11.64 |
| hsa-miR-150-5p | 641.35 | 0.90 | 489.70 | 361.33 |
| hsa-miR-328-3p | 66.67 | 0.88 | 67.60 | 39.32 |
| hsa-miR-29a-3p | 215.21 | 0.87 | 165.77 | 119.96 |
| hsa-miR-410-3p | 27.71 | 0.87 | 12.46 | 7.30 |
| hsa-miR-181c-5p | 57.91 | 0.86 | 46.33 | 29.33 |
| hsa-miR-107 | 34.19 | 0.86 | 29.08 | 19.38 |
| hsa-miR-25-3p | 599.40 | 0.85 | 569.82 | 313.53 |
| hsa-miR-151a-3p | 114.08 | 0.85 | 71.05 | 59.18 |
| hsa-miR-152-3p | 7.55 | 0.84 | 6.82 | 3.49 |
| hsa-miR-133a-3p | 641.86 | 0.82 | 464.52 | 338.32 |
| hsa-miR-99b-5p | 35.57 | 0.80 | 29.10 | 21.57 |
| hsa-miR-181a-5p | 725.44 | 0.77 | 579.05 | 380.52 |
| hsa-miR-106b-5p | 12.24 | 0.77 | 15.44 | 3.84 |
| hsa-miR-21-5p | 813.50 | 0.77 | 576.76 | 382.25 |
| hsa-miR-744-5p | 64.55 | 0.77 | 32.73 | 31.40 |
| hsa-miR-484 | 80.88 | 0.77 | 87.46 | 39.27 |
| hsa-miR-142-5p | 201.37 | 0.76 | 191.56 | 95.54 |
| hsa-miR-320a | 112.84 | 0.76 | 92.99 | 47.59 |
| hsa-miR-130a-3p | 81.47 | 0.74 | 72.69 | 38.11 |
| hsa-miR-140-3p | 233.31 | 0.71 | 190.94 | 113.81 |
| hsa-miR-423-3p | 111.14 | 0.71 | 90.71 | 54.98 |
| hsa-miR-27a-3p | 180.26 | 0.70 | 139.38 | 75.33 |

| | | | | |
|---|---|---|---|---|
| hsa-miR-342-3p | 68.78 | 0.68 | 43.67 | 30.53 |
| hsa-miR-1226-3p | 27.74 | 0.67 | 19.88 | 11.69 |
| hsa-miR-103a-3p | 104.45 | 0.67 | 92.26 | 50.12 |
| hsa-miR-23b-3p | 35.02 | 0.63 | 30.80 | 14.81 |
| hsa-miR-3168 | 1616.76 | 0.63 | 1191.11 | 676.32 |
| hsa-miR-23a-3p | 74.50 | 0.60 | 58.51 | 29.73 |
| hsa-miR-320b | 145.45 | 0.58 | 122.96 | 40.92 |
| hsa-miR-106b-3p | 22.94 | 0.57 | 28.78 | 6.25 |
| hsa-miR-221-3p | 64.08 | 0.54 | 60.90 | 24.23 |
| hsa-miR-320d | 6.97 | 0.53 | 12.39 | 1.31 |
| hsa-miR-125b-2-3p | 53.56 | 0.53 | 40.30 | 17.59 |
| hsa-miR-381-3p | 10.39 | 0.52 | 17.23 | 1.93 |
| hsa-miR-10a-5p | 1016.35 | 0.52 | 861.64 | 373.73 |
| hsa-miR-16-5p | 3630.80 | 0.51 | 2930.93 | 1313.72 |
| hsa-miR-222-3p | 29.47 | 0.49 | 34.51 | 3.03 |
| hsa-miR-26b-5p | 39.96 | 0.47 | 38.46 | 9.96 |
| hsa-miR-126-3p | 63.91 | 0.44 | 70.26 | 20.19 |
| hsa-miR-141-3p | 182.00 | 0.41 | 178.44 | 50.97 |
| hsa-miR-203a-3p | 387.08 | 0.38 | 372.92 | 91.65 |
| hsa-miR-210-3p | 18.49 | 0.36 | 14.90 | 1.91 |

**Table SIII. Normalized miRNA rank (RPMM) in primary human islets by sRNA-seq from 1 human donor.**

| Rank | Feature | Mean |
|---|---|---|
| 1 | hsa-miR-375 | 115165.4 |
| 2 | hsa-miR-26a-5p | 67691.51 |
| 3 | hsa-miR-22-3p | 39888.83 |
| 4 | hsa-miR-27b-3p | 33335.49 |
| 5 | hsa-let-7a-5p | 24821.51 |
| 6 | hsa-let-7f-5p | 18039.81 |
| 7 | hsa-miR-125a-5p | 18030.24 |
| 8 | hsa-miR-141-3p | 12378.82 |
| 9 | hsa-miR-16-5p | 11041.81 |
| 10 | hsa-miR-151a-5p | 10598.18 |
| 11 | hsa-miR-192-5p | 10378.69 |
| 12 | hsa-miR-148a-3p | 8673.186 |
| 13 | hsa-miR-191-5p | 8299.377 |
| 14 | hsa-miR-21-5p | 8141.678 |
| 15 | hsa-miR-143-3p | 7441.399 |
| 16 | hsa-miR-182-5p | 7029.85 |
| 17 | hsa-miR-125b-5p | 6649.971 |

| | | |
|---|---|---|
| 18 | hsa-miR-30d-5p | 5892.425 |
| 19 | hsa-let-7b-5p | 5528.996 |
| 20 | hsa-miR-29a-3p | 5139.805 |
| 21 | hsa-miR-99b-5p | 4652.784 |
| 22 | hsa-miR-181a-5p | 4641.751 |
| 23 | hsa-miR-200b-3p | 4439.209 |
| 24 | hsa-let-7g-5p | 3876.618 |
| 25 | hsa-miR-10a-5p | 3677.62 |
| 26 | hsa-let-7e-5p | 3283.382 |
| 27 | hsa-miR-126-5p | 3254.887 |
| 28 | hsa-let-7i-5p | 3251.6 |
| 29 | hsa-miR-98-5p | 3056.06 |
| 30 | hsa-miR-30b-5p | 3041.764 |
| 31 | hsa-miR-183-5p | 3040.656 |
| 32 | hsa-miR-30c-5p | 2953.583 |
| 33 | hsa-miR-423-5p | 2835.993 |
| 34 | hsa-miR-29c-3p | 2696.512 |
| 35 | hsa-miR-23b-3p | 2683.83 |
| 36 | hsa-miR-92a-3p | 2681.71 |
| 37 | hsa-miR-186-5p | 2419.887 |
| 38 | hsa-miR-26b-5p | 2398.655 |
| 39 | hsa-miR-200a-3p | 2089.876 |
| 40 | hsa-miR-222-3p | 2087.633 |
| 41 | hsa-miR-27a-3p | 2083.216 |
| 42 | hsa-miR-30a-5p | 1980.771 |
| 43 | hsa-miR-92b-3p | 1935.638 |
| 44 | hsa-miR-30e-5p | 1751.66 |
| 45 | hsa-miR-335-5p | 1627.754 |
| 46 | hsa-let-7d-5p | 1619.637 |
| 47 | hsa-miR-153-3p | 1538.779 |
| 48 | hsa-miR-423-3p | 1503.004 |
| 49 | hsa-miR-151a-3p | 1403.599 |
| 50 | hsa-miR-200c-3p | 1339.256 |
| 51 | hsa-miR-204-5p | 1277.235 |
| 52 | hsa-miR-127-3p | 1173.747 |
| 53 | hsa-miR-487b-3p | 1135.028 |
| 54 | hsa-miR-338-3p | 1050.98 |
| 55 | hsa-miR-23a-3p | 1024.262 |
| 56 | hsa-miR-101-3p | 1001.408 |
| 57 | hsa-miR-126-3p | 978.9712 |
| 58 | hsa-miR-15a-5p | 921.1897 |
| 59 | hsa-miR-132-3p | 885.1281 |
| 60 | hsa-let-7c-5p | 882.5081 |
| 61 | hsa-miR-7-5p | 862.6641 |
| 62 | hsa-miR-103a-3p | 841.0173 |

| | | |
|---|---|---|
| 63 | hsa-miR-181c-5p | 787.4481 |
| 64 | hsa-miR-136-3p | 783.6147 |
| 65 | hsa-miR-361-5p | 771.282 |
| 66 | hsa-miR-10b-5p | 760.0214 |
| 67 | hsa-miR-28-3p | 702.5677 |
| 68 | hsa-miR-410-3p | 702.5494 |
| 69 | hsa-miR-409-3p | 685.2376 |
| 70 | hsa-miR-24-3p | 665.2599 |
| 71 | hsa-miR-194-5p | 634.4039 |
| 72 | hsa-miR-429 | 634.0176 |
| 73 | hsa-miR-29b-3p | 612.2177 |
| 74 | hsa-miR-136-5p | 600.5248 |
| 75 | hsa-miR-484 | 580.7374 |
| 76 | hsa-miR-411-5p | 579.5778 |
| 77 | hsa-miR-432-5p | 542.7362 |
| 78 | hsa-miR-342-3p | 533.9539 |
| 79 | hsa-miR-3168 | 464.4544 |
| 80 | hsa-miR-181b-5p | 462.2785 |
| 81 | hsa-miR-320a | 460.6484 |
| 82 | hsa-miR-28-5p | 460.326 |
| 83 | hsa-miR-199a-3p;hsa-miR-199b-3p | 450.9452 |
| 84 | hsa-miR-376c-3p | 448.2679 |
| 85 | hsa-miR-148b-3p | 442.8233 |
| 86 | hsa-miR-199a-5p | 440.5607 |
| 87 | hsa-miR-130a-3p | 416.9796 |
| 88 | hsa-miR-146b-5p | 416.6273 |
| 89 | hsa-miR-744-5p | 414.4475 |
| 90 | hsa-miR-381-3p | 409.2744 |
| 91 | hsa-miR-221-3p | 389.9197 |
| 92 | hsa-miR-485-5p | 356.8929 |
| 93 | hsa-miR-574-3p | 335.9766 |
| 94 | hsa-miR-128-3p | 327.2629 |
| 95 | hsa-miR-301a-3p | 323.908 |
| 96 | hsa-miR-486-5p | 307.2739 |
| 97 | hsa-miR-421 | 301.1576 |
| 98 | hsa-miR-425-5p | 299.8963 |
| 99 | hsa-miR-874-3p | 280.8262 |
| 100 | hsa-miR-3607-5p | 266.8123 |
| 101 | hsa-miR-96-5p | 265.1244 |
| 102 | hsa-miR-15b-5p | 264.32 |
| 103 | hsa-miR-654-3p | 260.9603 |
| 104 | hsa-miR-132-5p | 259.2728 |
| 105 | hsa-miR-369-5p | 252.6895 |
| 106 | hsa-miR-25-3p | 244.4331 |
| 107 | hsa-miR-99a-5p | 234.7106 |

| | | |
|---|---|---|
| 108 | hsa-miR-212-3p | 227.6629 |
| 109 | hsa-miR-100-5p | 223.2627 |
| 110 | hsa-miR-21-3p | 220.5579 |
| 111 | hsa-miR-340-5p | 212.0131 |
| 112 | hsa-miR-1468-5p | 211.2405 |
| 113 | hsa-miR-145-5p | 209.1579 |
| 114 | hsa-miR-195-5p | 204.0097 |
| 115 | hsa-miR-374b-5p | 203.669 |
| 116 | hsa-miR-370-3p | 200.2546 |
| 117 | hsa-miR-151b | 200.0127 |
| 118 | hsa-miR-374a-5p | 197.7987 |
| 119 | hsa-miR-4532 | 195.8427 |
| 120 | hsa-miR-323b-3p | 195.0031 |
| 121 | hsa-miR-551b-3p | 194.8752 |
| 122 | hsa-miR-328-3p | 190.3496 |
| 123 | hsa-miR-335-3p | 189.8667 |
| 124 | hsa-miR-184 | 188.4126 |
| 125 | hsa-miR-95-3p | 184.5463 |
| 126 | hsa-miR-376a-5p | 166.8691 |
| 127 | hsa-miR-129-5p | 164.4993 |
| 128 | hsa-miR-141-5p | 163.9442 |
| 129 | hsa-miR-130b-3p | 159.6375 |
| 130 | hsa-miR-146a-5p | 157.7805 |
| 131 | hsa-miR-323a-3p | 150.3427 |
| 132 | hsa-miR-652-3p | 149.3716 |
| 133 | hsa-miR-19b-3p | 143.6241 |
| 134 | hsa-miR-212-5p | 142.6048 |
| 135 | hsa-miR-500a-3p | 134.731 |
| 136 | hsa-miR-369-3p | 134.3217 |
| 137 | hsa-miR-1180-3p | 131.1446 |
| 138 | hsa-miR-889-3p | 128.1077 |
| 139 | hsa-miR-125b-1-3p | 128.0477 |
| 140 | hsa-miR-345-5p | 125.1681 |
| 141 | hsa-miR-433-3p | 121.5622 |
| 142 | hsa-miR-199b-5p | 121.505 |
| 143 | hsa-miR-221-5p | 119.2459 |
| 144 | hsa-miR-197-3p | 114.1717 |
| 145 | hsa-miR-339-3p | 109.8324 |
| 146 | hsa-let-7d-3p | 107.9448 |
| 147 | hsa-miR-148b-5p | 105.2686 |
| 148 | hsa-miR-487a-3p | 98.8731 |
| 149 | hsa-miR-361-3p | 98.68969 |
| 150 | hsa-miR-1301-3p | 97.55109 |
| 151 | hsa-miR-129-2-3p | 97.11338 |
| 152 | hsa-miR-769-5p | 96.22528 |

| | | |
|---|---|---|
| 153 | hsa-miR-129-1-3p | 95.26058 |
| 154 | hsa-miR-877-5p | 93.3404 |
| 155 | hsa-miR-135a-5p | 91.57143 |
| 156 | hsa-miR-1224-5p | 91.12916 |
| 157 | hsa-miR-193b-3p | 90.42926 |
| 158 | hsa-miR-149-5p | 88.99439 |
| 159 | hsa-let-7b-3p | 82.94169 |
| 160 | hsa-miR-708-5p | 81.828 |
| 161 | hsa-miR-152-3p | 81.51372 |
| 162 | hsa-miR-454-3p | 80.11428 |
| 163 | hsa-miR-377-3p | 79.7154 |
| 164 | hsa-miR-670-3p | 79.67103 |
| 165 | hsa-miR-539-3p | 78.19656 |
| 166 | hsa-miR-301b-3p | 77.67412 |
| 167 | hsa-miR-142-5p | 75.30996 |
| 168 | hsa-miR-145-3p | 74.53333 |
| 169 | hsa-miR-200b-5p | 74.51927 |
| 170 | hsa-miR-32-5p | 74.00924 |
| 171 | hsa-miR-1260a | 73.77761 |
| 172 | hsa-miR-582-5p | 73.53756 |
| 173 | hsa-miR-532-5p | 71.16177 |
| 174 | hsa-miR-93-5p | 69.99367 |
| 175 | hsa-miR-941 | 69.54074 |
| 176 | hsa-miR-181d-5p | 68.97515 |
| 177 | hsa-miR-1307-5p | 67.25734 |
| 178 | hsa-miR-140-3p | 66.8573 |
| 179 | hsa-miR-577 | 66.56384 |
| 180 | hsa-miR-628-5p | 66.28473 |
| 181 | hsa-miR-330-5p | 65.08855 |
| 182 | hsa-miR-382-5p | 65.00645 |
| 183 | hsa-miR-340-3p | 64.85669 |
| 184 | hsa-miR-874-5p | 64.52381 |
| 185 | hsa-miR-1260b | 63.15415 |
| 186 | hsa-miR-27b-5p | 62.34446 |
| 187 | hsa-miR-148a-5p | 62.17515 |
| 188 | hsa-miR-134-5p | 62.12548 |
| 189 | hsa-miR-7977 | 61.56689 |
| 190 | hsa-miR-873-5p | 58.34816 |
| 191 | hsa-miR-107 | 57.95165 |
| 192 | hsa-miR-106b-5p | 57.86896 |
| 193 | hsa-miR-660-5p | 57.68272 |
| 194 | hsa-miR-30d-3p | 56.23378 |
| 195 | hsa-miR-424-5p | 56.18704 |
| 196 | hsa-miR-24-2-5p | 56.00229 |
| 197 | hsa-miR-130b-5p | 55.84246 |

| | | |
|---|---|---|
| 198 | hsa-miR-497-5p | 55.67386 |
| 199 | hsa-miR-3065-3p | 54.54768 |
| 200 | hsa-miR-224-5p | 54.25996 |
| 201 | hsa-miR-150-5p | 53.25289 |
| 202 | hsa-miR-331-3p | 53.22094 |
| 203 | hsa-miR-409-5p | 52.79447 |
| 204 | hsa-miR-214-3p | 51.43063 |
| 205 | hsa-miR-455-5p | 50.55013 |
| 206 | hsa-miR-363-3p | 50.31141 |
| 207 | hsa-miR-342-5p | 50.3087 |
| 208 | hsa-miR-26b-3p | 47.4227 |
| 209 | hsa-miR-494-3p | 45.72508 |
| 210 | hsa-miR-378a-3p | 45.58879 |
| 211 | hsa-miR-656-3p | 45.25702 |
| 212 | hsa-miR-17-5p | 44.92064 |
| 213 | hsa-miR-7-2-3p | 43.45663 |
| 214 | hsa-miR-539-5p | 43.37188 |
| 215 | hsa-miR-376a-3p | 43.36043 |
| 216 | hsa-miR-365a-3p;hsa-miR-365b-3p | 43.26143 |
| 217 | hsa-miR-216a-5p | 43.0022 |
| 218 | hsa-miR-1185-5p | 42.76405 |
| 219 | hsa-miR-29c-5p | 42.74648 |
| 220 | hsa-miR-758-3p | 42.49247 |
| 221 | hsa-miR-98-3p | 42.2561 |
| 222 | hsa-miR-210-3p | 42.17232 |
| 223 | hsa-miR-210-5p | 41.01511 |
| 224 | hsa-miR-455-3p | 40.64051 |
| 225 | hsa-miR-338-5p | 40.39432 |
| 226 | hsa-miR-374a-3p | 40.05364 |
| 227 | hsa-miR-320b | 40.03375 |
| 228 | hsa-miR-501-3p | 39.7748 |
| 229 | hsa-miR-125a-3p | 39.16676 |
| 230 | hsa-miR-668-3p | 38.82771 |
| 231 | hsa-miR-493-5p | 38.75496 |
| 232 | hsa-miR-574-5p | 37.7574 |
| 233 | hsa-miR-30a-3p | 37.64079 |
| 234 | hsa-let-7a-3p | 37.5029 |
| 235 | hsa-miR-133a-3p | 37.32967 |
| 236 | hsa-miR-34a-5p | 37.13697 |
| 237 | hsa-miR-30e-3p | 36.3578 |
| 238 | hsa-miR-181a-2-3p | 35.95816 |
| 239 | hsa-miR-1224-3p | 34.49647 |
| 240 | hsa-miR-489-3p | 33.58296 |
| 241 | hsa-miR-153-5p | 33.35186 |
| 242 | hsa-miR-642a-5p | 32.8564 |

| | | |
|---|---|---|
| 243 | hsa-miR-19a-3p | 32.62421 |
| 244 | hsa-miR-125b-2-3p | 32.39774 |
| 245 | hsa-miR-155-5p | 32.06561 |
| 246 | hsa-miR-802 | 31.35362 |
| 247 | hsa-miR-330-3p | 31.31229 |
| 248 | hsa-miR-183-3p | 30.80534 |
| 249 | hsa-miR-3609 | 30.45225 |
| 250 | hsa-miR-324-5p | 30.38663 |
| 251 | hsa-miR-1179 | 29.96565 |
| 252 | hsa-miR-7-1-3p | 28.95956 |
| 253 | hsa-miR-502-3p | 28.71689 |
| 254 | hsa-miR-485-3p | 28.49378 |
| 255 | hsa-miR-3653-5p | 28.33477 |
| 256 | hsa-miR-22-5p | 28.22219 |
| 257 | hsa-miR-488-3p | 27.91116 |
| 258 | hsa-miR-200a-5p | 27.66036 |
| 259 | hsa-miR-181a-3p | 27.55285 |
| 260 | hsa-miR-671-3p | 27.11773 |
| 261 | hsa-miR-589-5p | 26.92366 |
| 262 | hsa-miR-3065-5p | 26.90649 |
| 263 | hsa-miR-215-5p | 26.69396 |
| 264 | hsa-miR-664a-5p | 25.6745 |
| 265 | hsa-miR-3605-3p | 25.02164 |
| 266 | hsa-miR-93-3p | 25.01271 |
| 267 | hsa-miR-181c-3p | 24.8338 |
| 268 | hsa-miR-664a-3p | 24.70565 |
| 269 | hsa-miR-216b-5p | 24.51296 |
| 270 | hsa-miR-377-5p | 24.21876 |
| 271 | hsa-miR-532-3p | 23.97313 |
| 272 | hsa-miR-582-3p | 23.29594 |
| 273 | hsa-miR-20a-5p | 23.20789 |
| 274 | hsa-miR-127-5p | 23.13036 |
| 275 | hsa-miR-4662a-5p | 22.98047 |
| 276 | hsa-miR-17-3p | 22.9411 |
| 277 | hsa-miR-154-5p | 22.75385 |
| 278 | hsa-miR-24-1-5p | 22.46225 |
| 279 | hsa-let-7i-3p | 22.38448 |
| 280 | hsa-miR-411-3p | 22.24062 |
| 281 | hsa-miR-628-3p | 22.20333 |
| 282 | hsa-miR-324-3p | 22.16286 |
| 283 | hsa-miR-16-2-3p | 22.098 |
| 284 | hsa-miR-30c-2-3p | 21.78377 |
| 285 | hsa-miR-431-5p | 21.70005 |
| 286 | hsa-miR-1307-3p | 21.37684 |
| 287 | hsa-miR-493-3p | 21.32846 |

| | | |
|---|---|---|
| 288 | hsa-miR-139-5p | 21.32769 |
| 289 | hsa-miR-31-5p | 21.14837 |
| 290 | hsa-miR-671-5p | 21.10829 |
| 291 | hsa-miR-33b-5p | 21.0212 |
| 292 | hsa-miR-590-3p | 20.08197 |
| 293 | hsa-miR-29a-5p | 19.61487 |
| 294 | hsa-miR-625-5p | 19.23082 |
| 295 | hsa-miR-379-5p | 19.14283 |
| 296 | hsa-miR-380-3p | 19.06113 |
| 297 | hsa-miR-495-3p | 17.83986 |
| 298 | hsa-miR-337-3p | 17.62032 |
| 299 | hsa-miR-3200-3p | 16.69835 |
| 300 | hsa-miR-450a-5p | 16.68526 |
| 301 | hsa-miR-1185-1-3p | 16.63242 |
| 302 | hsa-miR-6511a-3p | 16.54646 |
| 303 | hsa-miR-106b-3p | 16.29426 |
| 304 | hsa-miR-147b | 16.18079 |
| 305 | hsa-miR-2110 | 16.16959 |
| 306 | hsa-miR-190b | 16.15072 |
| 307 | hsa-miR-654-5p | 16.00814 |
| 308 | hsa-miR-29b-2-5p | 15.71947 |
| 309 | hsa-miR-190a-5p | 15.29524 |
| 310 | hsa-miR-33a-5p | 15.18456 |
| 311 | hsa-miR-873-3p | 15.18317 |
| 312 | hsa-miR-543 | 15.13923 |
| 313 | hsa-miR-101-5p | 15.02166 |
| 314 | hsa-miR-1246 | 14.92316 |
| 315 | hsa-miR-642a-3p | 14.5348 |
| 316 | hsa-miR-25-5p | 14.5339 |
| 317 | hsa-miR-1296-5p | 13.99203 |
| 318 | hsa-miR-425-3p | 13.95324 |
| 319 | hsa-miR-193a-3p | 13.83026 |
| 320 | hsa-miR-451a | 13.76736 |
| 321 | hsa-miR-708-3p | 13.67939 |
| 322 | hsa-miR-185-5p | 13.629 |
| 323 | hsa-miR-431-3p | 13.42542 |
| 324 | hsa-miR-641 | 13.36364 |
| 325 | hsa-miR-329-3p | 13.33643 |
| 326 | hsa-miR-6511b-3p | 13.28732 |
| 327 | hsa-let-7e-3p | 12.64904 |
| 328 | hsa-miR-3615 | 12.30614 |
| 329 | hsa-miR-3607-3p | 12.18835 |
| 330 | hsa-miR-550a-5p | 11.9776 |
| 331 | hsa-miR-450b-5p | 11.66544 |
| 332 | hsa-miR-99b-3p | 11.54355 |

| | | |
|---|---|---|
| 333 | hsa-let-7f-1-3p | 11.39884 |
| 334 | hsa-miR-320c | 11.37636 |
| 335 | hsa-miR-142-3p | 11.26409 |
| 336 | hsa-miR-214-5p | 11.06385 |
| 337 | hsa-miR-598-3p | 10.94106 |
| 338 | hsa-miR-140-5p | 10.71556 |
| 339 | hsa-miR-337-5p | 10.32129 |
| 340 | hsa-miR-193a-5p | 10.20051 |
| 341 | hsa-miR-379-3p | 10.06474 |
| 342 | hsa-let-7f-2-3p | 9.969011 |
| 343 | hsa-miR-296-5p | 9.895999 |
| 344 | hsa-miR-30c-1-3p | 9.509561 |
| 345 | hsa-miR-192-3p | 9.501948 |
| 346 | hsa-miR-362-5p | 9.42444 |
| 347 | hsa-miR-376b-3p | 9.330562 |
| 348 | hsa-miR-331-5p | 9.22495 |
| 349 | hsa-let-7g-3p | 8.992349 |
| 350 | hsa-miR-203a-3p | 8.929635 |
| 351 | hsa-miR-137 | 8.927235 |
| 352 | hsa-miR-744-3p | 8.817853 |
| 353 | hsa-miR-544a | 8.790243 |
| 354 | hsa-miR-320d | 8.71815 |
| 355 | hsa-miR-3620-3p | 8.654056 |
| 356 | hsa-miR-326 | 8.641181 |
| 357 | hsa-miR-92b-5p | 8.528413 |
| 358 | hsa-miR-576-5p | 8.244863 |
| 359 | hsa-miR-95-5p | 8.232537 |
| 360 | hsa-miR-496 | 8.181449 |
| 361 | hsa-miR-339-5p | 8.122852 |
| 362 | hsa-miR-505-3p | 8.066493 |
| 363 | hsa-miR-30b-3p | 8.010539 |
| 364 | hsa-miR-551b-5p | 7.879253 |
| 365 | hsa-miR-7706 | 7.878805 |
| 366 | hsa-miR-548k | 7.690402 |
| 367 | hsa-miR-7641 | 7.504929 |
| 368 | hsa-miR-1197 | 7.273231 |
| 369 | hsa-miR-665 | 7.161204 |
| 370 | hsa-miR-382-3p | 7.013789 |
| 371 | hsa-miR-217 | 6.982153 |
| 372 | hsa-miR-483-3p | 6.947304 |
| 373 | hsa-miR-770-5p | 6.947068 |
| 374 | hsa-miR-92a-1-5p | 6.903531 |
| 375 | hsa-miR-655-3p | 6.780498 |
| 376 | hsa-miR-200c-5p | 6.76446 |
| 377 | hsa-miR-592 | 6.728438 |

| | | |
|---|---|---|
| 378 | hsa-miR-1229-3p | 6.569406 |
| 379 | hsa-miR-1247-5p | 6.568963 |
| 380 | hsa-miR-191-3p | 6.548826 |
| 381 | hsa-miR-3613-3p | 6.508481 |
| 382 | hsa-miR-4454 | 6.202206 |
| 383 | hsa-miR-1343-3p | 6.17674 |
| 384 | hsa-miR-10a-3p | 6.031309 |
| 385 | hsa-miR-222-5p | 5.941375 |
| 386 | hsa-miR-4531 | 5.920394 |
| 387 | hsa-miR-500a-5p | 5.825951 |
| 388 | hsa-miR-135b-5p | 5.752092 |
| 389 | hsa-miR-187-3p | 5.560705 |
| 390 | hsa-miR-550a-3p | 5.528757 |
| 391 | hsa-miR-223-3p | 5.477771 |
| 392 | hsa-miR-1306-5p | 5.477164 |
| 393 | hsa-miR-1248 | 5.433697 |
| 394 | hsa-miR-143-5p | 5.398236 |
| 395 | hsa-miR-138-5p | 5.392248 |
| 396 | hsa-miR-1275 | 5.381648 |
| 397 | hsa-miR-27a-5p | 5.363287 |
| 398 | hsa-miR-323a-5p | 5.361184 |
| 399 | hsa-miR-885-5p | 5.279463 |
| 400 | hsa-miR-26a-2-3p | 5.265377 |
| 401 | hsa-miR-454-5p | 5.192823 |
| 402 | hsa-miR-374b-3p | 5.136824 |
| 403 | hsa-miR-889-5p | 5.080676 |
| 404 | hsa-miR-299-3p | 5.053115 |
| 405 | hsa-miR-6777-3p | 5.024582 |
| 406 | hsa-miR-99a-3p | 5.017013 |
| 407 | hsa-miR-146b-3p | 5.012638 |
| 408 | hsa-miR-182-3p | 4.968011 |
| 409 | hsa-miR-18a-5p | 4.902247 |
| 410 | hsa-miR-1287-5p | 4.870462 |
| 411 | hsa-miR-3605-5p | 4.836553 |
| 412 | hsa-miR-296-3p | 4.805936 |
| 413 | hsa-miR-424-3p | 4.786531 |
| 414 | hsa-miR-483-5p | 4.771509 |
| 415 | hsa-miR-23a-5p | 4.749555 |
| 416 | hsa-miR-760 | 4.639648 |
| 417 | hsa-miR-5010-3p | 4.588924 |
| 418 | hsa-miR-186-3p | 4.573589 |
| 419 | hsa-miR-652-5p | 4.555055 |
| 420 | hsa-miR-1303 | 4.532499 |
| 421 | hsa-miR-1291 | 4.426293 |
| 422 | hsa-miR-499a-5p | 4.363371 |

| | | |
|---|---|---|
| 423 | hsa-miR-432-3p | 4.342381 |
| 424 | hsa-miR-6087 | 4.321139 |
| 425 | hsa-miR-452-5p | 4.309456 |
| 426 | hsa-miR-3613-5p | 3.993103 |
| 427 | hsa-miR-188-5p | 3.945814 |
| 428 | hsa-miR-625-3p | 3.919023 |
| 429 | hsa-miR-3909 | 3.917365 |
| 430 | hsa-miR-20a-3p | 3.814003 |
| 431 | hsa-miR-758-5p | 3.679236 |
| 432 | hsa-miR-32-3p | 3.643751 |
| 433 | hsa-miR-34a-3p | 3.628868 |
| 434 | hsa-miR-561-5p | 3.619861 |
| 435 | hsa-miR-2355-5p | 3.594528 |
| 436 | hsa-miR-589-3p | 3.56405 |
| 437 | hsa-miR-1270 | 3.504337 |
| 438 | hsa-miR-204-3p | 3.437531 |
| 439 | hsa-miR-542-3p | 3.36965 |
| 440 | hsa-miR-1251-5p | 3.349026 |
| 441 | hsa-miR-15b-3p | 3.319333 |
| 442 | hsa-miR-433-5p | 3.295895 |
| 443 | hsa-miR-134-3p | 3.29106 |
| 444 | hsa-miR-548e-3p | 3.199547 |
| 445 | hsa-miR-487a-5p | 3.171541 |
| 446 | hsa-miR-7704 | 3.161717 |
| 447 | hsa-miR-224-3p | 3.149555 |
| 448 | hsa-miR-767-5p | 3.144238 |
| 449 | hsa-miR-1299 | 3.069218 |
| 450 | hsa-miR-33b-3p | 3.031384 |
| 451 | hsa-miR-194-3p | 3.027674 |
| 452 | hsa-miR-1285-3p | 2.998341 |
| 453 | hsa-miR-891a-5p | 2.972907 |
| 454 | hsa-miR-487b-5p | 2.911322 |
| 455 | hsa-miR-4516 | 2.904456 |
| 456 | hsa-miR-362-3p | 2.896125 |
| 457 | hsa-miR-766-3p | 2.873589 |
| 458 | hsa-miR-3929 | 2.852098 |
| 459 | hsa-miR-29b-1-5p | 2.814485 |
| 460 | hsa-miR-370-5p | 2.808985 |
| 461 | hsa-miR-3177-3p | 2.752974 |
| 462 | hsa-miR-195-3p | 2.72392 |
| 463 | hsa-miR-502-5p | 2.706136 |
| 464 | hsa-miR-18a-3p | 2.666454 |
| 465 | hsa-miR-940 | 2.645595 |
| 466 | hsa-miR-218-5p | 2.611469 |
| 467 | hsa-miR-501-5p | 2.537314 |

| | | |
|---|---|---|
| 468 | hsa-miR-548o-3p | 2.527315 |
| 469 | hsa-miR-34c-5p | 2.524795 |
| 470 | hsa-miR-23b-5p | 2.517781 |
| 471 | hsa-miR-584-5p | 2.505631 |
| 472 | hsa-miR-3200-5p | 2.470706 |
| 473 | hsa-miR-653-3p | 2.427527 |
| 474 | hsa-miR-491-5p | 2.392188 |
| 475 | hsa-miR-4521 | 2.380687 |
| 476 | hsa-miR-545-5p | 2.367688 |
| 477 | hsa-miR-4326 | 2.357713 |
| 478 | hsa-miR-381-5p | 2.33717 |
| 479 | hsa-miR-541-5p | 2.333066 |
| 480 | hsa-miR-664b-5p | 2.288804 |
| 481 | hsa-miR-6747-3p | 2.249931 |
| 482 | hsa-miR-887-3p | 2.211713 |
| 483 | hsa-miR-4640-3p | 2.205228 |
| 484 | hsa-miR-6868-3p | 2.199983 |
| 485 | hsa-miR-128-1-5p | 2.188552 |
| 486 | hsa-miR-4758-3p | 2.170908 |
| 487 | hsa-miR-26a-1-3p | 2.163379 |
| 488 | hsa-miR-185-3p | 2.144563 |
| 489 | hsa-miR-616-5p | 2.138548 |
| 490 | hsa-miR-216a-3p | 2.119266 |
| 491 | hsa-miR-1271-5p | 2.072021 |
| 492 | hsa-miR-383-5p | 2.064889 |
| 493 | hsa-miR-1277-3p | 2.06409 |
| 494 | hsa-miR-3687 | 2.046264 |
| 495 | hsa-miR-505-5p | 2.036985 |
| 496 | hsa-miR-1-3p | 2.030266 |
| 497 | hsa-miR-9-5p | 2.004368 |
| 498 | hsa-miR-627-5p | 1.981009 |
| 499 | hsa-miR-877-3p | 1.979945 |
| 500 | hsa-miR-412-5p | 1.97257 |
| 501 | hsa-miR-188-3p | 1.911719 |
| 502 | hsa-miR-542-5p | 1.899967 |
| 503 | hsa-miR-144-3p | 1.89128 |
| 504 | hsa-miR-6726-3p | 1.856827 |
| 505 | hsa-miR-3661 | 1.847362 |
| 506 | hsa-miR-6842-3p | 1.836665 |
| 507 | hsa-miR-1185-2-3p | 1.82375 |
| 508 | hsa-miR-4677-3p | 1.816653 |
| 509 | hsa-miR-1254 | 1.814768 |
| 510 | hsa-miR-876-5p | 1.802726 |
| 511 | hsa-miR-219a-5p | 1.763754 |
| 512 | hsa-miR-937-3p | 1.742801 |

| | | |
|---|---|---|
| 513 | hsa-miR-5010-5p | 1.731093 |
| 514 | hsa-miR-942-5p | 1.728841 |
| 515 | hsa-miR-4449 | 1.724728 |
| 516 | hsa-miR-4446-3p | 1.723959 |
| 517 | hsa-miR-503-5p | 1.659881 |
| 518 | hsa-let-7c-3p | 1.654107 |
| 519 | hsa-miR-576-3p | 1.647872 |
| 520 | hsa-miR-4787-3p | 1.64733 |
| 521 | hsa-miR-376a-2-5p | 1.63012 |
| 522 | hsa-miR-4508 | 1.618948 |
| 523 | hsa-miR-1237-3p | 1.603398 |
| 524 | hsa-miR-105-5p | 1.590368 |
| 525 | hsa-miR-3158-3p | 1.588371 |
| 526 | hsa-miR-23c | 1.571701 |
| 527 | hsa-miR-664b-3p | 1.539274 |
| 528 | hsa-miR-5683 | 1.537251 |
| 529 | hsa-miR-486-3p | 1.53575 |
| 530 | hsa-miR-10b-3p | 1.529692 |
| 531 | hsa-miR-3656 | 1.524156 |
| 532 | hsa-miR-6803-3p | 1.49362 |
| 533 | hsa-miR-208b-3p | 1.485615 |
| 534 | hsa-miR-193b-5p | 1.484972 |
| 535 | hsa-miR-1233-3p | 1.465644 |
| 536 | hsa-miR-365b-5p | 1.450471 |
| 537 | hsa-miR-5701 | 1.445765 |
| 538 | hsa-miR-548w | 1.434132 |
| 539 | hsa-miR-6732-3p | 1.433627 |
| 540 | hsa-miR-488-5p | 1.418642 |
| 541 | hsa-miR-629-5p | 1.41791 |
| 542 | hsa-miR-548b-5p | 1.413389 |
| 543 | hsa-miR-301a-5p | 1.410721 |
| 544 | hsa-miR-2277-5p | 1.341934 |
| 545 | hsa-miR-6894-3p | 1.324768 |
| 546 | hsa-miR-2116-3p | 1.321477 |
| 547 | hsa-miR-5586-5p | 1.303409 |
| 548 | hsa-miR-618 | 1.29923 |
| 549 | hsa-miR-548e-5p | 1.278643 |
| 550 | hsa-miR-541-3p | 1.277382 |
| 551 | hsa-miR-122-5p | 1.270975 |
| 552 | hsa-miR-1268b | 1.269733 |
| 553 | hsa-miR-3117-3p | 1.261635 |
| 554 | hsa-miR-1304-3p | 1.256551 |
| 555 | hsa-miR-876-3p | 1.248525 |
| 556 | hsa-miR-545-3p | 1.244033 |
| 557 | hsa-miR-3928-3p | 1.240214 |

| | | |
|---|---|---|
| 558 | hsa-miR-6516-5p | 1.239716 |
| 559 | hsa-miR-33a-3p | 1.22118 |
| 560 | hsa-miR-299-5p | 1.21675 |
| 561 | hsa-miR-494-5p | 1.204777 |
| 562 | hsa-miR-1268a | 1.192532 |
| 563 | hsa-miR-5001-3p | 1.182877 |
| 564 | hsa-miR-6750-3p | 1.182525 |
| 565 | hsa-miR-1226-3p | 1.177722 |
| 566 | hsa-miR-100-3p | 1.161958 |
| 567 | hsa-miR-152-5p | 1.160342 |
| 568 | hsa-miR-548ad-5p;hsa-miR-548ae-5p | 1.135088 |
| 569 | hsa-miR-4742-3p | 1.11781 |
| 570 | hsa-miR-3912-3p | 1.109618 |
| 571 | hsa-miR-329-5p | 1.068131 |
| 572 | hsa-miR-4520-2-3p | 1.051561 |
| 573 | hsa-miR-1976 | 1.044054 |
| 574 | hsa-miR-4324 | 1.044031 |
| 575 | hsa-miR-1908-5p | 1.040113 |
| 576 | hsa-miR-181b-3p | 1.034741 |
| 577 | hsa-miR-410-5p | 1.034453 |
| 578 | hsa-miR-5100 | 1.02489 |
| 579 | hsa-miR-154-3p | 1.019373 |
| 580 | hsa-miR-1255a | 1.017413 |
| 581 | hsa-miR-2681-5p | 1.016302 |
| 582 | hsa-miR-3653-3p | 1.009672 |
| 583 | hsa-miR-6735-5p | 0.970203 |
| 584 | hsa-miR-548b-3p | 0.96537 |
| 585 | hsa-miR-4685-3p | 0.95478 |
| 586 | hsa-miR-3934-5p | 0.954536 |
| 587 | hsa-miR-4742-5p | 0.953046 |
| 588 | hsa-miR-6516-3p | 0.942763 |
| 589 | hsa-miR-655-5p | 0.942298 |
| 590 | hsa-miR-6880-3p | 0.929999 |
| 591 | hsa-miR-190a-3p | 0.922794 |
| 592 | hsa-miR-4690-3p | 0.922042 |
| 593 | hsa-miR-1249-3p | 0.918092 |
| 594 | hsa-miR-4705 | 0.913973 |
| 595 | hsa-miR-769-3p | 0.9056 |
| 596 | hsa-miR-1227-3p | 0.903679 |
| 597 | hsa-miR-1323 | 0.897889 |
| 598 | hsa-miR-1306-3p | 0.895645 |
| 599 | hsa-miR-4492 | 0.888062 |
| 600 | hsa-miR-6770-3p | 0.884716 |
| 601 | hsa-miR-378c | 0.882512 |

| | | |
|---|---|---|
| 602 | hsa-miR-2276-3p | 0.87506 |
| 603 | hsa-miR-6749-3p | 0.865991 |
| 604 | hsa-miR-6802-3p | 0.862296 |
| 605 | hsa-miR-651-5p | 0.859525 |
| 606 | hsa-miR-6772-3p | 0.838458 |
| 607 | hsa-miR-660-3p | 0.827881 |
| 608 | hsa-miR-3074-3p | 0.824761 |
| 609 | hsa-miR-643 | 0.824596 |
| 610 | hsa-miR-6895-3p | 0.80775 |
| 611 | hsa-miR-4781-3p | 0.80691 |
| 612 | hsa-miR-3619-5p | 0.801859 |
| 613 | hsa-miR-6853-3p | 0.790505 |
| 614 | hsa-miR-6818-5p | 0.786961 |
| 615 | hsa-miR-6819-3p | 0.786457 |
| 616 | hsa-miR-2355-3p | 0.777191 |
| 617 | hsa-miR-548i | 0.773003 |
| 618 | hsa-miR-935 | 0.764695 |
| 619 | hsa-miR-3161 | 0.761698 |
| 620 | hsa-miR-3611 | 0.754905 |
| 621 | hsa-miR-3173-5p | 0.750099 |
| 622 | hsa-miR-1908-3p | 0.747826 |
| 623 | hsa-miR-3651 | 0.747584 |
| 624 | hsa-miR-7974 | 0.746084 |
| 625 | hsa-miR-373-3p | 0.739182 |
| 626 | hsa-miR-550a-3-5p | 0.738841 |
| 627 | hsa-miR-6786-3p | 0.733957 |
| 628 | hsa-miR-6505-3p | 0.733047 |
| 629 | hsa-miR-106a-5p | 0.732656 |
| 630 | hsa-miR-3064-5p | 0.726801 |
| 631 | hsa-miR-6758-3p | 0.720832 |
| 632 | hsa-miR-659-5p | 0.71343 |
| 633 | hsa-miR-216b-3p | 0.71263 |
| 634 | hsa-miR-6769b-3p | 0.712566 |
| 635 | hsa-miR-670-5p | 0.711529 |
| 636 | hsa-miR-3187-3p | 0.710387 |
| 637 | hsa-miR-5006-3p | 0.708084 |
| 638 | hsa-miR-4646-3p | 0.705111 |
| 639 | hsa-miR-4749-3p | 0.69513 |
| 640 | hsa-miR-144-5p | 0.67966 |
| 641 | hsa-miR-676-3p | 0.672435 |
| 642 | hsa-miR-219a-1-3p | 0.666505 |
| 643 | hsa-miR-4689 | 0.665181 |
| 644 | hsa-miR-656-5p | 0.660106 |
| 645 | hsa-miR-3180-5p | 0.659345 |
| 646 | hsa-miR-3074-5p | 0.647674 |

| | | |
|---|---|---|
| 647 | hsa-miR-6812-3p | 0.646222 |
| 648 | hsa-miR-4728-3p | 0.639836 |
| 649 | hsa-miR-7155-3p | 0.637377 |
| 650 | hsa-miR-1262 | 0.633496 |
| 651 | hsa-miR-6737-3p | 0.631151 |
| 652 | hsa-miR-4488 | 0.629507 |
| 653 | hsa-miR-16-1-3p | 0.625367 |
| 654 | hsa-miR-504-5p | 0.624231 |
| 655 | hsa-miR-4423-5p | 0.620968 |
| 656 | hsa-miR-6855-3p | 0.616193 |
| 657 | hsa-miR-3913-5p | 0.614246 |
| 658 | hsa-miR-4671-3p | 0.611511 |
| 659 | hsa-miR-675-3p | 0.611354 |
| 660 | hsa-miR-1276 | 0.610075 |
| 661 | hsa-miR-6866-5p | 0.604949 |
| 662 | hsa-miR-449b-5p | 0.601424 |
| 663 | hsa-miR-103a-2-5p | 0.600747 |
| 664 | hsa-miR-4796-3p | 0.597005 |
| 665 | hsa-miR-6514-3p | 0.59283 |
| 666 | hsa-miR-2682-5p | 0.585816 |
| 667 | hsa-miR-3679-5p | 0.584222 |
| 668 | hsa-miR-6741-3p | 0.584038 |
| 669 | hsa-miR-4423-3p | 0.579495 |
| 670 | hsa-miR-7705 | 0.571887 |
| 671 | hsa-miR-495-5p | 0.570142 |
| 672 | hsa-miR-548a-5p | 0.566286 |
| 673 | hsa-miR-3127-3p | 0.562357 |
| 674 | hsa-miR-6505-5p | 0.559104 |
| 675 | hsa-miR-6511b-5p | 0.554505 |
| 676 | hsa-miR-3127-5p | 0.550867 |
| 677 | hsa-miR-642b-3p | 0.549818 |
| 678 | hsa-miR-3195 | 0.545083 |
| 679 | hsa-miR-6886-5p | 0.544438 |
| 680 | hsa-miR-885-3p | 0.542618 |
| 681 | hsa-miR-139-3p | 0.534003 |
| 682 | hsa-miR-3648 | 0.533545 |
| 683 | hsa-miR-548az-5p | 0.530804 |
| 684 | hsa-miR-376b-5p | 0.530038 |
| 685 | hsa-miR-376c-5p | 0.530038 |
| 686 | hsa-miR-548q | 0.529957 |
| 687 | hsa-miR-4662a-3p | 0.529027 |
| 688 | hsa-miR-5699-5p | 0.523571 |
| 689 | hsa-miR-4286 | 0.520287 |
| 690 | hsa-miR-4497 | 0.518283 |
| 691 | hsa-miR-590-5p | 0.516948 |

| | | |
|---|---|---|
| 692 | hsa-miR-4520-3p | 0.511926 |
| 693 | hsa-miR-5581-3p | 0.50807 |
| 694 | hsa-miR-6743-3p | 0.507728 |
| 695 | hsa-miR-4501 | 0.507708 |
| 696 | hsa-miR-1266-5p | 0.507228 |
| 697 | hsa-miR-6794-3p | 0.491487 |
| 698 | hsa-miR-365a-5p | 0.486265 |
| 699 | hsa-miR-6862-5p | 0.478226 |
| 700 | hsa-miR-6852-5p | 0.477411 |
| 701 | hsa-miR-615-3p | 0.475338 |
| 702 | hsa-miR-2114-5p | 0.47367 |
| 703 | hsa-miR-943 | 0.469473 |
| 704 | hsa-miR-548ab | 0.467684 |
| 705 | hsa-miR-7854-3p | 0.467238 |
| 706 | hsa-miR-1292-5p | 0.462147 |
| 707 | hsa-miR-548at-5p | 0.461931 |
| 708 | hsa-miR-570-3p | 0.455934 |
| 709 | hsa-miR-4723-3p | 0.451985 |
| 710 | hsa-miR-511-5p | 0.444891 |
| 711 | hsa-miR-5695 | 0.441613 |
| 712 | hsa-miR-4443 | 0.439628 |
| 713 | hsa-miR-598-5p | 0.434078 |
| 714 | hsa-miR-6883-3p | 0.432657 |
| 715 | hsa-miR-3664-3p | 0.426145 |
| 716 | hsa-miR-135a-3p | 0.4231 |
| 717 | hsa-miR-7114-3p | 0.422862 |
| 718 | hsa-miR-548a-3p | 0.421095 |
| 719 | hsa-miR-3936 | 0.418362 |
| 720 | hsa-miR-205-5p | 0.406975 |
| 721 | hsa-miR-3194-5p | 0.405633 |
| 722 | hsa-miR-6129 | 0.401078 |
| 723 | hsa-miR-2116-5p | 0.399362 |
| 724 | hsa-miR-4636 | 0.398521 |
| 725 | hsa-miR-6844 | 0.394824 |
| 726 | hsa-miR-6810-3p | 0.393491 |
| 727 | hsa-miR-4775 | 0.393039 |
| 728 | hsa-miR-5586-3p | 0.389567 |
| 729 | hsa-miR-1261 | 0.386556 |
| 730 | hsa-miR-2681-3p | 0.383896 |
| 731 | hsa-miR-6838-5p | 0.380945 |
| 732 | hsa-miR-6837-3p | 0.379101 |
| 733 | hsa-miR-548au-5p | 0.375475 |
| 734 | hsa-miR-19a-5p | 0.370469 |
| 735 | hsa-miR-516b-5p | 0.369612 |
| 736 | hsa-miR-6500-3p | 0.36737 |

| | | |
|---|---|---|
| 737 | hsa-miR-519c-3p | 0.367326 |
| 738 | hsa-miR-1538 | 0.366529 |
| 739 | hsa-miR-4802-3p | 0.365014 |
| 740 | hsa-miR-4682 | 0.36425 |
| 741 | hsa-miR-765 | 0.362886 |
| 742 | hsa-miR-197-5p | 0.361554 |
| 743 | hsa-miR-6843-3p | 0.36107 |
| 744 | hsa-miR-6777-5p | 0.360337 |
| 745 | hsa-miR-491-3p | 0.35888 |
| 746 | hsa-miR-3157-3p | 0.356729 |
| 747 | hsa-miR-4667-5p | 0.355154 |
| 748 | hsa-miR-548v | 0.353231 |
| 749 | hsa-miR-6757-3p | 0.352056 |
| 750 | hsa-miR-6716-3p | 0.351747 |
| 751 | hsa-miR-1234-3p | 0.347384 |
| 752 | hsa-miR-34b-3p | 0.347373 |
| 753 | hsa-miR-6798-3p | 0.346292 |
| 754 | hsa-miR-6859-5p | 0.345194 |
| 755 | hsa-miR-4466 | 0.344378 |
| 756 | hsa-miR-6514-5p | 0.340271 |
| 757 | hsa-miR-1180-5p | 0.340006 |
| 758 | hsa-miR-449a | 0.339183 |
| 759 | hsa-miR-3174 | 0.338853 |
| 760 | hsa-miR-6775-3p | 0.338511 |
| 761 | hsa-miR-1256 | 0.336779 |
| 762 | hsa-miR-548ay-3p | 0.336414 |
| 763 | hsa-miR-624-5p | 0.332999 |
| 764 | hsa-miR-7110-3p | 0.332273 |
| 765 | hsa-miR-4485-5p | 0.330977 |
| 766 | hsa-miR-2114-3p | 0.33079 |
| 767 | hsa-miR-323b-5p | 0.330531 |
| 768 | hsa-miR-5680 | 0.328319 |
| 769 | hsa-miR-4641 | 0.327382 |
| 770 | hsa-miR-4767 | 0.325989 |
| 771 | hsa-miR-20b-5p | 0.325604 |
| 772 | hsa-miR-4738-3p | 0.324131 |
| 773 | hsa-miR-346 | 0.323332 |
| 774 | hsa-miR-4485-3p | 0.32153 |
| 775 | hsa-miR-548ba | 0.318133 |
| 776 | hsa-miR-6820-5p | 0.317867 |
| 777 | hsa-miR-4671-5p | 0.313912 |
| 778 | hsa-miR-616-3p | 0.311644 |
| 779 | hsa-miR-636 | 0.310519 |
| 780 | hsa-miR-3940-3p | 0.309477 |
| 781 | hsa-miR-580-3p | 0.309402 |

| | | |
|---|---|---|
| 782 | hsa-miR-4726-5p | 0.30338 |
| 783 | hsa-miR-6885-5p | 0.302792 |
| 784 | hsa-miR-5699-3p | 0.302455 |
| 785 | hsa-miR-1284 | 0.296086 |
| 786 | hsa-miR-675-5p | 0.295687 |
| 787 | hsa-miR-4763-5p | 0.291699 |
| 788 | hsa-miR-6796-3p | 0.291271 |
| 789 | hsa-miR-3176 | 0.290881 |
| 790 | hsa-miR-4688 | 0.290063 |
| 791 | hsa-miR-1226-5p | 0.288836 |
| 792 | hsa-miR-219b-5p | 0.287725 |
| 793 | hsa-miR-1253 | 0.28767 |
| 794 | hsa-miR-663b | 0.286942 |
| 795 | hsa-miR-3196 | 0.285212 |
| 796 | hsa-miR-3622a-5p | 0.28344 |
| 797 | hsa-miR-4745-5p | 0.282406 |
| 798 | hsa-miR-6833-3p | 0.27927 |
| 799 | hsa-miR-2277-3p | 0.276797 |
| 800 | hsa-miR-1914-5p | 0.275155 |
| 801 | hsa-miR-509-3p | 0.274486 |
| 802 | hsa-miR-6826-3p | 0.274024 |
| 803 | hsa-miR-516a-5p | 0.270672 |
| 804 | hsa-miR-196a-5p | 0.270556 |
| 805 | hsa-miR-4662b | 0.268781 |
| 806 | hsa-miR-6805-5p | 0.268708 |
| 807 | hsa-miR-548j-3p | 0.268294 |
| 808 | hsa-miR-6765-3p | 0.267727 |
| 809 | hsa-miR-8072 | 0.267168 |
| 810 | hsa-miR-4510 | 0.266039 |
| 811 | hsa-miR-6816-3p | 0.264549 |
| 812 | hsa-miR-766-5p | 0.260963 |
| 813 | hsa-miR-6762-3p | 0.259695 |
| 814 | hsa-miR-7111-3p | 0.258561 |
| 815 | hsa-miR-208a-3p | 0.25721 |
| 816 | hsa-miR-380-5p | 0.25669 |
| 817 | hsa-miR-3140-3p | 0.255713 |
| 818 | hsa-miR-449c-5p | 0.253879 |
| 819 | hsa-miR-6851-5p | 0.253275 |
| 820 | hsa-miR-6742-3p | 0.251616 |
| 821 | hsa-miR-6827-3p | 0.24935 |
| 822 | hsa-miR-6763-3p | 0.248114 |
| 823 | hsa-miR-6791-3p | 0.244838 |
| 824 | hsa-miR-5588-5p | 0.244595 |
| 825 | hsa-miR-6794-5p | 0.242706 |
| 826 | hsa-miR-1193 | 0.241018 |

| | | |
|---|---|---|
| 827 | hsa-miR-1296-3p | 0.240758 |
| 828 | hsa-miR-629-3p | 0.239282 |
| 829 | hsa-miR-548s | 0.238313 |
| 830 | hsa-miR-3942-3p | 0.238178 |
| 831 | hsa-miR-520f-3p | 0.237605 |
| 832 | hsa-miR-6721-5p | 0.235513 |
| 833 | hsa-miR-6818-3p | 0.235046 |
| 834 | hsa-miR-5187-3p | 0.233713 |
| 835 | hsa-miR-548al | 0.23192 |
| 836 | hsa-miR-6894-5p | 0.231522 |
| 837 | hsa-miR-6733-5p | 0.229052 |
| 838 | hsa-miR-624-3p | 0.228849 |
| 839 | hsa-miR-18b-5p | 0.228555 |
| 840 | hsa-miR-5587-3p | 0.226967 |
| 841 | hsa-miR-512-3p | 0.226622 |
| 842 | hsa-miR-3620-5p | 0.226401 |
| 843 | hsa-miR-7850-5p | 0.226401 |
| 844 | hsa-miR-3145-3p | 0.224544 |
| 845 | hsa-miR-378a-5p | 0.223178 |
| 846 | hsa-miR-138-1-3p | 0.222389 |
| 847 | hsa-miR-4484 | 0.221772 |
| 848 | hsa-miR-19b-1-5p | 0.219587 |
| 849 | hsa-miR-6885-3p | 0.21656 |
| 850 | hsa-miR-663a | 0.216025 |
| 851 | hsa-miR-6866-3p | 0.215444 |
| 852 | hsa-miR-5696 | 0.215398 |
| 853 | hsa-miR-371b-5p | 0.215087 |
| 854 | hsa-miR-548f-3p | 0.212904 |
| 855 | hsa-miR-187-5p | 0.212203 |
| 856 | hsa-miR-517a-3p;hsa-miR-517b-3p | 0.211185 |
| 857 | hsa-miR-6822-5p | 0.210811 |
| 858 | hsa-miR-4461 | 0.209394 |
| 859 | hsa-miR-514a-3p | 0.208443 |
| 860 | hsa-miR-556-3p | 0.206772 |
| 861 | hsa-miR-4802-5p | 0.206421 |
| 862 | hsa-miR-518c-3p | 0.20589 |
| 863 | hsa-miR-4687-5p | 0.20555 |
| 864 | hsa-miR-412-3p | 0.204982 |
| 865 | hsa-miR-6807-3p | 0.204646 |
| 866 | hsa-miR-9-3p | 0.204464 |
| 867 | hsa-miR-4520-5p | 0.204398 |
| 868 | hsa-miR-503-3p | 0.204208 |
| 869 | hsa-miR-202-5p | 0.203172 |
| 870 | hsa-miR-223-5p | 0.200442 |
| 871 | hsa-miR-887-5p | 0.199883 |

| | | |
|---|---|---|
| 872 | hsa-miR-133b | 0.199022 |
| 873 | hsa-miR-3132 | 0.197424 |
| 874 | hsa-miR-627-3p | 0.196166 |
| 875 | hsa-miR-2467-5p | 0.195815 |
| 876 | hsa-miR-518e-3p | 0.195781 |
| 877 | hsa-miR-6889-3p | 0.195491 |
| 878 | hsa-miR-4639-3p | 0.193334 |
| 879 | hsa-miR-6511a-5p | 0.192021 |
| 880 | hsa-miR-6748-3p | 0.191233 |
| 881 | hsa-miR-6513-5p | 0.191209 |
| 882 | hsa-miR-4467 | 0.190802 |
| 883 | hsa-miR-1249-5p | 0.190292 |
| 884 | hsa-miR-497-3p | 0.187682 |
| 885 | hsa-miR-6734-5p | 0.186616 |
| 886 | hsa-miR-6804-3p | 0.182892 |
| 887 | hsa-miR-4661-5p | 0.182363 |
| 888 | hsa-miR-6764-3p | 0.182187 |
| 889 | hsa-miR-597-3p | 0.182089 |
| 890 | hsa-miR-490-3p | 0.181676 |
| 891 | hsa-miR-3194-3p | 0.181323 |
| 892 | hsa-miR-3156-5p | 0.180724 |
| 893 | hsa-miR-4517 | 0.180693 |
| 894 | hsa-miR-6751-5p | 0.177664 |
| 895 | hsa-miR-2278 | 0.177574 |
| 896 | hsa-miR-5094 | 0.176833 |
| 897 | hsa-miR-548ar-3p | 0.176437 |
| 898 | hsa-miR-4474-3p | 0.176345 |
| 899 | hsa-miR-556-5p | 0.175775 |
| 900 | hsa-miR-3150a-5p | 0.175116 |
| 901 | hsa-miR-6750-5p | 0.174631 |
| 902 | hsa-miR-133a-5p | 0.17437 |
| 903 | hsa-miR-181b-2-3p | 0.173371 |
| 904 | hsa-miR-520a-5p | 0.173368 |
| 905 | hsa-miR-4757-3p | 0.173299 |
| 906 | hsa-miR-3180-3p | 0.172246 |
| 907 | hsa-miR-4512 | 0.172243 |
| 908 | hsa-miR-6873-3p | 0.171959 |
| 909 | hsa-miR-3144-5p | 0.17156 |
| 910 | hsa-miR-4697-3p | 0.170909 |
| 911 | hsa-miR-7976 | 0.170865 |
| 912 | hsa-miR-5092 | 0.170834 |
| 913 | hsa-miR-4647 | 0.1705 |
| 914 | hsa-miR-6847-5p | 0.169943 |
| 915 | hsa-miR-3916 | 0.169423 |
| 916 | hsa-miR-3162-3p | 0.168035 |

| | | |
|---|---|---|
| 917 | hsa-miR-3685 | 0.167688 |
| 918 | hsa-miR-4750-5p | 0.167452 |
| 919 | hsa-miR-6806-3p | 0.16706 |
| 920 | hsa-miR-6787-3p | 0.167016 |
| 921 | hsa-miR-6813-5p | 0.166329 |
| 922 | hsa-miR-3129-3p | 0.165317 |
| 923 | hsa-miR-320e | 0.16527 |
| 924 | hsa-miR-4780 | 0.164538 |
| 925 | hsa-miR-3938 | 0.163468 |
| 926 | hsa-miR-3157-5p | 0.163441 |
| 927 | hsa-miR-6733-3p | 0.160213 |
| 928 | hsa-miR-374c-5p | 0.159647 |
| 929 | hsa-miR-6770-5p | 0.159646 |
| 930 | hsa-miR-6882-5p | 0.159576 |
| 931 | hsa-miR-15a-3p | 0.158251 |
| 932 | hsa-miR-508-3p | 0.158167 |
| 933 | hsa-miR-6734-3p | 0.156261 |
| 934 | hsa-miR-4536-3p | 0.156069 |
| 935 | hsa-miR-6793-3p | 0.155513 |
| 936 | hsa-miR-548ai;hsa-miR-570-5p | 0.155382 |
| 937 | hsa-miR-3924 | 0.154709 |
| 938 | hsa-miR-4667-3p | 0.153682 |
| 939 | hsa-miR-4433b-5p | 0.153591 |
| 940 | hsa-miR-6780a-5p | 0.153378 |
| 941 | hsa-miR-4749-5p | 0.152888 |
| 942 | hsa-miR-6776-3p | 0.151884 |
| 943 | hsa-miR-2467-3p | 0.150047 |
| 944 | hsa-miR-4646-5p | 0.149213 |
| 945 | hsa-miR-4746-5p | 0.148675 |
| 946 | hsa-miR-6814-3p | 0.148085 |
| 947 | hsa-miR-520d-3p | 0.147598 |
| 948 | hsa-miR-7109-3p | 0.147149 |
| 949 | hsa-miR-146a-3p | 0.146286 |
| 950 | hsa-miR-892a | 0.145203 |
| 951 | hsa-miR-4645-3p | 0.144949 |
| 952 | hsa-miR-6858-3p | 0.143531 |
| 953 | hsa-miR-1269a | 0.142858 |
| 954 | hsa-miR-4701-5p | 0.142326 |
| 955 | hsa-miR-4473 | 0.14205 |
| 956 | hsa-miR-585-3p | 0.141422 |
| 957 | hsa-miR-548ah-3p | 0.141014 |
| 958 | hsa-miR-1913 | 0.140384 |
| 959 | hsa-miR-34b-5p | 0.140047 |
| 960 | hsa-miR-6735-3p | 0.139988 |
| 961 | hsa-miR-6751-3p | 0.139723 |

| | | |
|---|---|---|
| 962 | hsa-miR-378g | 0.139285 |
| 963 | hsa-miR-6769a-5p | 0.139049 |
| 964 | hsa-miR-7112-3p | 0.137811 |
| 965 | hsa-miR-4638-3p | 0.136952 |
| 966 | hsa-miR-7158-3p | 0.136512 |
| 967 | hsa-let-7a-2-3p | 0.136396 |
| 968 | hsa-miR-3934-3p | 0.13609 |
| 969 | hsa-miR-1273c | 0.136061 |
| 970 | hsa-miR-6740-3p | 0.135757 |
| 971 | hsa-miR-525-5p | 0.135448 |
| 972 | hsa-miR-7703 | 0.135406 |
| 973 | hsa-miR-6823-3p | 0.133961 |
| 974 | hsa-miR-548ak | 0.133796 |
| 975 | hsa-miR-96-3p | 0.133353 |
| 976 | hsa-miR-135b-3p | 0.132661 |
| 977 | hsa-miR-1537-3p | 0.131501 |
| 978 | hsa-miR-3617-3p | 0.130099 |
| 979 | hsa-miR-378d | 0.128599 |
| 980 | hsa-miR-548an | 0.127387 |
| 981 | hsa-miR-4762-5p | 0.126614 |
| 982 | hsa-miR-6780b-3p | 0.126279 |
| 983 | hsa-miR-6845-5p | 0.126226 |
| 984 | hsa-miR-7855-5p | 0.125232 |
| 985 | hsa-miR-150-3p | 0.124578 |
| 986 | hsa-miR-6754-3p | 0.124198 |
| 987 | hsa-miR-4458 | 0.123902 |
| 988 | hsa-miR-4524a-5p | 0.12381 |
| 989 | hsa-miR-511-3p | 0.123799 |
| 990 | hsa-miR-6746-3p | 0.123446 |
| 991 | hsa-miR-6879-3p | 0.123213 |
| 992 | hsa-miR-2115-3p | 0.123143 |
| 993 | hsa-miR-4766-3p | 0.122759 |
| 994 | hsa-miR-5682 | 0.122112 |
| 995 | hsa-miR-6840-5p | 0.122075 |
| 996 | hsa-miR-4668-3p | 0.121997 |
| 997 | hsa-miR-4760-5p | 0.121997 |
| 998 | hsa-miR-6752-3p | 0.121499 |
| 999 | hsa-miR-6884-3p | 0.121131 |
| 1000 | hsa-miR-6804-5p | 0.121029 |
| 1001 | hsa-miR-7108-3p | 0.120065 |
| 1002 | hsa-miR-579-5p | 0.119655 |
| 1003 | hsa-miR-3188 | 0.119304 |
| 1004 | hsa-miR-6892-5p | 0.119244 |
| 1005 | hsa-miR-6864-5p | 0.118932 |
| 1006 | hsa-miR-3614-5p | 0.11785 |

| | | |
|---|---|---|
| 1007 | hsa-miR-6732-5p | 0.117105 |
| 1008 | hsa-miR-561-3p | 0.116886 |
| 1009 | hsa-miR-4429 | 0.116767 |
| 1010 | hsa-miR-3180 | 0.114831 |
| 1011 | hsa-miR-1236-3p | 0.113644 |
| 1012 | hsa-miR-6784-3p | 0.112611 |
| 1013 | hsa-miR-6761-5p | 0.112386 |
| 1014 | hsa-miR-3677-3p | 0.112007 |
| 1015 | hsa-miR-5002-5p | 0.109796 |
| 1016 | hsa-miR-579-3p | 0.109475 |
| 1017 | hsa-miR-1914-3p | 0.109413 |
| 1018 | hsa-miR-203a-5p | 0.108403 |
| 1019 | hsa-miR-4741 | 0.108386 |
| 1020 | hsa-miR-6756-3p | 0.107422 |
| 1021 | hsa-miR-599 | 0.106762 |
| 1022 | hsa-miR-6815-5p | 0.106646 |
| 1023 | hsa-miR-6730-5p | 0.105988 |
| 1024 | hsa-miR-4743-3p | 0.10527 |
| 1025 | hsa-miR-5090 | 0.10527 |
| 1026 | hsa-miR-6857-3p | 0.104919 |
| 1027 | hsa-miR-4677-5p | 0.104901 |
| 1028 | hsa-miR-4640-5p | 0.1049 |
| 1029 | hsa-miR-4687-3p | 0.104506 |
| 1030 | hsa-miR-5193 | 0.104286 |
| 1031 | hsa-miR-6811-5p | 0.104187 |
| 1032 | hsa-miR-4999-5p | 0.102824 |
| 1033 | hsa-miR-7975 | 0.10276 |
| 1034 | hsa-miR-6801-3p | 0.102076 |
| 1035 | hsa-miR-1915-5p | 0.101444 |
| 1036 | hsa-miR-3064-3p | 0.100799 |
| 1037 | hsa-miR-548y | 0.100799 |
| 1038 | hsa-miR-6895-5p | 0.100332 |
| 1039 | hsa-miR-4660 | 0.100011 |
| 1040 | hsa-miR-4781-5p | 0.099216 |
| 1041 | hsa-miR-6817-3p | 0.098265 |
| 1042 | hsa-miR-6503-3p | 0.098237 |
| 1043 | hsa-miR-2276-5p | 0.097179 |
| 1044 | hsa-miR-6858-5p | 0.097168 |
| 1045 | hsa-miR-939-3p | 0.096861 |
| 1046 | hsa-miR-3177-5p | 0.096142 |
| 1047 | hsa-miR-5582-3p | 0.095778 |
| 1048 | hsa-miR-3175 | 0.094724 |
| 1049 | hsa-miR-6502-5p | 0.094709 |
| 1050 | hsa-miR-6870-3p | 0.094325 |
| 1051 | hsa-miR-4422 | 0.093298 |

| | | |
|---|---|---|
| 1052 | hsa-miR-6855-5p | 0.092699 |
| 1053 | hsa-miR-5001-5p | 0.092564 |
| 1054 | hsa-miR-2682-3p | 0.092259 |
| 1055 | hsa-miR-548h-5p | 0.09225 |
| 1056 | hsa-miR-6513-3p | 0.091891 |
| 1057 | hsa-miR-6890-3p | 0.091588 |
| 1058 | hsa-miR-3124-5p | 0.091497 |
| 1059 | hsa-miR-4745-3p | 0.091494 |
| 1060 | hsa-miR-1915-3p | 0.091492 |
| 1061 | hsa-miR-7843-3p | 0.091492 |
| 1062 | hsa-miR-548ax | 0.090179 |
| 1063 | hsa-miR-4769-3p | 0.089856 |
| 1064 | hsa-miR-515-5p | 0.089784 |
| 1065 | hsa-miR-6878-3p | 0.089518 |
| 1066 | hsa-miR-3622a-3p | 0.089507 |
| 1067 | hsa-miR-302a-3p | 0.089436 |
| 1068 | hsa-miR-4651 | 0.089436 |
| 1069 | hsa-miR-6877-5p | 0.08905 |
| 1070 | hsa-miR-371a-3p | 0.088027 |
| 1071 | hsa-miR-6740-5p | 0.087412 |
| 1072 | hsa-miR-3116 | 0.087379 |
| 1073 | hsa-miR-552-3p | 0.087015 |
| 1074 | hsa-miR-3183 | 0.085949 |
| 1075 | hsa-miR-653-5p | 0.084965 |
| 1076 | hsa-miR-4524a-3p | 0.084845 |
| 1077 | hsa-miR-6499-5p | 0.084247 |
| 1078 | hsa-miR-1273d | 0.084205 |
| 1079 | hsa-miR-5690 | 0.083863 |
| 1080 | hsa-miR-4515 | 0.082461 |
| 1081 | hsa-miR-499b-3p | 0.082124 |
| 1082 | hsa-miR-3167 | 0.082096 |
| 1083 | hsa-miR-4686 | 0.082096 |
| 1084 | hsa-miR-211-5p | 0.081924 |
| 1085 | hsa-miR-6792-3p | 0.081711 |
| 1086 | hsa-miR-4750-3p | 0.081418 |
| 1087 | hsa-miR-1237-5p | 0.081331 |
| 1088 | hsa-miR-202-3p | 0.081331 |
| 1089 | hsa-miR-6763-5p | 0.081331 |
| 1090 | hsa-miR-7156-5p | 0.081331 |
| 1091 | hsa-miR-6851-3p | 0.081329 |
| 1092 | hsa-miR-6759-5p | 0.081112 |
| 1093 | hsa-miR-6877-3p | 0.081112 |
| 1094 | hsa-miR-6884-5p | 0.081112 |
| 1095 | hsa-miR-4707-3p | 0.081037 |
| 1096 | hsa-miR-6889-5p | 0.081027 |

| | | |
|---|---|---|
| 1097 | hsa-miR-6820-3p | 0.080013 |
| 1098 | hsa-miR-1252-5p | 0.08 |
| 1099 | hsa-miR-3182 | 0.079659 |
| 1100 | hsa-miR-7845-5p | 0.078196 |
| 1101 | hsa-miR-4680-3p | 0.077962 |
| 1102 | hsa-miR-149-3p | 0.077931 |
| 1103 | hsa-miR-7851-3p | 0.077905 |
| 1104 | hsa-miR-6814-5p | 0.07751 |
| 1105 | hsa-miR-526b-5p | 0.077245 |
| 1106 | hsa-miR-548ay-5p | 0.076822 |
| 1107 | hsa-miR-5588-3p | 0.076527 |
| 1108 | hsa-miR-5004-5p | 0.076484 |
| 1109 | hsa-miR-6865-5p | 0.07644 |
| 1110 | hsa-miR-6501-3p | 0.075824 |
| 1111 | hsa-miR-3690 | 0.075487 |
| 1112 | hsa-miR-6789-5p | 0.075487 |
| 1113 | hsa-miR-4482-3p | 0.075428 |
| 1114 | hsa-miR-450a-2-3p | 0.075062 |
| 1115 | hsa-miR-1290 | 0.074677 |
| 1116 | hsa-miR-6826-5p | 0.074006 |
| 1117 | hsa-miR-1294 | 0.073715 |
| 1118 | hsa-miR-6830-3p | 0.073715 |
| 1119 | hsa-miR-1305 | 0.073697 |
| 1120 | hsa-miR-4668-5p | 0.072953 |
| 1121 | hsa-miR-4523 | 0.07295 |
| 1122 | hsa-miR-5700 | 0.072618 |
| 1123 | hsa-miR-1255b-5p | 0.071999 |
| 1124 | hsa-miR-4654 | 0.071999 |
| 1125 | hsa-miR-3121-3p | 0.071549 |
| 1126 | hsa-miR-3910 | 0.071549 |
| 1127 | hsa-miR-3920 | 0.071549 |
| 1128 | hsa-miR-3941 | 0.071549 |
| 1129 | hsa-miR-578 | 0.071549 |
| 1130 | hsa-miR-6881-3p | 0.071549 |
| 1131 | hsa-miR-3692-3p | 0.071161 |
| 1132 | hsa-miR-548aw | 0.070565 |
| 1133 | hsa-miR-548l | 0.070565 |
| 1134 | hsa-miR-3664-5p | 0.069847 |
| 1135 | hsa-miR-518b | 0.069525 |
| 1136 | hsa-miR-6515-3p | 0.069525 |
| 1137 | hsa-miR-6836-5p | 0.068748 |
| 1138 | hsa-miR-4727-3p | 0.068427 |
| 1139 | hsa-miR-3189-3p | 0.068413 |
| 1140 | hsa-miR-3659 | 0.068413 |
| 1141 | hsa-miR-4706 | 0.068413 |

| | | |
|---|---|---|
| 1142 | hsa-miR-6501-5p | 0.068413 |
| 1143 | hsa-miR-3136-5p | 0.068061 |
| 1144 | hsa-miR-4747-3p | 0.068061 |
| 1145 | hsa-miR-548at-3p | 0.067344 |
| 1146 | hsa-miR-6795-3p | 0.067035 |
| 1147 | hsa-miR-3181 | 0.067006 |
| 1148 | hsa-miR-4664-3p | 0.066374 |
| 1149 | hsa-miR-4733-5p | 0.066272 |
| 1150 | hsa-miR-548az-3p | 0.066272 |
| 1151 | hsa-miR-6871-5p | 0.066272 |
| 1152 | hsa-miR-1228-3p | 0.06526 |
| 1153 | hsa-miR-3152-5p | 0.06526 |
| 1154 | hsa-miR-500b-3p | 0.06526 |
| 1155 | hsa-miR-767-3p | 0.06526 |
| 1156 | hsa-miR-6764-5p | 0.065229 |
| 1157 | hsa-miR-7113-3p | 0.064574 |
| 1158 | hsa-miR-124-3p | 0.063827 |
| 1159 | hsa-miR-3115 | 0.063531 |
| 1160 | hsa-miR-3130-5p | 0.062815 |
| 1161 | hsa-miR-4455 | 0.062784 |
| 1162 | hsa-miR-933 | 0.061762 |
| 1163 | hsa-miR-4709-3p | 0.061087 |
| 1164 | hsa-miR-1292-3p | 0.060995 |
| 1165 | hsa-miR-3688-3p | 0.060995 |
| 1166 | hsa-miR-499a-3p | 0.060995 |
| 1167 | hsa-miR-372-3p | 0.059328 |
| 1168 | hsa-miR-6780a-3p | 0.059284 |
| 1169 | hsa-miR-3131 | 0.057937 |
| 1170 | hsa-miR-605-5p | 0.057937 |
| 1171 | hsa-miR-6755-5p | 0.057863 |
| 1172 | hsa-miR-345-3p | 0.057599 |
| 1173 | hsa-miR-4712-5p | 0.057599 |
| 1174 | hsa-miR-548as-5p | 0.057599 |
| 1175 | hsa-miR-92a-2-5p | 0.057599 |
| 1176 | hsa-miR-1225-5p | 0.057189 |
| 1177 | hsa-miR-6515-5p | 0.056882 |
| 1178 | hsa-miR-934 | 0.056135 |
| 1179 | hsa-miR-1827 | 0.05583 |
| 1180 | hsa-miR-4435 | 0.055448 |
| 1181 | hsa-miR-4785 | 0.055448 |
| 1182 | hsa-miR-6825-3p | 0.055448 |
| 1183 | hsa-miR-490-5p | 0.055094 |
| 1184 | hsa-miR-944 | 0.055063 |
| 1185 | hsa-miR-3135a | 0.054787 |
| 1186 | hsa-miR-105-3p | 0.05473 |

| | | |
|---|---|---|
| 1187 | hsa-miR-3118 | 0.05473 |
| 1188 | hsa-miR-4433b-3p | 0.05473 |
| 1189 | hsa-miR-5187-5p | 0.05473 |
| 1190 | hsa-miR-219b-3p | 0.054348 |
| 1191 | hsa-miR-450a-1-3p | 0.054348 |
| 1192 | hsa-miR-4665-5p | 0.054346 |
| 1193 | hsa-miR-4536-5p | 0.053672 |
| 1194 | hsa-miR-4798-5p | 0.053662 |
| 1195 | hsa-miR-508-5p | 0.053662 |
| 1196 | hsa-miR-520a-3p | 0.053662 |
| 1197 | hsa-miR-6738-3p | 0.053662 |
| 1198 | hsa-miR-6815-3p | 0.053662 |
| 1199 | hsa-miR-4511 | 0.052649 |
| 1200 | hsa-miR-548u | 0.052648 |
| 1201 | hsa-miR-3126-5p | 0.052253 |
| 1202 | hsa-miR-3160-3p | 0.052253 |
| 1203 | hsa-miR-6729-3p | 0.052251 |
| 1204 | hsa-miR-3612 | 0.051975 |
| 1205 | hsa-miR-18b-3p | 0.050831 |
| 1206 | hsa-miR-3691-5p | 0.050831 |
| 1207 | hsa-miR-548n | 0.050831 |
| 1208 | hsa-miR-3913-3p | 0.050829 |
| 1209 | hsa-miR-4424 | 0.050829 |
| 1210 | hsa-miR-518f-3p | 0.050829 |
| 1211 | hsa-miR-6812-5p | 0.050829 |
| 1212 | hsa-miR-371a-5p | 0.050553 |
| 1213 | hsa-miR-1283 | 0.049118 |
| 1214 | hsa-miR-301b-5p | 0.048414 |
| 1215 | hsa-miR-4725-5p | 0.048385 |
| 1216 | hsa-miR-676-5p | 0.047697 |
| 1217 | hsa-miR-1910-5p | 0.046687 |
| 1218 | hsa-miR-3139 | 0.046687 |
| 1219 | hsa-miR-581 | 0.046687 |
| 1220 | hsa-miR-6736-3p | 0.046687 |
| 1221 | hsa-miR-3129-5p | 0.04635 |
| 1222 | hsa-miR-3927-3p | 0.04635 |
| 1223 | hsa-miR-4635 | 0.04635 |
| 1224 | hsa-miR-4772-3p | 0.04635 |
| 1225 | hsa-miR-5006-5p | 0.04635 |
| 1226 | hsa-miR-519a-3p | 0.04635 |
| 1227 | hsa-miR-7113-5p | 0.04635 |
| 1228 | hsa-miR-4695-3p | 0.04597 |
| 1229 | hsa-miR-4526 | 0.044928 |
| 1230 | hsa-miR-5009-5p | 0.044897 |
| 1231 | hsa-miR-517c-3p | 0.044897 |

| | | |
|---|---|---|
| 1232 | hsa-miR-1286 | 0.04418 |
| 1233 | hsa-miR-3918 | 0.04418 |
| 1234 | hsa-miR-4421 | 0.043874 |
| 1235 | hsa-miR-4659a-5p | 0.043506 |
| 1236 | hsa-miR-549a | 0.043506 |
| 1237 | hsa-miR-1267 | 0.0432 |
| 1238 | hsa-miR-3178 | 0.0432 |
| 1239 | hsa-miR-3179 | 0.0432 |
| 1240 | hsa-miR-3684 | 0.0432 |
| 1241 | hsa-miR-4712-3p | 0.0432 |
| 1242 | hsa-miR-4733-3p | 0.0432 |
| 1243 | hsa-miR-4735-5p | 0.0432 |
| 1244 | hsa-miR-4739 | 0.0432 |
| 1245 | hsa-miR-523-3p | 0.0432 |
| 1246 | hsa-miR-5697 | 0.0432 |
| 1247 | hsa-miR-6772-5p | 0.0432 |
| 1248 | hsa-miR-6861-5p | 0.0432 |
| 1249 | hsa-miR-6871-3p | 0.0432 |
| 1250 | hsa-miR-3124-3p | 0.042482 |
| 1251 | hsa-miR-4792 | 0.042453 |
| 1252 | hsa-miR-1229-5p | 0.041062 |
| 1253 | hsa-miR-3130-3p | 0.041062 |
| 1254 | hsa-miR-4420 | 0.041048 |
| 1255 | hsa-miR-4754 | 0.041048 |
| 1256 | hsa-miR-6850-5p | 0.041048 |
| 1257 | hsa-miR-6856-5p | 0.041048 |
| 1258 | hsa-miR-7108-5p | 0.041048 |
| 1259 | hsa-miR-1225-3p | 0.040666 |
| 1260 | hsa-miR-1537-5p | 0.040666 |
| 1261 | hsa-miR-20b-3p | 0.040666 |
| 1262 | hsa-miR-3120-3p | 0.040666 |
| 1263 | hsa-miR-383-3p | 0.040666 |
| 1264 | hsa-miR-3940-5p | 0.040666 |
| 1265 | hsa-miR-4463 | 0.040666 |
| 1266 | hsa-miR-4634 | 0.040666 |
| 1267 | hsa-miR-4726-3p | 0.040666 |
| 1268 | hsa-miR-4793-3p | 0.040666 |
| 1269 | hsa-miR-5194 | 0.040666 |
| 1270 | hsa-miR-519b-3p | 0.040666 |
| 1271 | hsa-miR-544b | 0.040666 |
| 1272 | hsa-miR-548av-5p | 0.040666 |
| 1273 | hsa-miR-5581-5p | 0.040666 |
| 1274 | hsa-miR-573 | 0.040666 |
| 1275 | hsa-miR-605-3p | 0.040666 |
| 1276 | hsa-miR-6720-3p | 0.040666 |

| | | |
|---|---|---|
| 1277 | hsa-miR-6748-5p | 0.040666 |
| 1278 | hsa-miR-6768-5p | 0.040666 |
| 1279 | hsa-miR-6775-5p | 0.040666 |
| 1280 | hsa-miR-6803-5p | 0.040666 |
| 1281 | hsa-miR-6845-3p | 0.040666 |
| 1282 | hsa-miR-7978 | 0.040666 |
| 1283 | hsa-miR-4417 | 0.040663 |
| 1284 | hsa-miR-6788-3p | 0.040663 |
| 1285 | hsa-miR-6856-3p | 0.040663 |
| 1286 | hsa-miR-6891-5p | 0.040663 |
| 1287 | hsa-miR-939-5p | 0.040387 |
| 1288 | hsa-miR-6727-3p | 0.03967 |
| 1289 | hsa-miR-3173-3p | 0.038219 |
| 1290 | hsa-miR-1257 | 0.037575 |
| 1291 | hsa-miR-6865-3p | 0.037575 |
| 1292 | hsa-miR-6805-3p | 0.037531 |
| 1293 | hsa-miR-6503-5p | 0.036857 |
| 1294 | hsa-miR-1304-5p | 0.035774 |
| 1295 | hsa-miR-6727-5p | 0.035774 |
| 1296 | hsa-miR-6741-5p | 0.035774 |
| 1297 | hsa-miR-6774-3p | 0.035774 |
| 1298 | hsa-miR-6798-5p | 0.035774 |
| 1299 | hsa-miR-6834-3p | 0.035774 |
| 1300 | hsa-miR-6859-3p | 0.035774 |
| 1301 | hsa-miR-1228-5p | 0.034762 |
| 1302 | hsa-miR-3646 | 0.034762 |
| 1303 | hsa-miR-4431 | 0.034762 |
| 1304 | hsa-miR-4800-5p | 0.034762 |
| 1305 | hsa-miR-5189-5p | 0.034762 |
| 1306 | hsa-miR-6508-5p | 0.034762 |
| 1307 | hsa-miR-6728-3p | 0.034762 |
| 1308 | hsa-miR-6842-5p | 0.034762 |
| 1309 | hsa-miR-147a | 0.033883 |
| 1310 | hsa-miR-520b | 0.033159 |
| 1311 | hsa-miR-520c-3p | 0.033159 |
| 1312 | hsa-miR-422a | 0.032616 |
| 1313 | hsa-miR-4804-5p | 0.032287 |
| 1314 | hsa-miR-6130 | 0.032287 |
| 1315 | hsa-miR-3960 | 0.031919 |
| 1316 | hsa-miR-4446-5p | 0.031919 |
| 1317 | hsa-miR-6824-3p | 0.031919 |
| 1318 | hsa-miR-504-3p | 0.03157 |
| 1319 | hsa-miR-6882-3p | 0.03157 |
| 1320 | hsa-miR-130a-5p | 0.030497 |
| 1321 | hsa-miR-4709-5p | 0.030497 |

| | | |
|---|---|---|
| 1322 | hsa-miR-551a | 0.030497 |
| 1323 | hsa-miR-6759-3p | 0.030497 |
| 1324 | hsa-miR-6785-5p | 0.030497 |
| 1325 | hsa-miR-4761-3p | 0.029475 |
| 1326 | hsa-miR-6886-3p | 0.029475 |
| 1327 | hsa-miR-3158-5p | 0.0288 |
| 1328 | hsa-miR-4504 | 0.0288 |
| 1329 | hsa-miR-4679 | 0.0288 |
| 1330 | hsa-miR-4716-3p | 0.0288 |
| 1331 | hsa-miR-5009-3p | 0.0288 |
| 1332 | hsa-miR-6782-3p | 0.0288 |
| 1333 | hsa-miR-6875-3p | 0.0288 |
| 1334 | hsa-miR-585-5p | 0.028082 |
| 1335 | hsa-miR-519d-3p | 0.028053 |
| 1336 | hsa-miR-3680-5p | 0.027365 |
| 1337 | hsa-miR-4717-3p | 0.027365 |
| 1338 | hsa-miR-5698 | 0.027365 |
| 1339 | hsa-miR-586 | 0.027365 |
| 1340 | hsa-miR-6758-5p | 0.027365 |
| 1341 | hsa-miR-6781-5p | 0.027365 |
| 1342 | hsa-miR-6797-3p | 0.027365 |
| 1343 | hsa-miR-6888-5p | 0.027365 |
| 1344 | hsa-miR-300 | 0.025951 |
| 1345 | hsa-miR-6761-3p | 0.02527 |
| 1346 | hsa-miR-6800-3p | 0.02527 |
| 1347 | hsa-miR-6834-5p | 0.02527 |
| 1348 | hsa-miR-6862-3p | 0.02527 |
| 1349 | hsa-miR-3619-3p | 0.024566 |
| 1350 | hsa-miR-4753-5p | 0.024566 |
| 1351 | hsa-miR-218-1-3p | 0.023848 |
| 1352 | hsa-miR-4306 | 0.023848 |
| 1353 | hsa-miR-6849-3p | 0.023848 |
| 1354 | hsa-miR-302b-3p | 0.023175 |
| 1355 | hsa-miR-3677-5p | 0.023175 |
| 1356 | hsa-miR-4680-5p | 0.023175 |
| 1357 | hsa-miR-4694-3p | 0.023175 |
| 1358 | hsa-miR-4748 | 0.023175 |
| 1359 | hsa-miR-6766-3p | 0.023175 |
| 1360 | hsa-miR-6780b-5p | 0.023175 |
| 1361 | hsa-miR-6821-5p | 0.023175 |
| 1362 | hsa-miR-6854-5p | 0.023175 |
| 1363 | hsa-miR-374c-3p | 0.021753 |
| 1364 | hsa-miR-550b-2-5p | 0.021753 |
| 1365 | hsa-miR-1909-5p | 0.020332 |
| 1366 | hsa-miR-4440 | 0.020332 |

| | | |
|---|---|---|
| 1367 | hsa-miR-4755-3p | 0.020332 |
| 1368 | hsa-miR-548ah-5p | 0.020332 |
| 1369 | hsa-miR-6510-3p | 0.020332 |
| 1370 | hsa-miR-6799-3p | 0.020332 |
| 1371 | hsa-miR-6887-3p | 0.020332 |
| 1372 | hsa-miR-1243 | 0.017887 |
| 1373 | hsa-miR-3154 | 0.017887 |
| 1374 | hsa-miR-3679-3p | 0.017887 |
| 1375 | hsa-miR-4301 | 0.017887 |
| 1376 | hsa-miR-4477b | 0.017887 |
| 1377 | hsa-miR-4498 | 0.017887 |
| 1378 | hsa-miR-517-5p | 0.017887 |
| 1379 | hsa-miR-5590-5p | 0.017887 |
| 1380 | hsa-miR-6133 | 0.017887 |
| 1381 | hsa-miR-632 | 0.017887 |
| 1382 | hsa-miR-6723-5p | 0.017887 |
| 1383 | hsa-miR-6739-3p | 0.017887 |
| 1384 | hsa-miR-6809-5p | 0.017887 |
| 1385 | hsa-miR-6829-5p | 0.017887 |
| 1386 | hsa-miR-6852-3p | 0.017887 |
| 1387 | hsa-miR-6861-3p | 0.017887 |
| 1388 | hsa-miR-6872-3p | 0.017887 |
| 1389 | hsa-miR-6874-3p | 0.017887 |
| 1390 | hsa-miR-1245b-5p | 0.0144 |
| 1391 | hsa-miR-1271-3p | 0.0144 |
| 1392 | hsa-miR-3660 | 0.0144 |
| 1393 | hsa-miR-3691-3p | 0.0144 |
| 1394 | hsa-miR-3912-5p | 0.0144 |
| 1395 | hsa-miR-4459 | 0.0144 |
| 1396 | hsa-miR-4659a-3p | 0.0144 |
| 1397 | hsa-miR-509-3-5p | 0.0144 |
| 1398 | hsa-miR-519a-5p | 0.0144 |
| 1399 | hsa-miR-548ao-5p | 0.0144 |
| 1400 | hsa-miR-552-5p | 0.0144 |
| 1401 | hsa-miR-5579-5p | 0.0144 |
| 1402 | hsa-miR-588 | 0.0144 |
| 1403 | hsa-miR-597-5p | 0.0144 |
| 1404 | hsa-miR-6132 | 0.0144 |
| 1405 | hsa-miR-615-5p | 0.0144 |
| 1406 | hsa-miR-6506-5p | 0.0144 |
| 1407 | hsa-miR-6509-5p | 0.0144 |
| 1408 | hsa-miR-6716-5p | 0.0144 |
| 1409 | hsa-miR-6823-5p | 0.0144 |
| 1410 | hsa-miR-6829-3p | 0.0144 |
| 1411 | hsa-miR-6832-3p | 0.0144 |

| | | |
|---|---|---|
| 1412 | hsa-miR-6836-3p | 0.0144 |
| 1413 | hsa-miR-1287-3p | 0.013683 |
| 1414 | hsa-miR-2115-5p | 0.013683 |
| 1415 | hsa-miR-3133 | 0.013683 |
| 1416 | hsa-miR-3193 | 0.013683 |
| 1417 | hsa-miR-34c-3p | 0.013683 |
| 1418 | hsa-miR-3616-5p | 0.013683 |
| 1419 | hsa-miR-4676-3p | 0.013683 |
| 1420 | hsa-miR-4743-5p | 0.013683 |
| 1421 | hsa-miR-4761-5p | 0.013683 |
| 1422 | hsa-miR-4768-5p | 0.013683 |
| 1423 | hsa-miR-5189-3p | 0.013683 |
| 1424 | hsa-miR-522-3p | 0.013683 |
| 1425 | hsa-miR-548bb-3p | 0.013683 |
| 1426 | hsa-miR-5706 | 0.013683 |
| 1427 | hsa-miR-602 | 0.013683 |
| 1428 | hsa-miR-642b-5p | 0.013683 |
| 1429 | hsa-miR-6779-5p | 0.013683 |
| 1430 | hsa-miR-6809-3p | 0.013683 |
| 1431 | hsa-miR-6819-5p | 0.013683 |
| 1432 | hsa-miR-6831-3p | 0.013683 |
| 1433 | hsa-miR-6837-5p | 0.013683 |
| 1434 | hsa-miR-6892-3p | 0.013683 |
| 1435 | hsa-miR-181d-3p | 0.011587 |
| 1436 | hsa-miR-3143 | 0.011587 |
| 1437 | hsa-miR-3166 | 0.011587 |
| 1438 | hsa-miR-3678-5p | 0.011587 |
| 1439 | hsa-miR-4796-5p | 0.011587 |
| 1440 | hsa-miR-5004-3p | 0.011587 |
| 1441 | hsa-miR-520g-3p | 0.011587 |
| 1442 | hsa-miR-5579-3p | 0.011587 |
| 1443 | hsa-miR-5587-5p | 0.011587 |
| 1444 | hsa-miR-6773-5p | 0.011587 |
| 1445 | hsa-miR-6840-3p | 0.011587 |
| 1446 | hsa-miR-6848-5p | 0.011587 |
| 1447 | hsa-miR-6878-5p | 0.011587 |
| 1448 | hsa-miR-6890-5p | 0.011587 |
| 1449 | hsa-miR-7111-5p | 0.011587 |
| 1450 | hsa-miR-1238-3p | 0.010166 |
| 1451 | hsa-miR-1297 | 0.010166 |
| 1452 | hsa-miR-1909-3p | 0.010166 |
| 1453 | hsa-miR-3135b | 0.010166 |
| 1454 | hsa-miR-31-3p | 0.010166 |
| 1455 | hsa-miR-3939 | 0.010166 |
| 1456 | hsa-miR-4703-3p | 0.010166 |

| | | |
|---|---|---|
| 1457 | hsa-miR-4753-3p | 0.010166 |
| 1458 | hsa-miR-4757-5p | 0.010166 |
| 1459 | hsa-miR-4791 | 0.010166 |
| 1460 | hsa-miR-4804-3p | 0.010166 |
| 1461 | hsa-miR-489-5p | 0.010166 |
| 1462 | hsa-miR-513c-5p | 0.010166 |
| 1463 | hsa-miR-548am-5p | 0.010166 |
| 1464 | hsa-miR-6507-5p | 0.010166 |
| 1465 | hsa-miR-651-3p | 0.010166 |
| 1466 | hsa-miR-6757-5p | 0.010166 |
| 1467 | hsa-miR-6821-3p | 0.010166 |
| 1468 | hsa-miR-6867-3p | 0.010166 |
| 1469 | hsa-miR-6880-5p | 0.010166 |
| 1470 | hsa-miR-7151-3p | 0.010166 |

**Table SIV. INS-1 cell miRNA export to HDL. Fold change of INS-1-nHDL versus cf-nHDL from n=1 pool of 3 samples.**

| Feature | DEseq2 (BaseMean) | Fold Change | cf-nHDL (RPM) | INS-1-nHDL (RPM) |
|---|---|---|---|---|
| rno-miR-375-3p | 6132.951 | 56.03596 | 14.10 | 755.72 |
| rno-miR-16-5p | 1555.169 | 0.343913 | 151.78 | 49.91 |
| rno-miR-203a-3p | 1337.862 | 4.042332 | 34.80 | 134.52 |
| rno-miR-21-5p | 449.1478 | 0.899418 | 31.02 | 26.67 |
| rno-miR-10a-5p | 397.4127 | 0.338184 | 38.95 | 12.60 |
| rno-miR-22-3p | 336.4992 | 2.271046 | 13.49 | 29.30 |
| rno-miR-27b-3p | 302.4495 | 1.55808 | 15.54 | 23.10 |
| rno-miR-320-3p | 195.859 | 0.502728 | 17.10 | 8.22 |
| rno-miR-125b-2-3p | 183.0042 | 0.624578 | 14.78 | 8.82 |
| rno-miR-130a-3p | 172.2597 | 2.458554 | 6.53 | 15.36 |
| rno-miR-192-5p | 160.0844 | 8.294808 | 2.26 | 17.92 |
| rno-miR-27a-3p | 114.328 | 0.805963 | 8.33 | 6.40 |
| rno-miR-378a-3p | 109.3488 | 6.118584 | 2.01 | 11.79 |
| rno-miR-182 | 87.68644 | 13.49039 | 0.79 | 10.24 |
| rno-miR-92a-3p | 80.38319 | 2.139767 | 3.36 | 6.87 |
| rno-let-7i-5p | 61.54899 | 6.345834 | 1.10 | 6.67 |
| rno-miR-340-5p | 54.92498 | 8.076497 | 0.79 | 6.13 |
| rno-miR-26a-5p | 49.94882 | 1.384559 | 2.75 | 3.64 |
| rno-miR-141-3p | 48.69655 | 0.230763 | 5.19 | 1.15 |
| rno-let-7f-5p | 47.94302 | 6.922716 | 0.79 | 5.25 |
| rno-miR-30a-5p | 47.08364 | 9.114886 | 0.61 | 5.32 |
| rno-miR-25-3p | 42.78739 | 2.676803 | 1.53 | 3.91 |

| | | | | |
|---|---|---|---|---|
| rno-miR-30e-5p | 38.27605 | 1.93673 | 1.71 | 3.17 |
| rno-miR-103-3p | 34.37302 | 3.922887 | 0.93 | 3.46 |
| rno-miR-181a-5p | 27.31948 | 2.668148 | 0.98 | 2.49 |
| rno-let-7b-5p | 25.56475 | 17.30648 | 0.16 | 3.00 |
| rno-miR-423-5p | 25.38629 | 0.947768 | 1.71 | 1.55 |
| rno-miR-30d-5p | 25.13523 | 4.999737 | 0.55 | 2.63 |
| rno-let-7c-5p | 24.45498 | 3.776037 | 0.68 | 2.44 |
| rno-miR-1224 | 23.09415 | 269.6946 | 0.00 | 2.90 |
| rno-miR-423-3p | 23.02357 | 0.07525 | 2.81 | 0.20 |
| rno-miR-23a-3p | 19.47848 | 0.494492 | 1.71 | 0.84 |
| rno-miR-328a-3p | 19.26305 | 40.38068 | 0.06 | 2.36 |
| rno-miR-107-3p | 17.2223 | 4.285485 | 0.41 | 1.73 |
| rno-miR-92b-3p | 16.93596 | 2.307589 | 0.67 | 1.48 |
| rno-miR-23b-3p | 16.68557 | 0.629353 | 1.34 | 0.84 |
| rno-miR-148a-3p | 16.18446 | 0.198937 | 1.77 | 0.34 |
| rno-miR-664-2-5p | 16.1122 | 188.1568 | 0.00 | 0.00 |
| rno-miR-205 | 16.00532 | 0.432682 | 1.47 | 0.61 |
| rno-let-7d-3p | 14.89487 | 14.9988 | 0.12 | 1.75 |
| rno-miR-451-5p | 14.788 | 0.221891 | 1.59 | 0.34 |
| rno-miR-140-3p | 14.7517 | 6.922635 | 0.24 | 1.62 |
| rno-miR-93-5p | 14.3578 | 14.42192 | 0.12 | 1.68 |
| rno-miR-98-5p | 13.42683 | 156.7962 | 0.00 | 1.68 |
| rno-miR-151-3p | 12.67499 | 8.076356 | 0.18 | 1.41 |
| rno-miR-532-5p | 12.2811 | 25.382 | 0.06 | 1.48 |
| rno-miR-26b-5p | 12.10263 | 0.008164 | 1.59 | 0.00 |
| rno-let-7g-5p | 11.85191 | 0.157343 | 1.34 | 0.20 |
| rno-miR-125a-5p | 11.70874 | 0.048078 | 1.47 | 0.07 |
| rno-let-7a-5p | 10.81339 | 0.659325 | 0.87 | 0.55 |
| rno-miR-29a-3p | 10.5267 | 6.538005 | 0.18 | 1.15 |
| rno-miR-488-3p | 10.20439 | 119.1634 | 0.00 | 0.00 |
| rno-miR-142-5p | 9.667655 | 0.384611 | 0.92 | 0.34 |
| rno-miR-222-3p | 9.667317 | 112.8912 | 0.00 | 1.21 |
| rno-miR-128-3p | 9.59573 | 19.61329 | 0.06 | 1.15 |
| rno-miR-133a-3p | 9.41693 | 0.839138 | 0.67 | 0.54 |
| rno-miR-132-3p | 9.130244 | 106.6191 | 0.00 | 1.15 |
| rno-miR-30d-3p | 9.130244 | 106.6191 | 0.00 | 1.15 |
| rno-miR-326-3p | 9.130244 | 106.6191 | 0.00 | 1.15 |
| rno-miR-221-3p | 8.378409 | 4.999656 | 0.18 | 0.88 |
| rno-miR-106b-3p | 8.056098 | 94.07489 | 0.00 | 1.01 |
| rno-miR-421-3p | 8.056098 | 94.07489 | 0.00 | 1.01 |
| rno-miR-10b-5p | 7.877298 | 0.692293 | 0.61 | 0.40 |
| rno-miR-150-5p | 7.340225 | 0.576914 | 0.61 | 0.34 |
| rno-miR-101a-3p | 6.981951 | 81.53067 | 0.00 | 0.88 |
| rno-miR-186-5p | 6.516803 | 0.015162 | 0.85 | 0.00 |
| rno-miR-1839-5p | 6.444878 | 75.25857 | 0.00 | 0.81 |

| | | | | |
|---|---|---|---|---|
| rno-miR-191a-5p | 6.015355 | 1.15381 | 0.37 | 0.40 |
| rno-miR-409a-3p | 5.370732 | 62.7144 | 0.00 | 0.67 |
| rno-miR-101b-3p | 5.120345 | 0.019297 | 0.67 | 0.00 |
| rno-miR-129-5p | 4.833659 | 56.44233 | 0.00 | 0.61 |
| rno-miR-877 | 4.833659 | 56.44233 | 0.00 | 0.61 |
| rno-miR-143-3p | 4.618897 | 2.307558 | 0.18 | 0.40 |
| rno-miR-126a-5p | 4.54731 | 1.442251 | 0.24 | 0.34 |
| rno-miR-652-3p | 4.296585 | 50.17028 | 0.00 | 0.54 |
| rno-miR-183-5p | 3.759512 | 43.899 | 0.00 | 0.47 |
| rno-miR-127-3p | 3.723887 | 0.026534 | 0.49 | 0.00 |
| rno-miR-100-5p | 3.258401 | 0.030324 | 0.43 | 0.00 |
| rno-miR-130b-5p | 3.258401 | 0.030324 | 0.43 | 0.00 |
| rno-miR-223-3p | 3.258401 | 0.030324 | 0.43 | 0.00 |
| rno-let-7e-5p | 2.792915 | 0.035378 | 0.37 | 0.00 |
